# Analysis of the Effects of Five Factors Relevant to *In Vitro* Chondrogenesis of Human Mesenchymal Stem Cells Using Factorial Design and High Throughput mRNA-Profiling

**DOI:** 10.1371/journal.pone.0096615

**Published:** 2014-05-09

**Authors:** Rune B. Jakobsen, Esben Østrup, Xiaolan Zhang, Tarjei S. Mikkelsen, Jan E. Brinchmann

**Affiliations:** 1 Norwegian Center for Stem Cell Research, Oslo University Hospital, Rikshospitalet, Oslo, Norway; 2 Department of Biochemistry, Institute of Basic Medical Sciences, The Medical Faculty, University of Oslo, Oslo, Norway; 3 Department of Biomaterials, Institute of Clinical Dentistry, University of Oslo, Oslo, Norway; 4 Broad Institute, Cambridge, Massachusetts, United States of America; 5 Harvard Stem Cell Institute and Department of Stem Cell and Regenerative Biology, Harvard University, Cambridge, Massachusetts, United States of America; 6 Institute of Immunology, Oslo University Hospital, Rikshospitalet, Oslo, Norway; University of Wisconsin-Madison, United States of America

## Abstract

The in vitro process of chondrogenic differentiation of mesenchymal stem cells for tissue engineering has been shown to require three-dimensional culture along with the addition of differentiation factors to the culture medium. In general, this leads to a phenotype lacking some of the cardinal features of native articular chondrocytes and their extracellular matrix. The factors used vary, but regularly include members of the transforming growth factor β superfamily and dexamethasone, sometimes in conjunction with fibroblast growth factor 2 and insulin-like growth factor 1, however the use of soluble factors to induce chondrogenesis has largely been studied on a single factor basis. In the present study we combined a factorial quality-by-design experiment with high-throughput mRNA profiling of a customized chondrogenesis related gene set as a tool to study in vitro chondrogenesis of human bone marrow derived mesenchymal stem cells in alginate. 48 different conditions of transforming growth factor β 1, 2 and 3, bone morphogenetic protein 2, 4 and 6, dexamethasone, insulin-like growth factor 1, fibroblast growth factor 2 and cell seeding density were included in the experiment. The analysis revealed that the best of the tested differentiation cocktails included transforming growth factor β 1 and dexamethasone. Dexamethasone acted in synergy with transforming growth factor β 1 by increasing many chondrogenic markers while directly downregulating expression of the pro-osteogenic gene osteocalcin. However, all factors beneficial to the expression of desirable hyaline cartilage markers also induced undesirable molecules, indicating that perfect chondrogenic differentiation is not achievable with the current differentiation protocols.

## Introduction

Mesenchymal stem cells (MSCs) have been advocated as a useful cell source for tissue engineering. MSCs were originally isolated from bone marrow, but have later been found in and isolated from numerous tissues [1], [2]. They can be readily expanded in vitro and differentiated into tissues of mesodermal and, in some instances, ectodermal lineages [3], [4]. Clinically MSCs have shown promising potential in treatments of graft-versus-host-disease and in repair of full-thickness cartilage defects [5], [6].

The in vitro process of directed differentiation of mesenchymal stem cells has been widely studied. Chondrogenic differentiation of MSCs has been shown to require the use of either high-density cell pellet, micro-mass cultures or a scaffold allowing for three-dimensional culture [7]–[9] along with the addition of differentiation factors to the culture medium [10]–[13].

The differentiation factors have traditionally included factors from the TGF superfamily such as transforming growth factor β (TGFβ) [7], [14], [15] and/or bone morphogenetic protein (BMP) [9], [16], [17] along with the steroid hormone dexamethasone (DEX). Other factors used are fibroblast growth factor 2 (FGF2) [15], [18], [19] and insulin-like growth factor 1 (IGF1) [20], [21]. Traditionally, the use of soluble factors to induce chondrogenesis has largely been studied on a single factor basis or with simple combinations of a few factors. However, optimizing differentiation conditions one factor at a time is time consuming, and does not take into account interdependency between factors, which is likely to play a role in growth factor mediated differentiation. Factorial analysis is commonly used in industrial processes as a statistically and scientifically sound way of analyzing interplay between several factors on a predefined outcome. Factorial design (often termed quality-by-design) has been used for optimization of protocols in a variety of industries and research areas including pharmaceutical studies and manufacturing, stem cell biology, polymer production and tissue engineering [22]–[24].

Previously, expression profiling of medium to large sets of genes on multiple samples has been done using microarray hybridization technology with a relative high cost per individual sample. Smaller sets of genes have often been investigated using quantitative polymerase chain reactions (qPCR), though upscaling of qPCR experiments rapidly exceeds practically and economically feasible numbers of reactions. However, the introduction of digital and highly multiplexed mRNA-profiling (Nanostring nCounter) has made it possible and cost-effective to analyze large number of samples on predefined gene sets of up to 800 genes with an accuracy equal to single-plex qPCR [25]. This may be performed directly on cell lysates, thus bypassing the variability introduced by RNA isolation and conversion to cDNA which is necessary in microarrays and RT-qPCR [26].

In the present study we undertook a detailed comparison of all possible combinations of five commonly used differentiation factors in a fully humanized culture system: TGFβ1, BMP2, dexamethasone, FGF2 and IGF1 used for in vitro chondrogenesis of MSCs established in 3D culture in alginate hydrogels, including a comparison of the three isoforms of the TFGβ growth factor (TGFβ1, TGFβ2 and TGFβ3) and three of the isoforms of BMP (BMP2, BMP4 and BMP6). Our aim was to explore factorial design and digital mRNA profiling as tools to characterize directed differentiation of MSCs and to validate the most commonly used chondrogenic growth factors.

## Methods and Materials

All chemicals were purchased from Sigma-Aldrich (St. Louis, MO) unless otherwise stated.

### Ethics statement

The study including the harvest of bone marrow from voluntary donors was approved by the Regional Committee for Medical Research Ethics, Southern Norway. Informed written consent was obtained from all donors before the harvest procedure.

### Cell harvest and culture

Bone marrow aspirates were obtained from the iliac crest of three healthy donors as previously described [27]. The isolation and culture procedure is given in Appendix S1.

### Medium and supplements

Growth medium for monolayer cultures contained 2 U/mL heparin, 100 U/mL penicillin, 100 µg/mL streptomycin and 2,5 µg/mL amphotericin B in DMEM F-12, with 20% human platelet lysate (hPL) (Appendix S1) added for the first passage and 10% for all subsequent passages.

Basic chondrogenic differentiation medium (bCDM) contained sodium pyruvate, ascorbic acid 2-phosphate, ITS and human serum albumin in high-glucose DMEM-F12 (4,5 g/L). bCDM was supplemented with BMP2, BMP4 or BMP6, TGFβ1, TGFβ2 or TGFβ3, dexamethasone, FGF2 and/or IGF1. Working concentrations and suppliers of all supplements are given in Table 1.

**Table 1:** List of culture supplements.

Supplements	Working concentration	Company	Catalog number
Sodium pyruvate	1 mM	Gibco (Life Technologies, Carlsbad, CA)	11360
Ascorbic acid 2-phosphate	0.1 mM	Sigma-Aldrich (St. Louis, MO)	A8960
Insulin-transferrin-sodium selenite media supplement	1%	Sigma-Aldrich (St. Louis, MO)	I1884
Human serum albumin	1,25 mg/mL	Baxter (Deerfield, IL)	N/A
Dexamethasone	100 nM	Sigma-Aldrich (St. Louis, MO)	D4902
Insulin-like growth factor 1	100 ng/mL	Sigma-Aldrich (St. Louis, MO)	I3769
Transforming growth factor β 1	10 ng/mL	R&D Systems (Minneapolis, MN)	240-B
Transforming growth factor β 2	10 ng/mL	R&D Systems (Minneapolis, MN)	302-B2
Transforming growth factor β 3	10 ng/mL	R&D Systems (Minneapolis, MN)	243-B3
Bone morphogenetic protein 2	500 ng/mL	Wyeth (Taplow, UK)	InductOs
Bone morphogenetic protein 4	500 ng/mL	R&D Systems (Minneapolis, MN)	314-BP
Bone morphogenetic protein 6	500 ng/mL	R&D Systems (Minneapolis, MN)	507-BP
Fibroblast growth factor 2	10 ng/mL	Sigma-Aldrich (St. Louis, MO)	F0291

### Validation of cells as MSC

Cells used for experiments in passage 2 or 3 were validated as MSCs by flow cytometry and differentiation assays as described in Appendix S1 and Table S1.

### 3D cell culture

Cells in passage 2 or 3 were trypsinized, counted, washed in PBS and seeded into a self-gelling alginate scaffold (NovaMatrix, Sandvika, Norway) as described previously and in Appendix S1 [28].

### Experimental design

**Figure 1:**
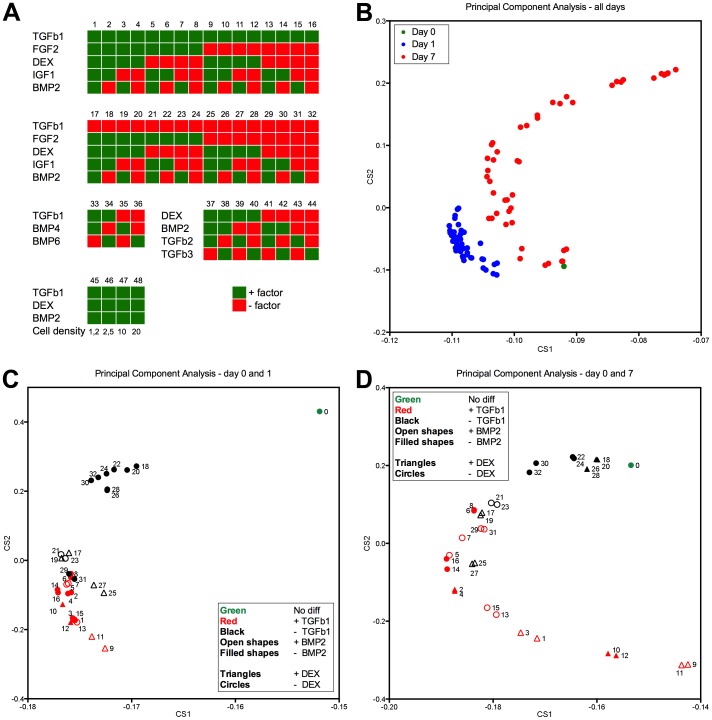
Experimental setup and principal component analysis (PCA). **A.** Experimental setup with numbering of the different conditions. When not stated, the cell density was 10^7^ cells per mL. **B.** PCA on all conditions labelled by days in culture. **C.** PCA limited to conditions 1–32 on days 0 and 1. **D.** PCA limited to conditions 1–32 on days 0 and day 7.

We investigated a total of 48 different conditions (Figure 1A). Five factors: TGFβ1, IGF1, DEX, FGF2 and BMP2 were investigated in two-level (present or not) full 2^5^ factorial design experiments. Concentrations were based on typical use in the literature [7], [15]–[17], [21]. This gave a total of 32 conditions in each experiment, which was repeated with cells from three donors at two time points each: days 1 and 7. In addition a modified design investigating TGFβ isoforms 1, 2 and 3 and BMP isoforms 2, 4 and 6 was also performed, including an experiment where the cell density was varied between 1.25×10^6^ and 2×10^7^ cells/mL at log(2)-intervals. Design of the experiments was done with MiniTAB Statistical Software version 16 (Minitab Inc, State College, PA - www.minitab.com). At the end of the experiments discs were divided in halves, snap-frozen in liquid nitrogen and stored at −80°C. Negative control disc cultures were performed in the same way using bCDM only, while positive controls were discs supplemented with our to date standard chondrogenic differentiation cocktail consisting of TGFβ1, BMP2 and DEX, concentrations are given in Table 1. Positive control samples were collected at day 7, 14 and 21 with additions of extra discs fixed in 4% paraformaldehyde for immunohistochemistry.

### Digital mRNA profiling and quantitative polymerase chain reaction

Frozen discs were crushed in liquid nitrogen with a pestle (Argos Technologies, Elgin, IL), lysed in RLT buffer and homogenized (QiaShredder, Qiagen, Venlo, Netherlands). Samples were then either directly used for digital mRNA profiling with the Nanostring nCounter technology [25] (NanoString Technologies, Seattle, WA) or RNA was extracted (RNeasy Mini kit, Qiagen). For qPCR, after DNase I treatment (Ambion; Life Technologies, Carlsbad, CA), reverse transcription (RT) was performed according to protocol (High-Capacity cDNA archive Kit; Applied Biosystems, Foster City, CA) using 200 ng total RNA per RT reaction and analyzed with the primers for peroxisome proliferator-activated receptor gamma and osteomodulin (*PPARG* Hs01115513_m1, *OMD* Hs00192325_m1, Applied Biosystems).

For the digital mRNA profiling, a custom chondrogenic gene set consisting of 364 genes (Appendix S2) including endogenous controls was established on the background of genes known or suspected to be affected by chondroskeletogenesis. We used a previous study from our lab of differentially expressed genes in the course of chondrogenic differentiation of MSCs to select genes based predominantly on the level of differential expression. These were supplemented with genes chosen from previously published papers from other labs describing genes known to be involved in chondrogenesis [28]–[31]. For analysis of lysate vs. purified mRNA performance, the pre-designed NanoString 48-plex Customer Assay Evaluation (CAE) kit was used instead. Sample preparation and hybridization was performed according to the manufacturer's instructions with either 100 ng of total RNA or lysate equivalent to 10.000 cells. All hybridizations were incubated at 65°C purified and counted on the nCounter Prep Station and Digital Analyzer (NanoString Technologies).

### Data analysis and statistics

Normalization for lane-to-lane variation and positive spike-in-control series were performed according to the manufacturer's protocol using Microsoft Excel (Microsoft, Redmond, WA) [32]. The geometric mean of the five best endogenous control genes identified by NormFinder was used to normalize the data [33]. Further data normalization was performed in the R statistical application (http://www.R-project.org/) including log transformation using the "vsn" package [34].

The MiniTAB Statistical Software package was used to fit a statistical regression model to analyze main effects, two and three factor interactions with significance assumed for p-values less than 0.05 in a multivariate analysis of variance on the normalized data. If needed, transformation of the responses was used to make the residuals exhibit normality as judged by normality plots. Pearson's correlation between expression values in lysates and RNA and Spearman's correlation between rankings of conditions day 1 and day 7 were calculated with Prism 6 (Graphpad, San Diego, CA).

To make graphical representations of wanted and unwanted genes the data were normalized by Studentization, ie. subtracting the mean expression of each gene across all conditions divided by the standard deviation. For analysis of significantly changed genes and gene set enrichment analysis the data were analyzed in R using the "Limma" package to fit a linear model to the data [35]. Cut-off values were set to twofold difference in expression values with a false discovery rate of 5% (FDR < 0.05). The "ade4" package in R was used to perform a two-dimensional principal component analysis on the normalized data [36].

## Results

### Characterization of cells and validation of the use of lysates for mRNA profiling

Surface antigen profiles were obtained of the expanded cells at passage 2 (Figure S1A). Cells readily differentiated into adipogenic and osteogenic lineages verified by extensive staining of lipid droplets and calcium deposits and upregulation of *PPARG* and *OMD* (Figure S1B and C). Cells also showed differentiation into the chondrogenic lineage with upregulation of gene expression and synthesis of proteins representing key chondrogenic markers (Figure S1D and E). To evaluate if lysate of cells in alginate discs could be used instead of RNA, lysate and RNA isolated from matching samples at three timepoints grown under standard chondrogenic conditions were analyzed. Results showed highly significant correlations (p <0.0001) for all pairs with coefficients of determination (R^2^) ranging from 0.92 to 0.97 (Figure S2). This validated the use of lysate through the rest of the study.

### Principal component analysis on the full gene set

Principal component analysis (PCA) is a powerful way of reducing the dimensionality of a large data set in an unbiased way to identify clustering behaviour [37]. To see if the mRNA profiling of the full chondrogenic gene set reflected the studied conditions both regarding factors and temporal spatialization, we performed a PCA on the full dataset in all conditions at all timepoints (Figure 1B). This revealed that day 1 and day 7 samples clustered together, with larger differences observed within the day 7 cluster. We next limited the the PCA to only day 0 (untreated cells) and the full 2^5^-factorial design at day 1 (Figure 1C) or day 7 (Figure 1D) to allow for a more detailed analysis of the individual factors and combinations. At both timepoints it was readily apparent that conditions clustered according to TGFβ1 exposure, with unexposed conditions being closer to undifferentiated MSCs. On day 7 (Figure 1D) it was also evident that adding IGF1 led to only very minor differences (see for example conditions 25 and 27, 2 and 4 or 9 and 11). Notably, the 6 conditions found in the lower right quadrant of the plot all included TGFβ1 and DEX.

### Interactions between TGFβ1, DEX, BMP2, IGF1 and FGF2 evaluated by changes in selected gene subsets

**Figure 2:**
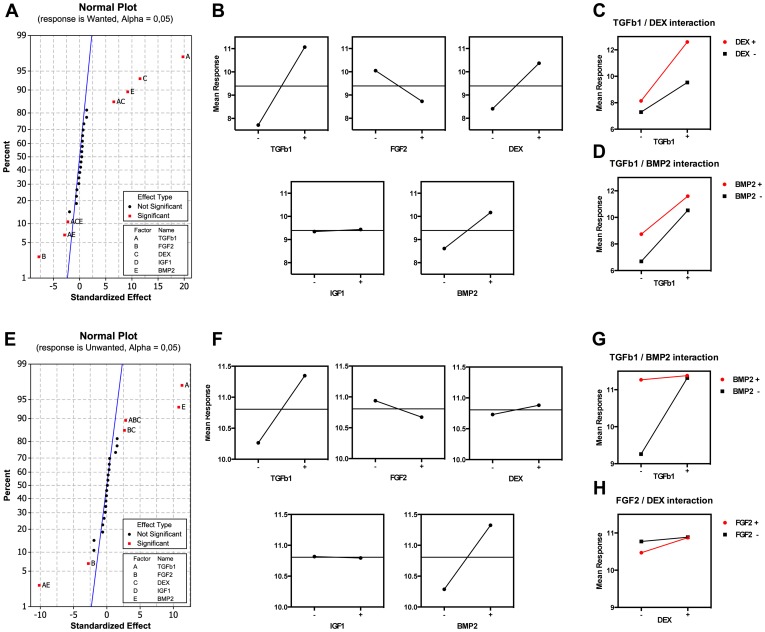
Statistical analysis of main effects and interactions at day 7. **A.** Normal plot of the standardized effects with the response set to mean expression of wanted markers. **B.** Corresponding main effects plot of all factors. **C** and **D.** Corresponding plots of significant second order interactions. **E.** Normal plot of the standardized effects with the response set to mean expression of unwanted markers. **F.** Corresponding main effects plot of all factors. **G** and **H**. Corresponding plots of significant second order interactions.

The full custom-made chondrogenic gene set comprised 364 genes including endogenous reference genes. It included both genes that are hallmarks of hyaline cartilage, but also genes that mark other differentiation processes such as adipogenesis or osteogenesis. To study the effects of the individual factors specifically on chondrogenesis we prespecified two subsets of genes: a "wanted" marker group comprised of genes coding for extracellular matrix (ECM) molecules known to be hallmarks of native hyaline cartilage [38], and the negative "unwanted" marker group comprised of genes coding for extracellular molecules distinctive for other cartilage types, but also genes coding for major transcription factors of other lineages such as adipose tissue or bone. These markers were selected based on descriptions of biological functionality in a number of selected references as described in Table 2. The mean expression of "wanted" or "unwanted" markers was used as responses when fitting a statistical regression model to the full factorial design. This allowed us to study the main effects of individual factors and significant interactions between factors on chondrogenesis. The normal plots of standardized effects using wanted and unwanted markers on day 1 (Figure S3A and D) and day 7 (Figure 2A and D) show the factors and interactions that significantly affected the wanted and unwanted responses. Focusing on day 7, TGFβ1, DEX and BMP2 affected the wanted markers significantly in the desired direction and FGF2 in the opposite direction (Figure 2B). A more complete description is seen when analyzing the significant two-way interactions of TGFβ1 with DEX and TGFβ1 with BMP2 (Figure 2C). The effect on wanted markers of TGFβ1 was dependent on the presence of DEX. TGFβ1 on its own had a much smaller effect than when added in the presence of DEX. For the interaction of TGFβ1 with BMP2 the opposite was true: adding TGFβ1 in the presence of BMP2 led to a smaller absolute increase in wanted marker expression than when TGFβ1 was added alone (Figure 2D). Only one three-way interaction, that of TGFβ1, DEX and BMP2, was found to significantly affect wanted markers. However, the standardized effect was small, and showed that both the TGFβ1/BMP2 and the TGFβ1/DEX interaction was affected by the addition of the third factor, which in both cases decreased the total effect slightly (Table S2). The effects of the differentiation factors on the expression of the unwanted marker genes were very similar to that seen for the wanted genes, with a few notable differences (Figure 2E, F, G and H). First, DEX alone did not have a significant effect (Figure 2F, Table S2). Second, both TGFβ1 and BMP2 alone increased unwanted marker expression, but in combination TGFβ1 or BMP2 did not increase unwanted expression above that seen for each of them alone (Figure 2G). FGF2 seemed to reduce the expression of wanted genes considerably, while IGF1 did not impact on this gene set at all (Figure 2B and F).

**Table 2:** Selected wanted and unwanted gene sets.

	Gene symbol	Gene name	Functional role
**WANTED**	*ACAN*	aggrecan	Major proteoglycan in hyaline cartilage
	*BGN*	biglycan	Small leucine rich proteoglycans, pericellular location and links to chondroitin sulfate in hyaline cartilage
	*COL11A1/2*	collagen, type XI, alpha1/2	Fibril forming collagen found associated with type 2 collagen in hyaline cartilage
	*COL2A1*	collagen, type II, alpha 1	The major fibril forming collagen almost exclusively found in hyaline cartilage
	*COL9A1/2/3*	collagen, type IX, alpha 1/2/3	Fibril associated collagen with interrupted triple helix found covalently linked to collagen type 2 in hyaline cartilage
	*COMP*	cartilage oligomeric matrix protein	Prominent component in the ECM of hyaline cartilage possibly stabilizing the collagen fibril assembly and network
	*DCN*	decorin	Small leucine rich proteoglycan, binds to collagen fibrils and aids in assembly
	*FMOD*	fibromodulin	Small leucine rich proteoglycan, aids in collagen assembly in cartilage in early development
	*HAPLN1*	hyaluronan and proteoglycan link protein 1	Abundant protein in cartilage, stabilizes aggregates of hyaluronan and aggrecan
	*LUM*	lumican	Leucine rich proteoglycan, aids in collagen assembly in cartilage in early development
	*MATN3*	matrilin 3	Matrix protein restricted to cartilage and binds tightly to aggrecan and/collagen fibrils
**UNWANTED**	*ALPL*	alkaline phosphatase, liver/bone/kidney	Major enzyme leading to mineralization of bone
	*COL10A1*	collagen type X, alpha 1	Network forming collagen found predominantly in hypertrophic or diseased cartilage
	*COL1A1/2*	collagen type I, alpha 1/2	Fibril forming cartilage abundant in bone ECM and virtually absent in hyaline cartilage
	*COL3A1*	collagen type III, alpha 1	Fibril forming collagen often found in mixed fibrils with collagen type 1
	*OGN*	osteoglycin	Small leucine rich proteoglycan, induces bone formation
	*PPARG*	peroxisome proliferator-activated receptor gamma	Nuclear receptor, promotes adipogenesis, stimulates lipid uptake and glucose metabolism
	*RUNX2*	runt related transcription factor 2	Transcription factor required for bone formation
	*SP7*	osterix	Transcription factor essential for osteoblastogenesis
	*SPP1*	osteopontin	Bone protein, potentiates osteoclast adhesion to mineral surfaces
	*VCAN*	versican	Proteoglycan present in fibrous and elastic cartilage, upregulated in dedifferentiating chondrocytes

Gene symbol and name of all genes comprising the wanted and unwanted marker gene sets. Based on references [28], [38], [66]–[73].

### Identifying optimal differentiation conditions from the expression of wanted and unwanted genes

**Figure 3:**
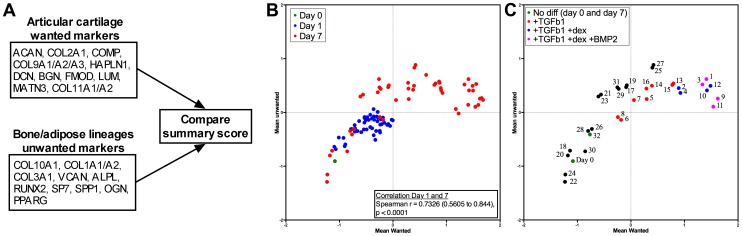
Analysis of wanted and unwanted gene expression. **A.** Gene sets used to compute mean expression of wanted and unwanted markers. **B.** Scatter-plot of the mean expression (studentized values) of wanted (x-axis) and unwanted (y-axis) markers at day 0, 1 and 7 of all conditions. **C.** Scatter-plot of the mean expression (studentized values) of wanted and unwanted markers at day 0 and 7 for condition 1–32.

As the PCA was done on the full gene set, we next wanted to explore if the changes in gene expression that segregated the different conditions reflected a desired change in terms of the expression of the wanted or unwanted gene subsets (Figure 3A). Interestingly, we found a highly significant (P < 0.0001) correlation between the ranking of conditions on day 1 and day 7 (Figure 3B), which shows that the changes in mRNA expression that arise soon after induction of differentiation can predict the direction of later changes. Corroborating the PCA, and supporting the validity of the chosen genes in the wanted and unwanted gene groups, plots of the summary score of wanted and unwanted markers show larger separation in the day 7 samples (Figure 3B). In the detailed view of day 0 and the full 2^5^-factorial design on day 7 (Figure 3C) it is clear that conditions 9 and 11 are the most favorable, with low scores for unwanted markers and the highest scores of wanted markers overall. It is also apparent from the color coding that TGFβ1 is substantially affecting expression of wanted markers in the desired direction, yet also increasing the expression of unwanted markers. DEX, on the other hand, seems to increase only expression of wanted markers if added in the presence of TGFβ1.

**Figure 4:**
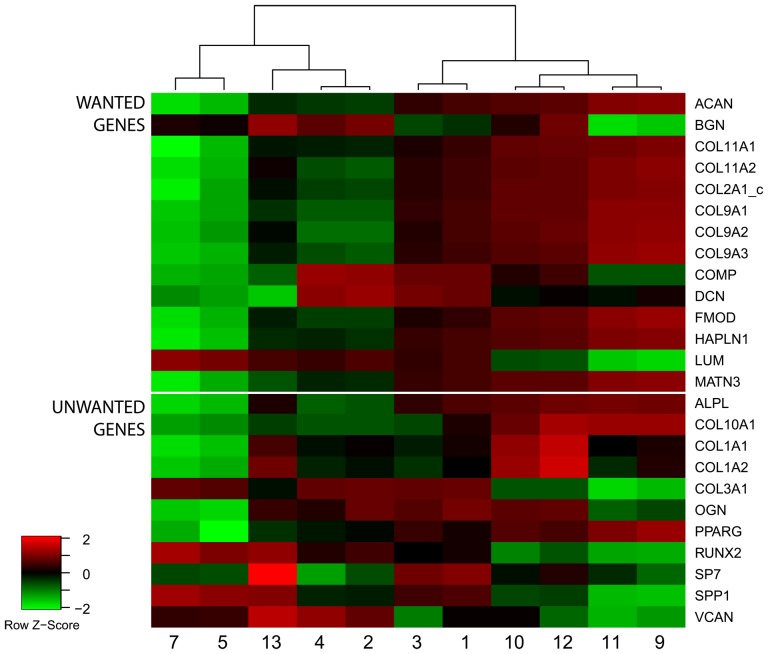
Heatmap of top ranking conditions. Heatmap of wanted and unwanted genes in all conditions significantly enriched for wanted, but not unwanted markers, color coded by the studentized score.

To support these findings we performed a gene set enrichment analysis for the wanted and unwanted gene sets. Conditions significantly enriched for the wanted gene set (FDR < 0.05), but not significantly enriched for the unwanted gene set, are highlighted in bold sorted by the FDR (Table 3). The top ranked conditions match the previous findings and a heatmap of the expression of wanted and unwanted markers of the top ten conditions (Figure 4) further visually confirms the changes with lower expressions of unwanted markers and higher expression of wanted markers in conditions 9, 11, 10 and 12. However, from the heatmap it is also apparent that individual unwanted and wanted genes such as *LUM, ALPL, COL10A1* and *PPARG* did not change in the desired direction.

**Table 3:** Gene set enrichment analysis for wanted and unwanted markers.

	False discovery rate	
Condition	Wanted	Unwanted
**11**	**<0.0001**	**0.452**
**10**	**<0.0001**	**0.273**
**9**	**<0.0001**	**0.484**
**12**	**<0.0001**	**0.296**
**1**	**<0.0001**	**0.135**
**3**	**<0.0001**	**0.138**
**13**	**<0.0001**	**0.052**
**4**	**<0.0001**	**0.080**
**2**	**<0.0001**	**0.056**
**5**	**0.003**	**0.067**
**7**	**0.035**	**0.051**
15	<0.0001	0.044
14	0.001	0.014
19	0.017	0.018
17	0.020	0.016
29	0.030	0.023
31	0.033	0.023

All conditions enriched for wanted markers are listed and ranked by the false discovery rate for wanted markers (exact FDR values are not stated for FDR <0.0001). Conditions enriched for wanted but not for unwanted markers are marked in **bold** and stated first.

The three TGFβ isoforms tested did not show substantial differences in the mean expression of wanted and unwanted markers. Addition of any TGFβ isoform increased both wanted and unwanted markers, addition of DEX to any of these increased wanted markers further and addition of BMP2 in the presence of TGFβ and DEX decreased unwanted marker expression slightly (Figure S4A). In the comparison of BMP isoforms we found that addition of any of the three BMP isoforms alone increased both wanted and unwanted marker expression, and addition of TGFβ1 further increased the expression of wanted markers (Figure S4B). Cell density upon induction of chondrogenesis affected expression both on day 1 and day 7 (Figure S4C). The expression of wanted markers on day 7 increased as the cell density was increased from 1.25 × 10^6^ to 10 × 10^6^ cells/mL. However, further increasing the cell density to 2 × 10^7^ cells/mL reduced the expression of wanted genes substantially.

### Genes uniformly affected by single factors across all conditions

**Figure 5:**
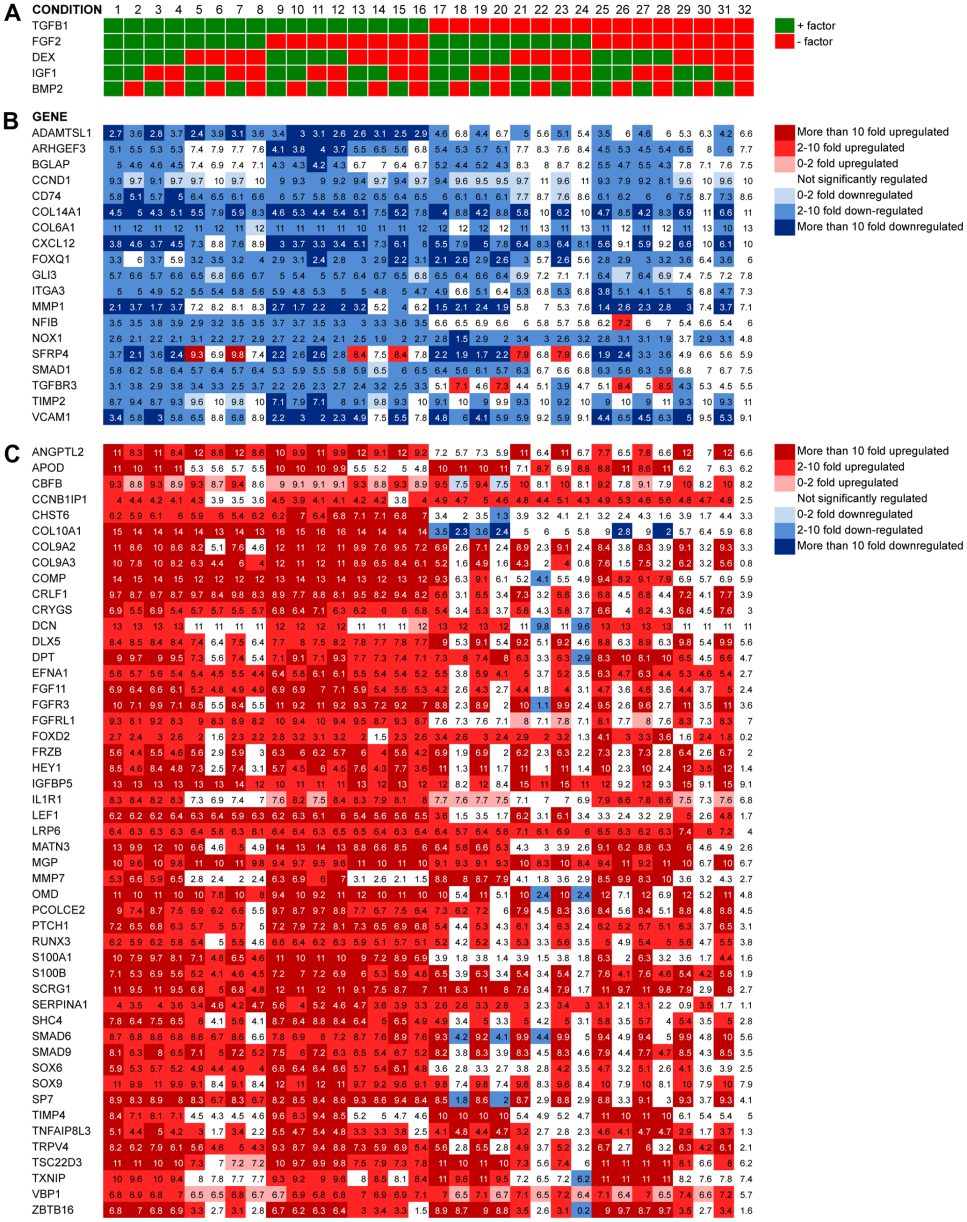
Genes uniformly affected by single factors across conditions 1–32 at day 7. **A.** Experimental setup conditions 1–32. **B.** Heatmap of genes significantly downregulated in all conditions contaning any one of the factors compared to condition 32. **C.** Heatmap of genes significantly upregulated in all conditions contaning any one of the factors compared to condition 32. Values are log2-transformed mean expressions (n  =  3).

To elucidate effects of individual factors on genes other than the selected wanted or unwanted marker genes we performed an analysis of differentially expressed genes between the bCDM and all other conditions (Figure S5). Figure 5 shows the genes that were consistently up or down regulated in all conditions with any one of the five factors. It is evident that the expression for several genes is completely dependent on the presence of a specific factor. The pro-osteoblastic gene *BGLAP*, for example, which codes for osteocalcin, was downregulated in all conditions containing DEX, but not affected in any other condition. The matrix metallopeptidase *MMP1*, which specifically degrades type I, II and III collagen, was also almost exclusively downregulated in conditions containing DEX. Genes consistently upregulated by DEX included *MMP7*, previously shown to correlate with chondrocyte maturation [39] and the tissue inhibitor of metalloproteinases 4 (*TIMP4*), known to be upregulated in response to cartilage injury and degradation [40]. Another example worth special attention is the WNT-signalling modulator *SFRP4*, known to be upregulated during adipogenesis, shown here to be consistently downregulated by DEX, and upregulated in conditions with BMP2 and no DEX, except where BMP2 was added alone or with IGF1 only [41]. *COL10A1*, a known marker of hypertrophy was consistently and exclusively upregulated in conditions with TGFβ1 [42]. Unlike practically all the other molecules upregulated by TGFβ1, for *COL10A1* the absence of TGFβ1 could not be compensated for by the addition of BMP2. *COL2A1*, which encodes for the major collagen of hyaline collage, is somewhat surprisingly not upregulated consistently by any one factor, though it is consistently upregulated in all conditions containing TGFβ1 and DEX (Figure S5). Interestingly, FGF2 could be seen to inhibit the upregulatory effect of TGFβ1 or BMP2 on *COL2A1* in all conditions where DEX was not also added. Further substantiating that DEX plays an important role in chondrogenesis is that *PRG4*, encoding the surface lubricant lubricin, was only upregulated in conditions with DEX without TGFβ-superfamily ligands [43](Figure S5).

### Genes differentially regulated between key conditions

**Figure 6:**
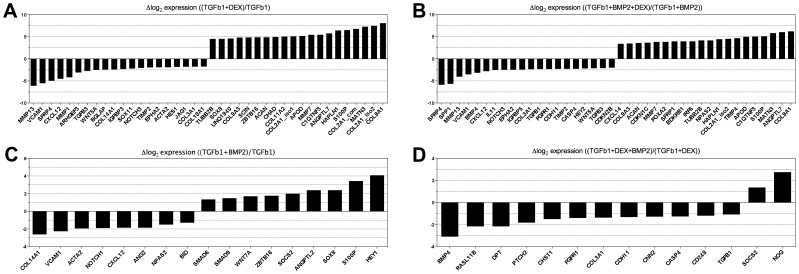
Genes significantly regulated between key conditions (day 7). **A.** Top 20 upregulated and top 20 downregulated genes when adding DEX to TGFB1. **B.** Top 20 upregulated and top 20 downregulated genes when adding DEX to TGFB1+BMP2. **C.** All regulated genes when adding BMP2 to TGFB1. **D.** All regulated genes when adding BMP2 to TGFB1+DEX. Values represent log2 to the fold change between the gene expression in the condition without and the condition with the specified factor added.

Finally, we examined genes differentially expressed between key conditions. In particular, we focused on the effect of adding DEX to either TGFβ1 or to TGFβ1+BMP2 (ie. comparing condition 12 to 16 and condition 11 to 15), or adding BMP2 to either TGFβ1 or TGFβ1+DEX (ie. comparing condition 15 to 16 and condition 11 to 12)(Figure 6A and B and Figure S6). Adding DEX to TGFβ1 changed 115 genes significantly, and adding DEX to TGFβ1 with BMP2 changed 110 genes, with an overlap of 77 genes (Figure S7 and Table S3). Several desired genes were upregulated by DEX such as *ACAN*, *COL2A1* and *SOX9* while undesired genes such as the collagen degrading metallopeptidase *MMP13* and the osteogenic transcription factor *RUNX2* were downregulated by DEX.

As expected, adding BMP2 as the second TGFβ-superfamily signalling molecule to either TGFβ1 alone or TGFβ1 with DEX changed only 17 and 14 genes respectively Surprisingly, only the upregulated gene *SOCS2* was common between these gene sets, showing that DEX importantly affects the way TGFβ1 stimulated MSCs respond to BMP2.

Given that the beneficial effect of DEX has been amply proven, and IGF1 and FGF2 have been shown to not have effects or even predominantly negative effects, the remaining question was whether BMP2 should be added to the combination of TGFβ1 and DEX. The answer to this question, at the single gene expression level, is found in Figure 6D. The most highly upregulated gene, *NOG*, encodes a polypeptide noggin that binds and inactivates BMPs belonging to the TGFβ-superfamily, particularly BMP4 [44], [45]. Also the addition of BMP2 on a background of TGFβ1 + DEX leads to downregulation of *BMP4* at the mRNA level, which could be a direct effect of BMP2 or perhaps an effect by noggin also on BMP4 mRNA expression. *NOTCH1*, which has been shown to be required in early chondrogenesis but must be turned off for full chondrogenesis to occur [30], [46], was also downregulated. In addition, several other downregulated genes such as *DPT*, *FGFR1* and *TGFB1* are likely to have pro-chondrogenic effects [15], [47]. One positive effect of BMP2 was the downregulation of *COL3A1*, a collagen frequently coexpressed with type I collagen in connective tissues [48].

In total, these data indicate that addition of BMP2 to a chondrogenic cocktail already consisting of TGFβ1 and DEX will not improve cartilage formation, at least judging by the expression of genes of relevance for chondrogenesis.

## Discussion

Directed differentiation of stem cells into chondrocytes in vitro has been shown to require both three-dimensional culture and environmental ques in the form of growth factors [7], [16], [17], [49]. These cocktails of growth factors have largely been studied by manipulating one factor at a time, which is laborous and time consuming. We show here that high-throughput gene profiling makes it feasible to perform larger scale experiments with statistical design of experiments, allowing for sound conclusions on the involvement of many simultaneously investigated factors [24], [50], [51].

In the present study we used this approach to dissect the expression of a chondrogenesis relevant gene set during in vitro chondrogenesis of MSCs subjected to 48 different conditions of growth factors and cell densities. We found that only three of the factors (TGFβ1, DEX, and BMP2) directly increased the expression of chondrogenic markers significantly. Adding FGF2 or IGF1, either alone or in combination with other factors, had either no effect or predominantly negative effects on the expression of chondrogenic genes.

TGFβ1 is the most extensively used factor for inducing chondrogenesis in directed differentiation of MSCs [10]. The present data show that the related factors TGFβ2 and 3, but no single other factor studied here, can replace its positive effects on chondrogenic differentiation of human MSCs. Next, we found that adding DEX to TGFβ1 changed more that 100 of the investigated genes significantly, with the vast majority of changes being favourable for chondrogenesis. This is in line with the use of DEX in most of the published literature [7], [10], [14], although a recent publication actually concluded that DEX should be omitted [52]. The present study extends current knowledge by describing which of a large set of relevant genes are changed by each of these factors, and then by two together.

BMPs can, like the TGFβ isoforms, promote MSC differentiation into chondrocytes [53], [54]. However, BMPs and TGFβ have also been described to exhibit antagonistic activities in many tissues [55]. We found that both factors increased wanted chondrogenic markers on their own. Interestingly, the two combinations identified with the best ratio of wanted to unwanted genes and highest mean expression of wanted markers contained both TGFβ1 and BMP2. However the effect of adding either factor together with the other was marginal and not synergistical on either wanted or unwanted markers. This is most likely explained by the molecular mechanism for the actions of these factors. Both BMP2 and TGFβ1 are ligands of the transformings growth factor β superfamily and act by binding to specific type II receptors, which recruits the corresponding type I receptor, ultimately leading to phosporylation of receptor-SMADs. Even though BMP2 works mainly through SMAD1, 5 and 8 and TGFβ1 through SMAD2 and 3, there are known interactions between the two systems such as the competitive occupation of the common downstream effector SMAD4 [55]. Also it seems that adding BMP2 to TGFβ1 and DEX does not lead to a significant upregulation of any genes positively related to chondrogenesis, but rather to an endogenous modulation of BMP4. Taken together, there may be more reasons to exclude BMP2 than to include it in a chondrogenic differentiation cocktail, although it could have a role in a system with sequential cocktails for different parts of chondrogenesis, as illustrated by the effect on *PRG4*. This is in contrast to findings in the literature that BMP2, -4 or -6 are beneficial to in vitro chondrogenesis in pellet culture [16], [17]. This finding may be explained by the difference between scaffold based culture systems such as alginate and pellet or micromass culture systems. However, the present analysis also included more genes, and thus was more detailed than the initial experiments leading to the inclusion of BMPs in the differentiation cocktails used in many, but not all, labs studying in vitro chondrogenesis.

The FGF2 treatment did not lead to an increase in the chondrogenic gene expression. On the contrary, a significant reduction was seen in the general gene expression of both wanted and unwanted genes when FGF2 was added to the basic differentiation cocktail. In line with our finding, it has been published that FGF2 may abolish chondrogenesis when combined with TGFβ1 and BMP-6 [56]. On the other hand, FGF2 has recently been described to enhance the potential of MSCs for use in tissue engineering of cartilage when used as a mitogen in the expansion phase prior to the differentiation [18], [57].

We found that IGF1 did not change the general expression of either wanted or unwanted genes significantly, which is contrary to some previous publications [21]. IGF1 has been shown to be expressed in articular cartilage and regulate proteoglycan metabolism [58] and it has a distinct expression profile during embryogenic chondrogenesis [59]. However our finding is in line with other publications failing to find effects of using IGF1 to induce chondrogenesis [60], [61].

There are limitations to our approach. We only considered gene expression on the level of transcribed mRNA, which does not necessarily correlate with protein synthesis [62]. We also utilized a two-level factorial design with either absence or presence of the investigated growth factors, which did not allow an assesment of the role that different factor concentrations might play. Considering these limitations, we propose that the method presented here could be adapted to screen large numbers of molecules that could enhance chondrogenesis. We also believe that the method described could be valuably expanded to testing several concentrations of factors, which would also allow a statistical analysis with response optimization to be performed [24]. This could be a particularly valuable way forward, as the concentrations of growth factors used in the literature rarely are based on complete dose-response experiments, and they are frequently used without a clear relation to physiologic concentrations. Recently, screening experiments on large libraries of novel drug-like molecules have also been performed looking for compounds that increase chondrogenesis based on simpler initial assays [63]. The approach used in the present paper could easily be adapted for such a purpose allowing for the added value of a more stringent selection of new molecules enhancing wanted but not unwanted genes. Further supporting the feasibility of our approach in larger screening experiments is our finding that gene profiling can be performed directly on lysates without any loss in assay quality. Also the finding that changes in gene expression seen just one day after induction predicts later changes, potentially allows for a simpler design with just one time point, perhaps earlier than the one week time point chosen in the present study. Combined, the implications of these findings could decrease both cost and workload considerably in future experiments. Finally, larger screening experiments could be efficiently performed in a fractionalized factorial design allowing for sound conclusions without increasing the number of experiments [23], [64]. However, to test temporal spatialization of chondrogenic factors to more exactly mirror the conditions known from embryogenesis of cartilage, the best approach might be to combine the mRNA profiling assay described here with staining and imaging assays used to describe the composition and structure of the ECM [65].

## Conclusion

In this study we have shown that high-throughput mRNA profiling can be efficiently performed on lysates of MSCs during in vitro chondrogenesis in alginate. A thorough analysis revealed that the cocktail of growth factors leading to the most efficient upregulation of wanted chondrogenic markers was a combination of TGFβ and DEX. Adding BMP2 lead to a slightly higher mean expression of wanted markers but did not significantly upregulate key positive genes and led to a downregulation of endogenous BMP4 and TGFβ1 expression, and may therefore be expendable. DEX, on the other hand, worked synergistically with TGFB1 in increasing wanted marker expression and was also directly downregulating expression of the unwanted marker BGLAP. All factors beneficial to the expression of wanted hyaline cartilage markers also introduced an induction of unwanted markers, with the exception of DEX alone. Upregulation of *COL10A1* was seen in all conditions containing TGFβ1 indicating that perfect differentiation to hyaline cartilage is not achievable with the current differentiation protocols.

## Supporting Information

Appendix S1 **Supplementary Methods and materials.**
(DOCX)

Appendix S2 **Nanostring Gene Set.**
(XLS)

Figure S1 **Quality control of MSCs.** **A**. Light microscopy picture of MSCs and surface marker profiles of MSCs as measured by flow cytometry before differentiation (passage 2 or 3). **B**. Light microscopy pictures of stained control (CTRL) and differentiated (DIFF) MSCs on day 21. Upper panel; adipogenic differentiated cells stained with Oil Red-O: Lower panel: osteogenic differentiation of MSCs stained with Alizarin Red. **C**. Fold change of the expression of PPARG in adipogenic differentiated cells and OMD in osteogenic differentiated relative to control treated cells measured by qPCR (mean±SE, n = 3). **D**. Chondrogenically differentiated MSCs in alginate with TGFB1, DEX and BMP2 as shown by mRNA expression changes in key chondrogenic markers (n = 3, mean±SE) with corresponding protein synthesis (**E**.) in a representative sample on day 21. Nuclei counterstained with DAPI (blue). Scale bar  =  50 µM.
(TIF)

Figure S2 **Correlation between gene expression analyzed in lysates and in isolated total RNA from corresponding samples.** Correlation plots of the expression of genes analyzed in lysates and in total RNA in two donors at day 1, 7 and 14 with coefficients of determination (Pearson's correlation).
(TIFF)

Figure S3 **Statistical analysis of main effects and interactions at day 1.** **A**. Normal plot of the standardized effects with response set to mean expression of wanted markers. **B**. Corresponding main effects plot of all factors. **C**. Corresponding plots of significant second order interactions. **D**. Normal plot of the standardized effects with response set to mean expression of unwanted markers. **E**. Corresponding main effects plot of all factors. **F**. Corresponding plots of significant second order interactions.
(TIF)

Figure S4 **Analysis of wanted and unwanted gene expression.** **A**. Scatter-plot of the mean expression (studentized values) of wanted and unwanted markers at day 0 and 7 for conditions containing isoforms of TGFB (n  =  2). **B**. Scatter-plot of the mean expression (studentized values) of wanted and unwanted markers at day 0 and 7 for conditions with isoforms of BMP. (n  =  2) **B**. Scatter-plot of the mean expression (studentized values) of wanted and unwanted markers at day 0 and 7 for condition with varying cell seeding density. (n  =  2)
(TIFF)

Figure S5 **Genes significantly up- or downregulated.** Heatmap of all genes significantly changed compared to condition 32 at day 7. Values are log2-transformed mean expressions (n  =  3)
(PDF)

Figure S6 **Genes significantly regulated between key conditions (day 7).** **A.** All significantly regulated genes (>2-fold) when adding DEX to TGFB1. **B.** All significantly regulated genes (>2-fold) when adding DEX to TGFB1+BMP2. Values represent log2 to the fold change between the gene expression in the condition without and the condition with the specified factor added.
(TIFF)

Figure S7 **Overlapping regulated genes when adding DEX to TGF or to TGFB1 with BMP2.** Venn diagram illustrating the overlap of genes significantly up- or downregulated corresponding to Figure S6.
(TIF)

Table S1 **Primary and secondary antibodies used in immunohistochemistry.**
(DOC)

Table S2 **Statistical response analysis of main effects and second and third order interactions.**
(DOCX)

Table S3 **Significantly regulated genes uniformly up- or downregulated by DEX.**
(DOCX)
