# Supplementary material for: Analysis of the Effects of Five Factors Relevant to In Vitro Chondrogenesis of Human Mesenchymal Stem Cells Using Factorial Design and High Throughput mRNA-Profiling
Source: PLoS One. 2014 May 9;9(5):e96615. doi: 10.1371/journal.pone.0096615 (PMC4015996; doi:10.1371/journal.pone.0096615)
Supplement: Appendix S1 — Supplementary Methods and materials. (DOCX) [file pone.0096615.s001.docx]

**Supporting information Appendix S1: Methods and materials**

## Cell harvest and culture

Bone marrow aspirates were obtained in heparin-coated syringes and immediately diluted 1:4 with DMEM-F12 (Gibco) and centrifuged on a density gradient (Lymphoprep, Axis Shield, Oslo, Norway). The mononuclear layer was washed twice and seeded into 175 cm^2^ culture flask (Nunc, Roskilde, Denmark). Cells were allowed to adhere for 72 hours before the first medium change. Medium was then changed every 3-4 days. Cells were passaged when approximately 75% confluent and reseeded at a density of 5000 cells/cm^2^.

## Medium and supplements

Human platelet lysate (hPL) was prepared as published previously[[1](#_ENREF_1),[2](#_ENREF_2)]. Briefly, 1 unit of pooled platelets (4 donors) in platelet additive solution was spun at 1700 g at room temperature. The resulting pellet was resuspended in 10 mL Octaplas AB (Octapharma AS, Jessheim, Norway) and frozen at -20°C. After thawing, platelets from 19 units were pooled and adjusted to a final volume of 4,8 L with Octaplas AB, subjected to a second freeze-thaw-cycle, centrifuged at 4000 g at 4°C for 15 minutes to remove platelet fragments and frozen in aliquotes.

## Validation of cells as MSC

Characterization was performed as previously described[[3](#_ENREF_3)]. Fluorochrome conjugated antibodies used for surface marker characterization were CD19-APC, CD105-APC, HLA-DR-APC, CD14-FITC (all Diatec, Oslo, Norway), CD34-FITC, CD44-FITC, CD45-PE, CD73-PE (all BD-Biosciences, San Jose, CA) and CD90-PE (Serotec, Oxford, UK) and appropriate IgG control antibodies. Multipotency was validated by osteogenic and adipogenic differentiation[[4](#_ENREF_4)]. For osteogenic differentiation cells were seeded at 3000 cells/cm^2^ and differentiated with DMEM-F12 containing 10 % hPL, 10 mM β-glycerophosphate, 100 nM DEX and 0.05 mM ascorbic acid-2-phosphate. Medium was changed twice a week. For adipogenic differentiation cells were seeded at 50,000 cells/cm^2^ and differentiated with DMEM-F12 containing 10 μg/mL insulin (Novo Nordisk, Bagsvaerd, Denmark), 0.5 μM 1-methyl-3-isobutylxanthine, 1 μM DEX and 100 μm indomethacin (Dumex-Alpharma, Copenhagen, Denmark). Medium was changed twice a week. For both differentiation assays, after 3 weeks, the cells were washed with phosphate-buffered saline (PBS), fixed for 1 h with 4% paraformaldehyde, and rinsed with PBS. Mineralisation of osteogenic cultures was confirmed by staining with 40 mM Alizarin Red (ph 4.2) for 5 min. Lipid droplets in adipogenic cultures were confirmed by staining with Oil Red O for 10 min. Appropiate control cultures receiving standard growth medium were also stained and evaluated.

## 3D cell culture

Briefly, cells were resuspended into a 1% Pronova-LVG solution. 62,5 μL were transferred to 16-well chamberslides (Labtek, Nunc, Denmark) and mixed with an equal volume of 1% Pronova-calcium-alginate containing slow releasing calcium particles, giving a final cell density of 1x10^7^ cells/mL. After stabilisation for 15 minutes, discs were transferred to 24-well culture plates (Nunc) where gelling was consolidated by washing with 50 mM SrCl_2_, followed by three times of washing with regular DMEM before addition of bCDM with or without additional growth factors according to the experimental factorial design. Medium was changed three times per week.

## Immunohistochemical stainings

Primary and secondary antibodies and all final concentrations are specified in Table S1. Formalin-fixed, paraffin-embedded samples of cells in alginate were sectioned and deparaffinized using standard laboratory procedures and postfixed for 10 minutes in 4% paraformaldehyde in PBS. Tissue sections were boiled for 20 minutes in 0.05% citraconic anhydride in ddH2O (pH 7.4), incubated with primary antibodies diluted in PBS/1.25% bovine serum albumin with 0.1% saponin for permeabilization overnight at 4°C, followed by secondary reagents for 1.5 hour at room temperature. Stained sections were mounted using ProLong Gold antifading reagent with DAPI (Invitrogen, Carlsbad, CA). Microscopy was performed with a Nikon Eclipse E-600 fluorescence microscope equipped with Nikon Plan-Fluor objective lenses and a Color View III digital camera controlled by Cell-B software (Olympus, Center Valley, PA).

**REFERENCES**

1. Schallmoser K, Strunk D (2009) Preparation of pooled human platelet lysate (pHPL) as an efficient supplement for animal serum-free human stem cell cultures. J Vis Exp.

2. Karlsen TA, Jakobsen RB, Mikkelsen TS, Brinchmann JE (2013) microRNA-140 targets RALA and regulates chondrogenic differentiation of human mesenchymal stem cells by translational enhancement of SOX9 and ACAN. Stem Cells Dev.

3. Herlofsen SR, Kuchler AM, Melvik JE, Brinchmann JE (2011) Chondrogenic differentiation of human bone marrow-derived mesenchymal stem cells in self-gelling alginate discs reveals novel chondrogenic signature gene clusters. Tissue Eng Part A 17: 1003-1013.

4. Shahdadfar A, Fronsdal K, Haug T, Reinholt FP, Brinchmann JE (2005) In vitro expansion of human mesenchymal stem cells: choice of serum is a determinant of cell proliferation, differentiation, gene expression, and transcriptome stability. Stem Cells 23: 1357-1366.
