# Supplementary figures and images for: Analysis of the Effects of Five Factors Relevant to In Vitro Chondrogenesis of Human Mesenchymal Stem Cells Using Factorial Design and High Throughput mRNA-Profiling
Source: PLoS One. 2014 May 9;9(5):e96615. doi: 10.1371/journal.pone.0096615 (PMC4015996; doi:10.1371/journal.pone.0096615)

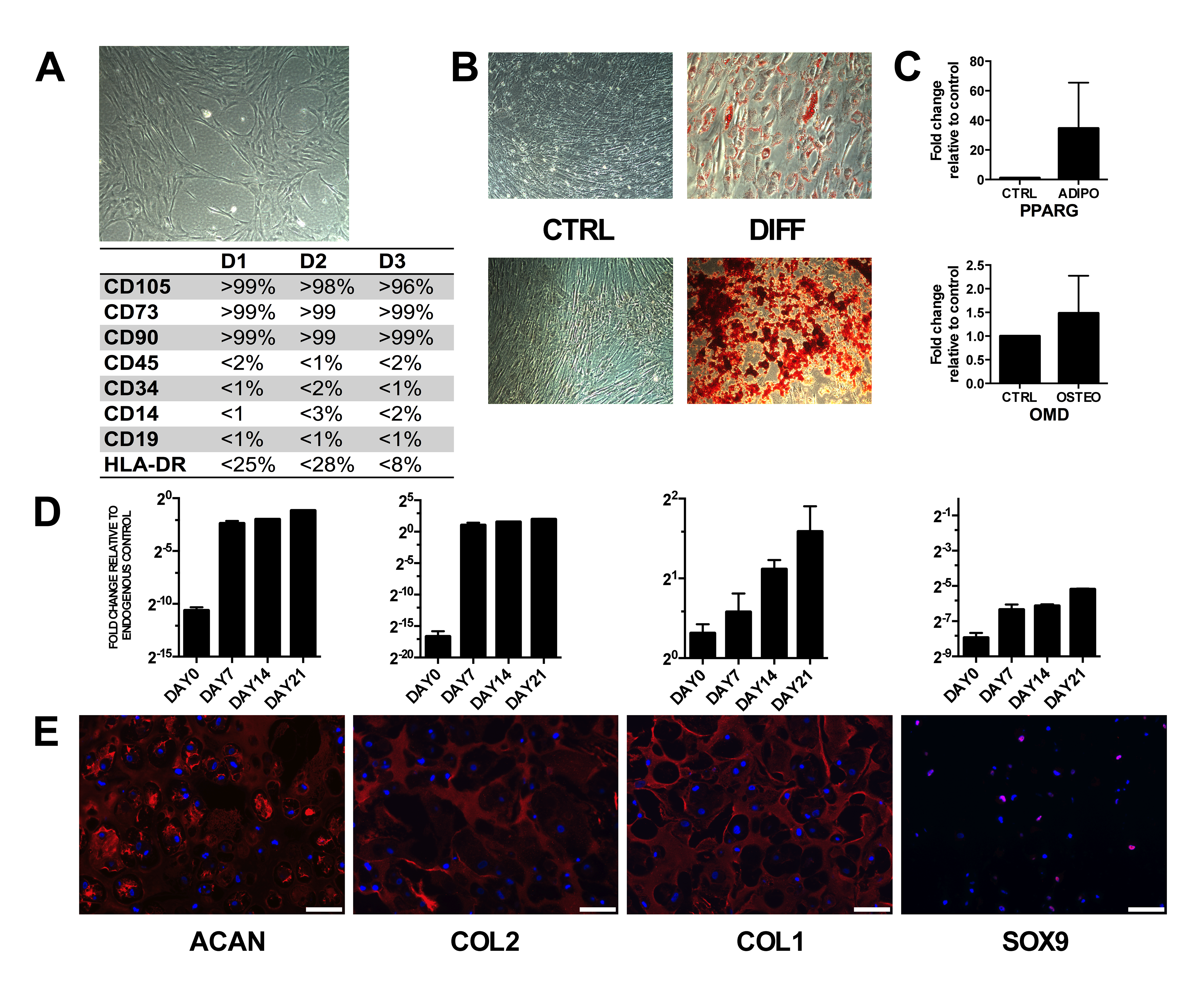

Supplement: Figure S1 — Quality control of MSCs. A. Light microscopy picture of MSCs and surface marker profiles of MSCs as measured by flow cytometry before differentiation (passage 2 or 3). B. Light microscopy pictures of stained control (CTRL) and differentiated (DIFF) MSCs on day 21. Upper panel; adipogenic differentiated cells stained with Oil Red-O: Lower panel: osteogenic differentiation of MSCs stained with Alizarin Red. C. Fold change of the expression of PPARG in adipogenic differentiated cells and OMD in osteogenic differentiated relative to control treated cells measured by qPCR (mean±SE, n = 3). D. Chondrogenically differentiated MSCs in alginate with TGFB1, DEX and BMP2 as shown by mRNA expression changes in key chondrogenic markers (n = 3, mean±SE) with corresponding protein synthesis (E.) in a representative sample on day 21. Nuclei counterstained with DAPI (blue). Scale bar = 50 µM. (TIF) [file pone.0096615.s003.tif]

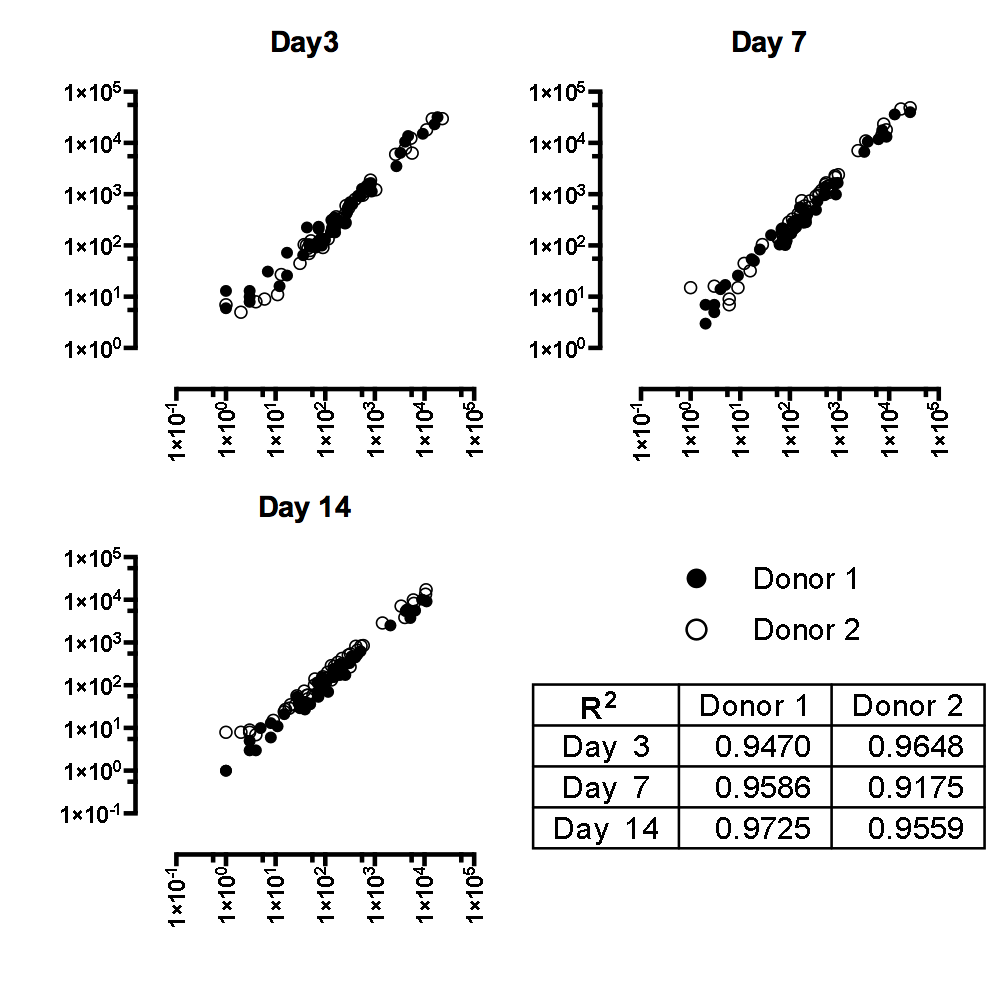

Supplement: Figure S2 — Correlation between gene expression analyzed in lysates and in isolated total RNA from corresponding samples. Correlation plots of the expression of genes analyzed in lysates and in total RNA in two donors at day 1, 7 and 14 with coefficients of determination (Pearson's correlation). (TIFF) [file pone.0096615.s004.tif]

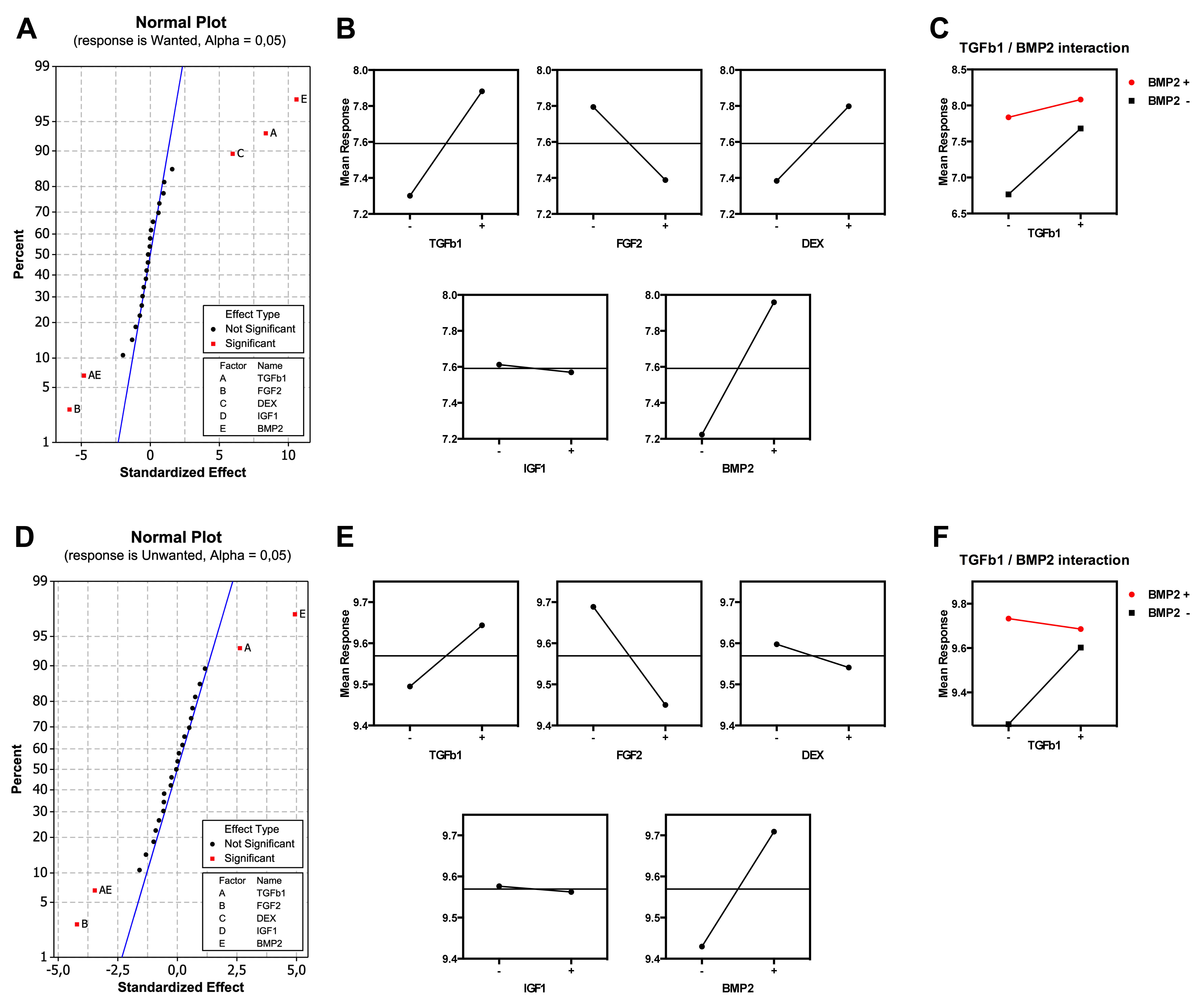

Supplement: Figure S3 — Statistical analysis of main effects and interactions at day 1. A. Normal plot of the standardized effects with response set to mean expression of wanted markers. B. Corresponding main effects plot of all factors. C. Corresponding plots of significant second order interactions. D. Normal plot of the standardized effects with response set to mean expression of unwanted markers. E. Corresponding main effects plot of all factors. F. Corresponding plots of significant second order interactions. (TIF) [file pone.0096615.s005.tif]

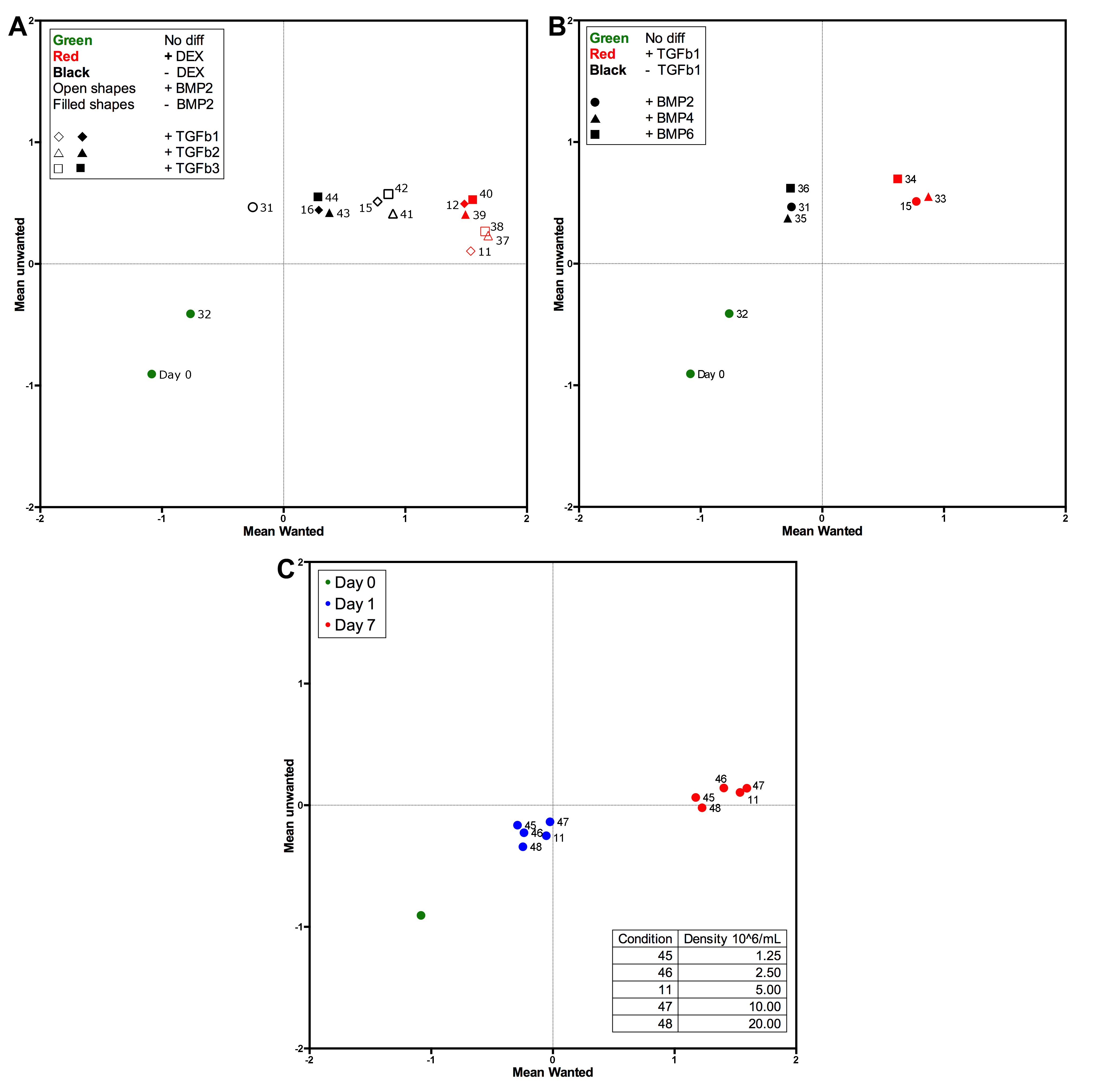

Supplement: Figure S4 — Analysis of wanted and unwanted gene expression. A. Scatter-plot of the mean expression (studentized values) of wanted and unwanted markers at day 0 and 7 for conditions containing isoforms of TGFB (n = 2). B. Scatter-plot of the mean expression (studentized values) of wanted and unwanted markers at day 0 and 7 for conditions with isoforms of BMP. (n = 2) B. Scatter-plot of the mean expression (studentized values) of wanted and unwanted markers at day 0 and 7 for condition with varying cell seeding density. (n = 2) (TIFF) [file pone.0096615.s006.tif]

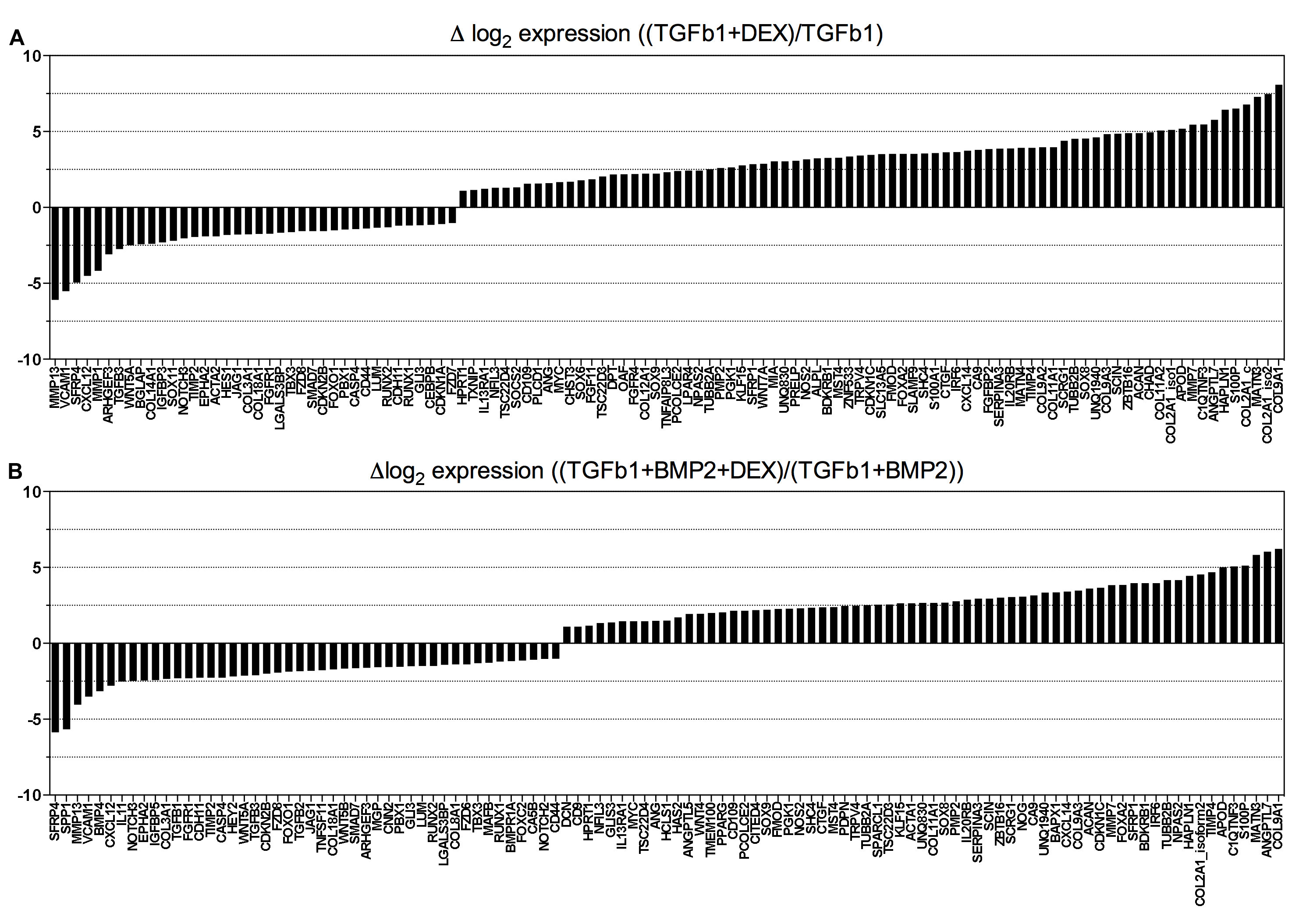

Supplement: Figure S6 — Genes significantly regulated between key conditions (day 7). A. All significantly regulated genes (>2-fold) when adding DEX to TGFB1. B. All significantly regulated genes (>2-fold) when adding DEX to TGFB1+BMP2. Values represent log2 to the fold change between the gene expression in the condition without and the condition with the specified factor added. (TIFF) [file pone.0096615.s008.tif]

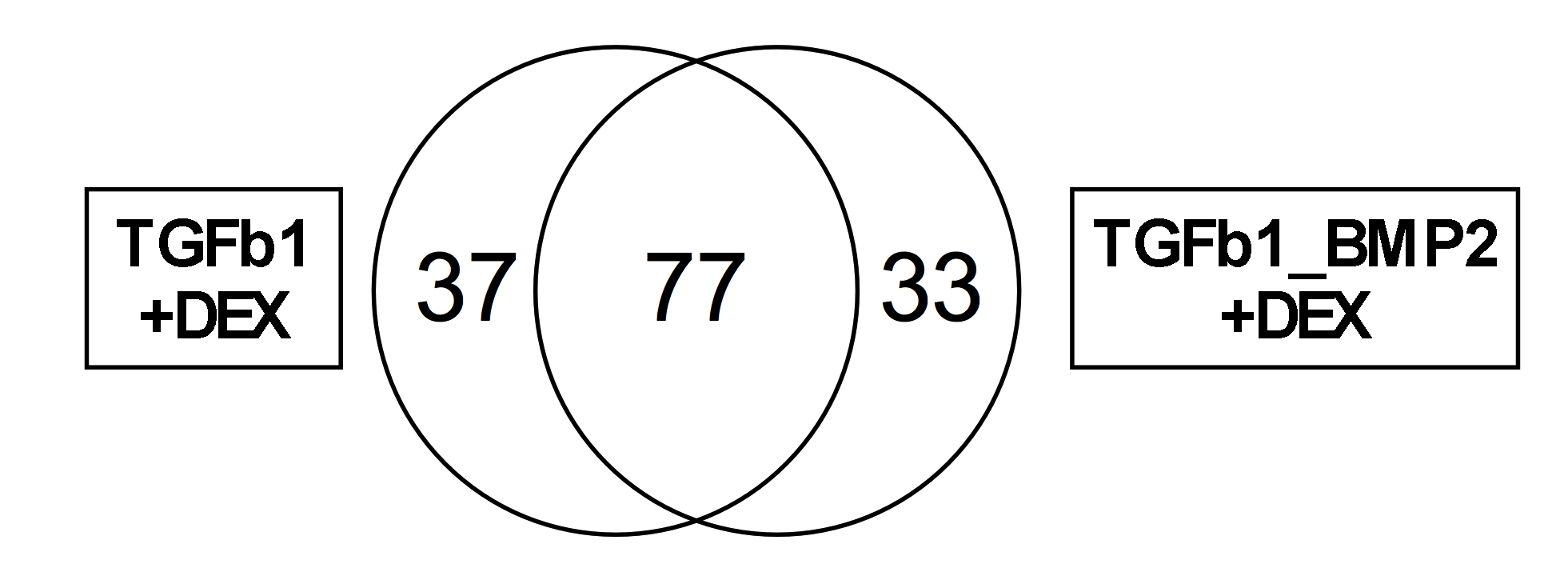

Supplement: Figure S7 — Overlapping regulated genes when adding DEX to TGF or to TGFB1 with BMP2. Venn diagram illustrating the overlap of genes significantly up- or downregulated corresponding to Figure S6. (TIF) [file pone.0096615.s009.tif]
