# Supplementary material for: Analysis of the Effects of Five Factors Relevant to In Vitro Chondrogenesis of Human Mesenchymal Stem Cells Using Factorial Design and High Throughput mRNA-Profiling
Source: PLoS One. 2014 May 9;9(5):e96615. doi: 10.1371/journal.pone.0096615 (PMC4015996; doi:10.1371/journal.pone.0096615)
Supplement: Figure S5 — Genes significantly up- or downregulated. Heatmap of all genes significantly changed compared to condition 32 at day 7. Values are log2-transformed mean expressions (n = 3) (PDF) [file pone.0096615.s007.pdf]

CONDITION	1	2	3	4	5	6	7	8	9	10	11	12	13	14	15	16	17	18	19	20	21	22	23	24	25	26	27	28	29	30	31	32
TGFB1	+	+	+	+	+	+	+	+	+	+	+	+	+	+	+	+	-	-	-	-	-	-	-	-	-	-	-	-	-	-	-	-
FGF2	+	+	+	+	+	+	+	+	-	-	-	-	-	-	-	-	+	+	+	+	+	+	+	+	-	-	-	-	-	-	-	-
DEX	+	+	+	+	-	-	-	-	+	+	+	+	-	-	-	-	+	+	+	+	-	-	-	-	+	+	+	+	-	-	-	-
IGF1	+	+	-	-	+	+	-	-	+	+	-	-	+	+	-	-	+	+	-	-	+	+	-	-	+	+	-	-	+	+	-	-
BMP2	+	-	+	-	+	-	+	-	+	-	+	-	+	-	+	-	+	-	+	-	+	-	+	-	+	-	+	-	+	-	+	-

+ factor
- factor

GENE	1	2	3	4	5	6	7	8	9	10	11	12	13	14	15	16	17	18	19	20	21	22	23	24	25	26	27	28	29	30	31	32
ACAN	12.9	10.4	12.6	10.5	8.1	5.8	7.4	5.7	14.2	13.1	14.1	13.3	10.8	8.8	10.5	8.4	10.7	4.1	10.4	3.9	8.3	5.6	8.3	5.1	11.8	5.0	11.8	4.5	10.5	6.0	10.3	6.4
ACTA1	1.9	2.3	2.5	1.7	2.5	2.2	3.3	1.8	3.7	3.0	3.4	3.0	2.5	2.5	0.7	2.6	0.8	1.9	1.5	0.8	2.2	1.9	2.4	1.7	1.9	2.2	1.9	1.6	2.6	2.0	2.0	2.3
ACTA2	7.1	9.1	7.3	9.1	7.4	8.8	7.2	9.4	6.6	7.1	6.2	6.9	7.1	8.9	6.8	8.8	9.0	8.7	9.0	8.7	7.7	8.7	7.7	8.6	8.5	9.0	8.4	9.0	8.4	9.7	8.0	10.0
ADAMTS1	5.6	4.8	5.2	5.0	5.3	6.4	5.7	6.1	4.9	4.7	4.9	4.8	5.9	4.9	5.4	5.2	7.7	8.2	7.8	8.3	7.8	6.8	7.8	6.8	8.1	9.2	8.1	9.2	8.2	6.6	8.4	6.0
ADAMTS5	4.2	4.3	4.3	4.2	5.3	6.3	5.5	6.2	3.5	3.6	3.3	3.8	4.8	4.7	4.6	5.0	4.4	4.7	4.4	4.3	4.8	4.4	5.4	4.3	4.8	4.9	4.6	5.6	5.0	4.5	4.9	3.9
ADAMTS7	6.1	6.1	6.2	6.1	6.3	6.3	6.6	6.2	5.4	6.0	5.4	6.2	6.2	6.3	6.1	6.5	7.5	5.7	7.5	5.8	7.1	6.0	7.1	6.3	7.8	6.6	7.9	6.5	7.6	7.1	7.5	7.2
ADAMTSL1	2.7	3.6	2.8	3.7	2.4	3.9	3.1	3.6	3.4	3.0	3.1	2.6	2.6	3.1	2.5	2.9	4.6	6.8	4.4	6.7	5.0	5.6	5.1	5.4	3.5	6.0	4.6	6.0	5.3	6.3	4.2	6.6
ADAMTSL4	4.1	4.4	4.5	4.5	3.9	3.7	4.3	3.7	4.5	3.8	4.2	3.9	4.0	4.2	3.7	3.9	5.3	7.5	5.2	7.4	4.3	4.5	4.4	4.1	5.6	7.4	5.9	7.4	5.0	5.4	4.8	3.3
AGT	3.6	3.4	3.3	3.2	3.2	4.0	3.7	4.3	3.7	4.0	3.4	4.4	4.6	4.5	4.3	4.2	4.4	2.0	4.4	2.5	3.5	4.3	3.6	3.6	5.1	4.5	4.7	3.7	3.1	3.2	3.3	5.0
AGTR1	4.1	3.9	3.9	3.8	4.1	3.8	4.6	4.1	2.9	3.2	3.3	2.5	3.6	3.9	2.6	3.9	9.2	7.3	9.3	7.4	7.1	4.8	7.0	4.8	7.8	8.4	7.7	8.3	6.2	6.0	6.3	5.5
AGTR2	3.5	3.2	3.4	3.3	3.4	3.4	3.9	3.4	3.3	2.6	2.5	3.1	3.4	3.8	3.6	3.5	2.9	2.3	3.9	1.5	3.9	2.0	3.3	1.7	2.7	2.2	3.6	2.9	3.4	3.3	2.8	2.3
AGTRAP/C11orf2	6.9	7.2	6.8	7.2	6.4	6.7	6.5	6.6	6.6	6.7	6.7	6.9	6.7	6.7	6.8	6.7	6.5	6.1	6.3	6.1	6.5	6.1	6.4	6.2	6.3	6.3	6.4	6.3	6.7	6.6	6.2	6.9
ALPL	10.5	8.0	10.0	7.7	6.2	2.8	5.8	3.4	11.2	10.8	11.2	11.1	9.8	7.9	9.6	7.8	10.4	6.9	10.5	6.7	8.2	1.8	8.0	2.5	12.0	5.4	12.2	5.8	9.7	5.2	9.6	5.1
ANG	5.2	5.0	5.0	4.5	4.0	3.7	3.8	3.4	5.9	5.0	5.6	5.3	4.0	3.8	4.1	3.7	4.8	5.4	4.9	5.4	4.2	3.6	4.4	3.7	5.0	6.2	5.1	6.1	4.4	3.7	4.4	4.3
ANG2	3.6	3.3	3.3	2.9	2.9	3.1	3.4	3.0	2.3	2.8	2.3	2.7	3.2	3.9	1.8	3.7	5.6	3.3	5.6	3.4	3.5	2.2	3.6	2.9	5.2	4.8	5.3	4.9	2.7	2.2	4.2	1.1
ANGPTL2	11.3	8.3	11.2	8.4	11.7	8.8	11.8	8.6	10.2	9.9	10.5	9.9	11.7	9.1	11.6	9.2	7.2	5.7	7.3	5.9	11.4	6.4	11.2	6.7	7.7	6.5	7.8	6.6	11.7	7.0	11.8	6.6
ANGPTL4	6.1	6.1	6.0	5.7	6.5	6.1	6.5	5.7	6.4	4.6	5.3	4.8	5.1	5.5	5.9	5.7	6.3	6.7	6.5	6.6	5.9	5.6	6.0	5.1	6.1	6.6	6.4	6.8	7.3	6.9	6.7	7.4
ANGPTL5	4.4	3.2	4.2	3.4	2.9	3.1	3.6	2.7	5.4	4.8	5.4	4.7	3.7	3.1	3.4	3.3	2.9	3.7	3.3	3.6	2.8	2.6	3.5	2.2	4.2	4.9	4.3	4.4	3.0	1.5	2.6	2.0
ANGPTL7	8.5	7.0	8.2	7.3	4.1	3.0	2.8	2.4	10.7	9.8	10.6	9.9	6.0	4.7	4.5	4.2	2.8	2.3	2.7	2.5	3.2	3.0	2.8	2.5	4.0	3.7	4.2	3.5	3.0	3.7	3.4	4.2
APOD	10.6	10.3	10.8	10.6	5.3	5.6	5.7	5.5	10.3	10.1	10.0	9.9	5.5	5.2	5.0	4.8	10.1	11.1	10.1	11.2	7.1	8.7	6.9	8.8	8.8	10.9	8.6	10.9	6.2	7.0	6.3	6.2
APOE	3.4	2.8	2.7	3.1	3.1	3.1	3.5	3.2	2.0	2.6	2.1	2.5	2.7	1.6	3.3	2.7	5.9	8.7	5.8	8.7	5.5	4.1	5.3	4.3	6.4	9.7	6.2	9.7	5.0	4.7	5.5	5.0
ARHGEF19	7.5	7.2	7.2	7.0	6.5	5.3	6.5	5.5	6.1	6.4	6.0	6.5	6.7	6.4	6.4	6.3	7.0	5.1	6.9	5.3	6.2	3.6	6.0	2.7	7.6	6.8	7.2	6.7	6.6	3.3	6.8	4.5
ARHGEF3	5.1	5.5	5.3	5.3	7.4	7.9	7.7	7.6	4.1	3.8	4.0	3.7	5.5	6.5	5.6	6.8	5.4	5.3	5.7	5.1	7.7	8.3	7.6	8.4	4.5	5.3	4.5	5.4	6.5	8.0	6.0	7.7
ARNT2	3.2	4.1	3.4	3.7	3.7	4.1	3.4	3.9	2.8	3.3	2.5	2.8	3.6	3.3	3.9	3.7	3.8	3.6	3.9	3.4	4.2	4.3	4.5	3.4	4.1	4.3	3.7	4.0	4.6	4.6	4.9	3.5
BAPX1	6.0	3.9	5.0	4.0	2.6	3.3	3.6	3.6	6.7	5.8	6.7	6.0	4.0	3.5	3.3	3.9	3.9	2.5	3.5	1.7	3.4	2.8	3.6	2.0	2.6	2.3	3.6	3.4	4.3	2.9	2.4	2.8
BCL6	7.5	7.6	7.5	7.5	6.6	7.3	6.7	7.2	7.7	7.0	7.1	7.2	6.8	7.2	6.4	7.3	8.3	7.0	8.5	6.9	6.0	6.5	6.2	6.4	8.5	8.1	8.5	8.1	6.1	6.7	6.5	6.2
BDKRB1	5.1	3.2	4.8	3.6	3.2	4.1	3.4	3.4	7.1	5.8	6.5	5.7	3.5	3.2	2.6	2.4	2.6	2.9	3.0	3.1	3.3	5.2	1.7	5.3	2.8	3.1	3.1	3.1	3.4	5.2	1.8	4.9
BEX1	4.5	4.3	4.6	4.6	4.8	4.1	4.9	4.5	4.8	4.5	4.2	4.7	4.5	4.5	4.3	4.3	4.5	3.7	4.6	3.4	4.8	3.8	5.3	4.1	5.0	4.1	4.2	4.3	5.0	4.5	5.2	4.8
BGLAP	5.0	4.6	4.6	4.5	7.4	6.9	7.4	7.1	4.3	4.3	4.2	4.3	6.7	7.0	6.4	6.7	5.2	4.4	5.2	4.3	8.3	8.0	8.7	8.2	5.5	4.7	5.5	4.3	7.8	7.1	7.6	7.5
BGN	13.1	13.5	13.0	13.4	13.2	13.2	13.3	13.3	12.7	13.3	12.6	13.5	13.6	13.7	13.7	13.7	11.5	10.8	11.5	10.7	12.3	10.9	12.3	10.9	11.2	10.9	11.3	10.9	12.1	11.8	12.1	12.1
BID	4.5	4.6	4.5	4.6	4.3	4.4	4.3	4.5	4.6	4.3	3.8	4.4	4.4	4.3	3.3	4.5	4.5	4.7	5.0	4.8	4.3	4.5	4.4	4.3	4.8	4.6	4.9	4.7	4.6	4.6	5.0	4.9
BMP2	9.0	10.0	9.0	9.9	10.1	10.9	10.4	10.8	7.7	8.6	7.8	8.6	8.8	9.8	9.2	10.2	7.5	4.3	7.4	4.6	6.6	8.6	6.5	8.7	8.1	5.2	8.1	5.4	6.2	7.8	6.1	8.4
BMP4	5.3	6.2	5.2	6.2	5.8	5.7	5.7	6.0	4.3	7.1	4.1	7.2	7.3	7.6	7.3	7.6	5.2	5.1	5.2	4.6	4.6	6.1	4.6	6.3	5.7	6.1	5.8	6.0	4.4	6.2	3.7	6.9
BMP6	3.8	4.4	3.7	4.0	3.7	3.5	3.6	2.8	4.1	3.3	3.6	3.4	3.8	2.6	3.4	2.7	5.7	3.4	6.2	3.0	3.7	5.6	4.1	5.5	6.7	4.6	6.8	4.7	2.1	4.1	3.5	5.1
BMP7	3.3	2.6	2.8	2.8	4.1	2.2	4.3	2.4	3.1	3.3	3.1	3.9	4.1	3.7	4.2	3.4	2.1	1.2	1.5	1.4	3.5	1.5	3.8	2.0	3.0	2.2	2.8	1.4	3.9	2.0	3.7	1.5
BMPR1A	7.3	7.1	7.1	7.0	7.4	7.2	7.3	7.3	6.3	6.7	6.2	6.7	7.3	7.4	7.4	7.4	8.1	7.1	8.1	7.1	8.2	6.9	8.3	6.9	8.0	7.5	8.0	7.6	8.7	7.3	8.6	7.2
BMPR1B	2.9	3.2	3.0	2.9	2.0	3.3	3.3	3.0	3.1	3.7	2.9	3.6	3.4	3.3	2.8	3.1	2.7	5.4	2.6	5.6	4.3	3.7	4.2	3.2	2.4	4.9	2.8	4.7	3.4	3.9	4.3	4.4
BMPR2	7.3	7.2	7.4	7.2	7.9	7.4	7.9	7.4	6.6	6.6	6.5	6.7	7.3	7.4	7.4	7.4	7.7	6.4	7.6	6.3	8.4	6.5	8.5	6.6	7.6	6.6	7.5	6.6	8.9	6.9	8.9	6.8
BST1	2.9	3.4	2.8	2.5	3.0	3.0	2.5	3.2	3.3	2.8	2.8	2.7	3.0	3.1	3.5	3.1	4.8	5.3	5.2	5.5	4.3	3.9	4.3	3.7	5.4	6.0	5.0	5.8	4.4	4.8	4.9	4.5
C1QTNF3	11.1	8.8	10.8	9.0	5.6	4.4	4.5	4.9	12.4	11.5	12.4	11.4	7.6	6.2	7.3	5.9	6.4	4.4	6.3	4.2	5.3	3.7	5.4	3.2	8.1	5.3	7.9	5.4	5.6	4.5	5.6	5.1
CA12	8.8	8.1	8.5	7.9	6.2	7.1	6.1	7.0	9.3	9.1	9.3	9.4	8.3	7.8	8.0	8.1	6.8	8.5	6.6	8.4	6.0	8.6	5.9	8.5	6.7	7.5	6.7	7.6	5.8	8.6	5.5	8.5
CA5B	5.6	5.3	5.5	5.2	6.6	5.7	6.5	5.4	4.7	4.6	4.6	4.7	5.7	5.8	5.7	5.6	5.8	4.1	5.9	4.5	6.3	5.2	6.1	5.4	6.2	4.9	6.4	4.9	5.4	4.7	5.8	5.1
CA9	7.9	7.5	7.8	7.4	4.8	3.6	4.8	3.1	8.8	7.6	8.4	7.8	4.9	3.7	5.2	4.1	3.9	4.1	2.6	3.2	4.1	1.9	4.2	1.8	4.4	2.8	4.4	3.3	4.6	3.9	4.5	2.8
CARD10	2.6	2.0	2.5	1.9	2.7	2.3	2.6	2.7	2.6	2.6	2.5	2.3	2.6	2.1	2.7	1.7	2.3	3.7	2.8	4.2	3.3	3.6	2.4	4.1	2.9	2.8	2.9	3.5	3.4	4.1	3.5	3.1
CASC3	7.3	7.2	7.2	7.1	7.2	7.2	7.2	7.2	6.7	6.9	6.7	7.0	7.3	7.3	7.3	7.4	7.8	7.3	7.7	7.2	7.8	7.2	7.9	7.0	7.7	7.9	7.6	7.8	7.9	7.2	7.9	6.7
CASP3	5.1	5.7	5.0	5.4	6.0	5.6	5.8	5.6	5.0	4.9	4.8	5.1	5.7	5.9	5.4	5.6	6.2	6.1	6.4	6.2	6.2	6.3	6.5	6.0	5.9	6.8	6.1	7.0	6.3	7.0	6.8	6.7
CASP4	5.0	5.6	4.9	5.6	5.7	5.8	5.6	5.9	3.5	4.2	3.0	4.3	5.2	5.7	5.3	5.7	5.8	6.0	5.9	6.0	6.2	5.6	6.4	5.4	5.9	6.6	5.9	6.7	6.4	5.6	6.7	6.4
CBFB	9.3	8.8	9.3	8.9	9.3	8.7	9.4	8.6	9.0	9.1	9.1	9.1	9.3	8.8	9.3	8.9	9.5	7.5	9.4	7.5	10.2	8.1	10.2	8.1	9.2	7.8	9.1	7.9	10.2	8.2	10.2	8.2
CCL2	1.6	2.2	1.8	2.2	3.0	4.2	3.1	4.0	2.0	2.2	1.5	1.4	3.0	2.7	3.3	3.4	2.9	3.7	3.3	3.8	3.8	5.4	3.1	5.1	2.4	3.6	2.3	3.6	4.1	6.4	4.1	4.2
CCNA2	5.4	6.2	5.3	6.1	3.9	4.3	4.3	4.6	4.1	5.2	4.6	4.9	5.0	4.8	4.9	4.7	4.4	3.4	4.3	3.3	4.1	3.7	3.9	2.8	4.4	3.6	4.5	3.8	4.3	4.6	4.3	3.4
CCNB1IP1	4.0	4.4	4.2	4.1	4.3	3.9	3.5	3.6	4.5	3.9	4.1	4.1	4.2	4.2	3.8	4.0	4.9	4.7	5.0	4.6	4.8	4.4	5.1	4.3	4.9	5.3	4.6	5.6	4.8	4.7	4.8	2.5
CCNB2	5.8	6.3	5.5	6.3	4.3	4.6	3.9	4.3	4.2	5.3	4.4	5.0	4.5	4.7	4.4	4.6	4.4	2.8	4.4	2.4	4.1	3.3	3.7	3.4	4.5	3.8	4.5	3.2	4.7	2.5	4.2	4.0
CCND1	9.3	9.7	9.1	9.7	9.7	10.0	9.7	10.0	9.0	9.3	9.0	9.2	9.4	9.7	9.4	9.7	9.4	9.6	9.5	9.5	9.7	10.7	9.6	10.7	9.3	7.9	9.2	8.1	9.6	10.5	9.6	10.4
CCND2	7.8	6.5	7.6	6.6	6.2	5.2	5.0	5.2	8.1	7.6	8.1	7.6	6.5	5.9	6.8	5.8	6.5	7.6	6.6	7.4	6.3	5.8	6.4	5.7	6.0	6.0	5.9	6.2	6.4	4.9	5.8	5.1
CCNF	4.8	5.2	4.8	5.3	3.7	4.1	3.8	4.1	5.1	4.9	4.7	4.7	4.0	4.1	4.2	4.7	4.4	3.9	4.3	3.8	3.7	3.6	3.9	3.2	4.0	3.7	4.2	4.0	4.2	3.9	3.9	4.2
CCNYL1	5.2	4.8	4.9	5.2	4.9	4.5	4.8	4.7	4.8	5.2	4.9	5.3	5.1	5.3	5.3	4.8	5.5	5.1	5.3	5.0	4.8	4.6	4.8	4.8	5.3	5.2	5.4	5.2	5.5	5.5	5.5	5.4
CD109	8.5	8.1	8.3	8.1	7.2	7.2	7.2	7.2	9.6	8.5	9.3	8.6	7.3	7.1	7.1	7.1	7.6	7.9	7.4	8.0	7.1	8.1	7.0	8.0	8.0	8.0	7.8	7.9	6.8	8.0	6.8	8.0
CD24	7.8	6.6	7.4	6.7	8.7	6.5	8.8	6.9	8.3	8.7	8.2	8.9	9.6	8.1	9.5	8.1	5.6	4.3	5.7	4.2	10.9	4.8	10.7	3.9	6.9	4.3	7.0	5.0	11.0	5.9	11.2	6.5
CD248	6.6	7.3	6.7	7.3	6.1	6.8	6.1	6.9	5.6	6.6	5.5	6.7	6.3	7.1	6.3	7.1	8.2	9.3	8.1	9.3	7.5	7.4	7.5	7.1	8.6	9.7	8.4	9.8	7.9	8.1	8.0	8.0
CD44	7.0	7.9	6.9	8.0	8.2	8.4	8.3	8.2	6.9	6.6	6.8	6.6	7.3	7.9	7.8	8.0	7.2	8.3	7.1	8.2	7.6	8.5	7.4	8.5	7.0	8.3	7.0	8.3	6.6	8.5	6.5	8.5
CD55	2.9	3.2	2.5	2.3	2.9	3.0	3.4	3.4	4.1	3.5	3.4	3.7	3.6	3.6	2.6	3.4	2.8	3.1	3.3	3.5	3.5	3.9	3.2	3.2	4.1	2.8	3.3	3.6	4.4	2.7	3.7	3.0
CD59	9.8	9.9	9.7	9.9	9.5	9.5	9.5	9.5	9.2	9.7	9.4	9.8	9.4	9.5	9.3	9.5	9.4	10.2	9.5	10.1	9.8	9.5	9.8	9.6	9.3	9.4	9.2	9.5	9.4	9.5	9.6	9.7
CD74	5.8	5.1	5.7	5.0	6.4	6.5	6.1	6.6	6.0	5.7	5.8	5.8	6.2	6.5	6.4	6.5	6.0	6.1	6.1	6.1	7.7	8.7	7.6	8.6	6.1	6.2	6.0	6.0	7.5	8.7	7.3	8.6
CD9	9.0	8.9	9.0	8.9	8.2	8.3	8.3	8.2	8.9	8.7	9.0	8.7	8.0	8.1	7.9	8.2	8.0	8.6	8.1	8.7	7.8	8.6	7.7	8.6	8.8	8.4	8.7	8.4	6.5	8.2	7.1	8.4
CDH11	11.1	11.5	11.1	11.5	11.3	11.0	11.3	11.1	9.1	10.5	9.1	10.4	11.4	11.6	11.4	11.6	11.9	10.5	11.9	10.5	12.1	10.8	12.1	10.7	11.9	11.3	11.8	11.3	12.4	11.4	12.3	11.4
CDH13	4.0	4.5	4.0	4.2	2.8	2.9	2.7	3.2	3.1	2.4	2.1	3.2	2.0	2.2	1.8	2.7	4.3	4.6	4.3	4.9	3.3	5.0	2.8	5.2	3.8	4.2	3.6	4.6	4.0	5.0	2.3	5.7
CDH15	7.0	6.1	6.8	5.9	7.1	5.3	7.2	5.9	6.3	6.8	6.0	6.7	8.3	7.6	7.9	7.5	4.9	1.2	5.3	1.6	5.4	3.4	5.5	2.8	7.2	2.1	7.0	3.0	6.2	4.1	6.5	4.1
CDH2	6.4	6.5	6.1	6.5	6.6	5.9	6.4	6.1	6.9	6.9	6.7	6.9	7.4	6.8	7.4	7.0	7.4	5.2	7.4	5.1	8.0	6.1	8.2	5.9	8.4	5.3	8.5	5.6	8.8	6.7	8.8	7.0
CDKN1A	8.7	8.9	8.7	8.9	9.8	9.9	9.8	9.8	8.8	8.5	8.7	8.5	9.4	9.3	9.6	9.6	8.9	9.7	8.9	9.7	9.4	10.3	9.4	10.2	9.1	9.8	9.1	10.0	9.2	10.2	9.2	10.3
CDKN1B	4.2	4.2	4.6	4.1	4.7	4.1	4.3	3.7	4.1	3.9	4.0	4.2	4.2	4.3	4.3	3.8	5.1	3.9	4.7	4.0	5.0	3.6	5.0	3.9	4.9	5.4	4.8	4.7	4.8	4.2	4.6	4.7
CDKN1C	8.3	5.4	7.7	5.0	3.8	3.9	3.9	3.4	10.1	9.0	10.0	9.2	6.5	5.9	6.3	5.7	5.2	4.4	5.4	4.5	5.6	3.3	5.4	4.0	7.0	6.1	7.1	6.1	7.1	5.1	6.8	5.2
CDKN2A	5.0	5.0	5.2	5.3	5.1	5.4	5.7	5.7	4.5	4.9	4.6	4.7	5.2	5.3	5.5	5.3	5.9	6.1	6.0	5.9	6.1	6.0	6.3	6.3	5.6	5.5	5.8	5.3	6.4	5.5	6.3	5.9
CDKN2B	5.7	5.0	5.7	5.3	7.2	7.2	7.6	7.2	4.6	5.3	4.6	5.0	6.4	6.5	6.6	6.5	6.9	6.6	6.8	6.4	7.2	7.8	7.3	8.1	6.4	5.6	6.5	5.6	6.9	6.8	6.7	6.7
CDKN2C	5.2	5.3	5.0	5.3	4.5	4.1	4.0	4.2	4.5	4.5	4.5	4.4	4.3	3.7	4.2	4.4	5.2	4.3	5.2	4.4	3.9	3.9	4.0	4.2	5.7	5.3	5.7	5.3	4.9	4.9	4.3	3.4
CDKN2D	4.2	4.3	4.3	4.3	3.9	3.6	4.5	4.1	3.9	3.7	3.9	4.0	3.6	4.1	4.2	3.6	4.4	3.8	4.3	3.7	4.3	4.1	4.5	4.6	3.9	3.5	3.5	3.9	4.1	3.8	4.5	2.4
CEBPA	1.2	0.5	1.9	1.3	1.0	1.5	2.7	1.2	2.2	1.6	1.6	1.3	2.0	1.6	1.6	2.2	5.0	1.9	5.1	2.4	2.7	2.6	3.6	2.3	6.2	4.0	5.8	4.4	3.7	2.3	3.8	3.0
CEBPB	8.6	8.5	8.5	8.4	8.8	9.1	9.0	9.0	7.7	7.6	7.6	7.7	8.2	8.7	8.4	8.9	9.1	7.7	9.1	7.6	8.7	8.6	8.8	8.5	9.3	8.3	9.2	8.5	9.1	8.8	9.1	8.6
CHAD	10.2	6.6	9.5	6.5	5.3	3.6	4.4	3.3	11.6	9.9	11.6	10.4	8.5	6.1	8.3	5.4	4.6	3.1	4.4	2.3	4.7	2.3	4.6	2.7	8.5	3.9	8.9	5.3	5.2	3.2	6.1	5.0
CHST1	6.7	7.3	6.8	7.3	6.9	6.9	7.1	7.0	6.0	7.1	5.8	7.3	6.8	7.5	6.8	7.1	5.2	4.5	4.8	4.4	5.9	6.0	6.2	6.1	4.8	4.2	4.6	4.1	5.6	5.3	5.5	5.8
CHST3	8.3	7.6	8.1	7.5	7.1	6.6	6.9	6.4	8.8	9.1	8.8	9.2	8.3	7.5	8.3	7.5	6.1	5.8	5.9	5.8	6.2	5.7	6.4	6.1	6.9	6.3	6.9	6.4	6.1	6.0	6.6	6.4
CHST6	6.2	5.9	6.1	6.0	5.9	6.0	5.4	6.2	6.2	7.0	6.4	6.8	7.1	7.1	6.8	7.0	3.4	2.0	3.5	1.3	3.9	3.2	4.1	2.1	3.2	2.4	4.3	1.6	3.9	1.7	4.4	3.3
CITED2	6.8	6.9	6.8	6.7	7.3	6.7	7.4	6.8	6.3	6.1	6.2	6.4	7.2	7.1	7.0	7.3	9.5	9.1	9.6	9.1	8.4	7.9	8.4	7.9	9.5	9.7	9.4	9.7	8.5	8.2	8.6	8.2
CITED4	3.2	3.9	3.3	3.9	2.9	4.0	3.9	3.4	4.0	4.0	4.6	3.8	3.7	4.2	2.5	4.0	2.4	6.1	2.5	5.7	3.8	4.5	2.6	4.9	2.5	3.5	2.2	3.5	2.9	4.2	2.9	4.7
CNN1	3.9	3.9	4.1	3.7	3.3	3.0	2.2	3.1	5.5	4.2	5.0	4.3	3.5	2.8	3.3	3.7	4.3	4.0	4.4	4.3	4.1	2.5	3.7	3.2	4.1	4.1	4.1	4.7	4.2	3.8	3.7	3.0
CNN2	8.1	9.3	8.2	9.2	8.1	8.6	8.3	8.9	7.0	8.2	6.8	8.1	8.5	9.0	8.4	8.9	9.2	9.5	9.2	9.4	8.9	8.3	8.9	8.2	9.0	10.0	8.9	10.0	9.1	9.1	8.8	9.3
COL10A1	14.9	14.3	14.4	14.3	14.0	13.3	13.9	13.0	15.6	15.3	15.6	15.7	14.4	13.8	14.4	13.9	3.5	2.3	3.6	2.4	5.0	6.0	5.0	5.8	9.0	2.8	9.0	2.0	5.7	6.4	5.9	6.8
COL11A1	12.9	11.5	12.5	11.5	8.9	8.0	7.7	7.8	14.1	13.6	13.9	13.7	11.7	10.3	11.2	9.8	9.4	7.9	9.4	7.4	7.3	6.1	7.4	6.1	11.1	9.4	11.2	9.5	8.9	7.2	8.8	8.1
COL11A2	10.8	7.7	10.3	8.0	5.9	3.2	5.0	3.0	12.4	11.4	12.2	11.5	9.8	6.9	9.1	6.4	4.2	1.4	4.2	1.4	3.3	2.5	4.1	1.7	7.5	2.9	7.5	1.6	4.6	1.5	5.6	1.3
COL12A1	12.1	11.7	11.8	11.6	10.9	9.8	10.9	10.0	12.7	13.1	12.5	13.3	12.2	11.5	11.8	11.1	11.0	8.7	11.0	8.6	10.8	8.4	10.8	8.3	12.1	9.8	12.0	9.8	11.3	9.8	11.4	10.1
COL14A1	4.5	5.0	4.3	5.1	5.5	7.9	5.9	8.3	4.6	5.3	4.4	5.4	5.1	7.5	5.2	7.8	4.0	8.8	4.2	8.8	5.8	10.2	6.2	10.3	4.7	8.5	4.2	8.3	6.9	10.6	6.6	10.5
COL18A1	6.4	6.7	6.3	6.7	7.3	6.9	7.4	6.8	4.5	5.5	4.4	5.0	6.1	6.5	6.1	6.7	6.3	8.3	6.0	8.3	6.7	7.2	6.5	7.1	5.3	8.6	5.1	8.6	6.1	7.3	5.9	7.4
COL1A1	15.9	15.9	15.7	15.7	14.8	14.0	14.6	14.2	16.0	16.6	15.8	16.8	16.2	15.8	16.0	15.7	14.1	12.5	14.3	12.4	14.8	11.6	15.0	11.4	15.1	13.3	15.2	13.4	14.9	13.2	14.7	13.8
COL1A2	15.2	15.1	15.0	15.0	14.4	13.5	14.3	13.7	15.3	15.8	15.0	16.0	15.6	15.2	15.4	15.1	14.0	13.4	14.1	13.3	14.5	12.3	14.6	12.2	14.8	14.2	14.9	14.2	14.7	13.4	14.5	13.7
COL27A1	8.4	6.7	8.0	6.4	6.5	6.8	6.4	6.6	9.2	8.3	9.2	8.2	7.7	6.9	7.5	7.0	6.8	6.0	6.9	5.9	7.2	6.5	6.8	6.4	7.8	7.0	7.4	6.9	7.3	7.6	7.1	7.5
COL2A1_1	11.4	8.7	10.9	8.9	7.0	4.1	5.7	3.9	12.6	12.2	12.3	12.2	10.5	7.8	9.9	7.1	4.3	1.6	4.8	1.6	4.2	1.5	4.5	2.7	7.1	2.1	7.5	2.2	5.8	2.0	5.6	3.9
COL2A1_2	13.4	9.7	12.8	9.9	6.7	3.7	5.3	3.6	15.2	14.2	15.0	14.2	11.1	7.6	10.4	6.7	3.7	0.9	4.3	1.3	2.4	2.3	1.4	2.3	7.6	2.7	8.8	2.4	6.2	2.0	5.8	3.3
COL2A1_common	15.0	11.6	14.4	11.9	9.1	5.0	7.1	4.5	16.7	15.8	16.6	15.9	13.1	9.9	12.7	9.1	4.6	0.9	5.6	2.5	3.0	2.2	3.7	2.2	9.9	2.6	10.3	2.5	6.6	2.6	7.9	4.6
COL3A1	16.2	16.2	16.0	16.1	15.9	15.6	16.1	15.7	13.0	14.1	12.7	14.1	14.9	15.6	15.1	15.9	17.7	15.8	17.7	15.8	17.3	14.1	17.2	14.0	16.4	16.7	16.4	16.8	17.0	15.0	16.9	15.1
COL4A1	6.4	7.5	6.1	7.4	6.2	6.7	6.2	6.9	5.9	5.7	5.7	5.4	4.2	5.0	4.3	5.1	7.9	7.3	8.0	7.3	5.9	5.6	5.7	5.6	6.9	8.2	7.0	8.4	4.7	6.0	5.1	6.2
COL4A2	5.0	6.4	5.1	6.1	4.8	5.4	4.9	5.5	4.5	4.3	4.4	3.7	3.2	3.8	3.7	4.5	6.7	6.4	6.7	6.4	4.9	5.1	4.5	5.1	5.8	7.2	5.5	7.4	4.1	5.5	3.6	5.8
COL6A1	11.2	11.9	11.1	11.8	11.1	11.9	11.1	12.0	11.2	10.8	11.1	10.7	10.4	11.4	10.7	11.5	11.7	12.4	11.6	12.2	11.3	12.6	11.2	12.6	11.0	12.2	11.0	12.3	10.7	12.8	10.5	13.0
COL7A1	8.9	9.9	8.8	9.8	9.7	9.6	9.8	9.5	8.0	8.1	7.6	8.3	8.9	8.9	8.5	9.2	7.5	7.9	7.7	7.8	7.9	7.7	7.7	7.6	7.4	8.2	7.2	8.4	7.0	7.7	7.2	7.9
COL8A1	9.6	10.3	9.4	10.3	9.6	9.9	9.6	9.9	8.2	9.2	8.0	9.2	9.6	9.8	9.4	9.8	9.5	7.6	9.6	7.6	9.5	8.3	9.3	8.5	10.3	7.3	10.3	7.5	11.1	8.8	11.3	8.8
COL9A1	8.7	3.9	8.0	4.0	1.8	0.9	0.9	1.7	10.8	9.5	10.8	9.6	5.3	3.1	4.6	1.5	0.9	0.8	1.9	1.4	1.5	2.2	1.2	1.4	4.5	1.7	5.0	1.4	2.6	2.0	1.8	2.1
COL9A2	10.8	8.6	10.3	8.6	8.2	5.1	7.6	4.6	11.7	11.1	11.7	11.2	9.9	7.6	9.5	7.2	6.9	2.6	7.1	2.4	8.9	2.3	9.1	2.4	8.4	3.8	8.3	3.9	9.1	3.2	9.3	3.3
COL9A3	10.5	7.8	10.1	8.2	6.3	4.4	6.0	4.0	12.0	10.9	11.8	10.9	8.9	6.5	8.4	6.1	5.2	1.6	4.9	1.6	4.3	2.0	4.0	0.8	7.6	1.5	7.5	3.2	6.2	3.2	5.6	0.8
COMP	14.3	14.6	14.3	14.6	12.3	12.1	12.2	11.7	12.9	13.8	12.9	14.0	12.8	12.4	12.7	12.4	9.3	6.3	9.1	6.1	5.2	4.1	5.5	4.9	9.4	8.2	9.1	7.9	6.9	5.7	6.9	5.9
CP	2.3	2.6	2.4	2.4	2.5	1.9	2.6	2.0	2.7	2.6	2.7	2.8	1.9	2.9	2.5	2.5	3.6	2.8	3.1	2.4	2.9	2.2	1.9	2.4	3.6	3.7	3.9	3.3	2.2	3.8	2.6	1.1
CRLF1	9.7	8.7	9.7	8.7	9.7	8.4	9.8	8.3	8.9	7.7	8.8	8.1	9.5	8.2	9.4	8.2	6.6	3.1	6.5	3.4	7.3	3.2	6.8	3.6	6.8	4.5	6.8	4.4	7.2	4.1	7.7	3.9
CRTAC1	5.0	5.5	4.7	5.4	5.5	5.6	5.0	4.9	4.3	5.5	4.1	5.5	5.2	5.6	5.4	5.6	5.2	1.6	5.0	1.8	4.0	2.8	4.0	2.7	5.9	2.2	5.6	2.9	4.8	2.9	5.4	3.0
CRTAP	4.7	4.9	4.8	4.7	5.0	4.5	4.9	4.7	4.4	4.9	4.5	5.2	4.9	5.1	4.6	4.8	4.7	4.7	4.7	4.5	4.7	3.7	5.0	2.6	5.2	5.0	4.9	4.8	4.9	4.1	4.8	5.0

More than 10 fold upregulated
2-10 fold upregulated
0-2 fold upregulated
Not significantly regulated
0-2 fold downregulated
2-10 fold down-regulated
More than 10 fold downregulated

CRYGS	6.9	5.5	6.9	5.4	5.7	5.7	5.5	5.7	6.8	6.4	7.1	6.3	6.2	6.0	6.0	5.8	5.4	3.4	5.3	3.7	5.6	4.3	5.8	3.7	6.6	4.0	6.2	4.3	6.6	4.5	7.6	3.0
CTGF	12.2	11.5	12.0	11.5	9.3	8.6	9.0	8.8	13.5	12.8	13.1	12.9	10.9	9.5	10.8	9.3	8.6	7.5	8.7	7.4	8.9	8.2	8.6	8.1	9.7	8.5	9.6	8.6	9.0	9.1	8.6	9.1
CTNNB1	12.3	12.5	12.2	12.5	11.2	11.3	11.2	11.4	11.9	12.5	11.8	12.7	11.8	11.8	11.7	11.7	11.3	10.4	11.3	10.4	11.1	11.1	11.1	11.0	11.9	10.9	11.8	10.9	11.5	11.2	11.4	11.5
CXCL12	3.8	4.6	3.7	4.5	7.3	8.8	7.6	8.9	3.0	3.7	3.3	3.4	5.1	7.3	6.1	8.0	5.5	7.9	5.0	7.8	6.4	8.3	6.4	8.1	5.6	9.1	5.9	9.2	6.6	10.1	6.1	10.1
CXCL14	8.2	5.9	7.7	5.8	3.0	1.9	3.0	2.2	9.6	9.0	9.5	9.3	6.5	6.3	6.1	5.6	1.3	1.0	3.2	1.3	2.3	2.2	3.1	2.2	4.1	1.3	3.6	1.2	3.9	2.0	3.5	1.1
CXCL16	3.5	3.5	3.6	3.5	4.2	3.9	4.2	3.9	3.8	3.4	3.2	3.8	3.1	4.1	3.6	3.5	4.2	4.0	4.0	3.5	5.4	4.8	5.2	4.4	3.7	4.2	3.0	3.6	5.6	4.8	5.1	4.9
CYB5B	7.8	7.7	7.7	7.8	7.4	7.4	7.5	7.6	7.6	8.0	7.8	7.8	7.8	7.7	7.8	7.6	7.9	7.5	7.9	7.5	8.0	7.7	8.0	7.6	7.6	7.5	7.7	7.4	7.9	7.4	8.2	7.4
CYC1	6.3	6.3	6.4	6.4	5.9	5.8	6.0	5.7	6.3	6.6	6.6	6.5	6.8	6.5	6.6	6.3	6.1	5.8	6.2	6.0	6.0	5.9	6.3	5.5	6.0	5.6	6.3	6.0	6.2	5.4	6.3	6.0
DCN	12.8	13.1	12.9	13.0	11.2	11.2	11.3	11.2	12.3	12.1	12.1	12.2	10.9	11.5	11.0	11.7	13.3	12.2	13.3	12.2	10.7	9.8	10.8	9.6	13.1	13.0	13.1	13.0	10.7	10.6	10.6	10.8
DDIT3	5.7	5.7	5.9	5.7	6.5	6.4	6.6	6.5	6.9	6.0	6.3	6.2	6.6	6.2	6.3	6.4	6.4	6.5	6.4	6.4	7.1	6.7	7.3	6.5	6.7	7.2	6.9	7.5	7.4	7.3	7.4	6.7
DKK1	6.3	4.4	5.4	4.6	4.7	2.9	4.9	2.3	6.7	6.2	6.7	6.0	6.7	4.5	6.2	4.2	6.1	2.3	6.4	2.9	6.7	2.0	7.2	2.5	5.4	4.1	5.7	4.4	4.9	3.1	5.4	2.7
DKK2	3.0	4.2	2.1	3.8	3.9	3.0	4.3	3.3	2.6	2.8	2.1	2.6	4.0	3.3	4.1	2.4	2.9	1.8	3.1	2.7	3.9	2.3	3.5	1.7	3.0	3.0	3.3	2.3	3.3	3.6	4.6	2.9
DLX5	8.4	8.5	8.4	8.4	7.4	6.4	7.5	6.4	7.7	8.0	7.5	8.2	7.8	7.7	7.8	7.7	9.0	5.3	9.1	5.4	9.2	5.1	9.2	4.6	8.8	6.3	8.9	6.3	9.8	5.4	9.9	5.6
DPT	9.0	9.7	9.0	9.5	7.3	5.6	7.4	5.4	7.1	9.1	7.1	9.3	7.7	7.3	7.4	7.1	7.3	8.0	7.4	8.0	6.3	3.3	6.3	2.9	8.3	10.1	8.1	10.0	6.5	4.5	6.6	4.7
EFNA1	5.6	5.7	5.6	5.4	5.4	4.5	5.5	4.4	6.4	5.8	6.1	6.1	5.5	5.4	5.4	5.2	5.5	3.8	5.9	4.1	5.0	3.7	5.2	3.5	6.3	4.7	6.3	4.4	5.3	4.6	5.4	2.7
EFNB2	3.2	4.3	3.3	4.3	3.6	3.5	3.6	3.2	3.9	3.2	3.5	3.2	3.1	3.5	3.6	4.0	2.3	5.3	3.3	5.1	3.8	3.7	3.9	3.4	2.4	3.1	3.7	3.6	5.0	3.7	5.3	2.9
EIF2B1	7.3	7.3	7.5	7.3	7.4	7.0	7.1	7.0	7.2	7.3	7.1	7.2	7.3	7.3	7.4	7.2	7.7	7.3	7.7	7.2	7.3	6.8	7.4	6.8	7.6	7.5	7.5	7.4	7.5	7.1	7.6	7.2
ELF1	7.1	6.8	7.0	6.8	6.8	6.9	7.0	6.8	6.4	6.4	6.4	6.7	6.5	6.8	6.8	6.8	7.6	6.8	7.6	6.8	7.0	6.3	7.3	6.6	7.6	7.2	7.6	7.1	7.5	6.7	7.3	7.1
ELN	4.4	5.8	4.3	5.4	3.8	4.0	3.8	3.9	4.8	5.4	4.7	5.5	4.8	6.3	5.1	5.7	3.8	2.9	3.5	3.1	3.5	2.6	3.6	3.1	4.5	4.5	3.8	3.3	4.7	3.5	4.3	4.2
EPHA2	6.8	7.2	6.7	7.1	7.7	6.9	7.7	7.3	5.8	5.8	5.3	5.8	7.9	7.6	7.8	7.8	5.9	5.0	6.2	4.9	7.4	5.4	7.4	5.9	6.3	5.2	6.3	5.8	7.9	5.8	7.6	6.2
EPYC	8.4	6.4	8.1	7.0	6.4	5.0	5.9	5.3	9.6	9.2	9.5	9.0	7.0	6.4	6.6	6.6	3.1	1.4	2.6	1.1	3.0	1.0	3.2	0.9	4.5	1.5	4.5	1.4	3.5	2.1	4.0	3.5
ERF	8.1	7.8	8.1	7.8	7.8	7.7	7.7	7.6	8.0	8.4	8.2	8.3	8.6	8.5	8.6	8.4	8.3	6.7	8.2	6.5	8.2	6.8	8.0	7.2	8.2	6.7	8.1	6.6	8.0	6.9	8.0	7.1
FGF11	6.9	6.4	6.6	6.1	5.2	4.8	4.9	4.9	6.9	6.9	7.0	7.1	5.9	5.4	5.6	5.3	4.2	2.6	4.3	2.7	4.4	1.8	4.0	3.1	4.7	3.6	4.6	3.6	4.4	3.7	5.0	2.4
FGF2	6.2	5.7	5.8	5.8	5.8	5.2	5.9	5.1	6.6	6.3	6.5	6.3	5.8	4.8	5.9	5.0	4.9	5.3	4.9	4.9	5.9	5.4	5.8	5.5	5.3	5.2	4.9	4.8	6.1	5.4	6.4	5.5
FGF7	6.5	6.9	6.5	6.7	7.7	8.1	8.2	8.0	5.5	5.5	5.5	5.9	5.6	6.9	5.9	7.0	8.6	7.2	8.6	7.0	9.1	8.0	9.0	8.0	8.7	8.0	8.6	7.9	8.7	8.8	8.7	8.9
FGFBP2	8.0	6.7	7.7	6.6	5.1	3.8	4.5	3.2	9.9	9.7	9.7	9.9	7.4	6.6	7.3	6.0	3.9	2.6	3.9	2.7	2.9	3.0	3.2	3.2	5.5	3.8	5.6	3.9	3.4	3.3	4.0	3.6
FGFR1	9.6	9.7	9.4	9.6	11.0	10.9	11.0	11.2	7.9	9.0	7.7	9.1	10.0	10.8	10.0	10.8	8.8	9.1	8.9	9.0	10.9	10.6	10.8	10.5	9.0	9.7	8.9	9.8	10.6	10.3	10.5	10.5
FGFR2	4.7	4.7	4.6	4.5	3.3	3.7	3.2	3.4	4.1	4.6	4.0	4.3	4.8	5.2	5.2	4.4	4.0	2.4	4.2	2.0	4.4	3.7	4.5	1.6	4.8	3.7	5.1	4.0	5.1	4.5	5.0	4.3
FGFR3	10.0	7.1	9.9	7.1	8.5	5.5	8.4	5.5	10.6	9.2	10.8	9.2	9.3	7.2	9.2	7.0	8.8	2.3	8.9	2.0	10.0	1.1	9.9	2.4	9.5	2.6	9.6	2.7	11.4	3.5	11.3	3.6
FGFR4	5.8	4.1	6.0	4.0	3.8	2.6	3.5	2.9	6.2	5.4	6.1	5.4	5.0	3.8	4.7	3.2	3.1	2.5	3.3	2.3	4.2	2.1	3.4	2.3	4.3	3.2	4.4	3.1	3.8	3.0	4.8	4.0
FGFRL1	9.3	8.1	9.2	8.3	9.0	8.3	8.9	8.2	10.0	9.4	10.1	9.4	9.5	8.7	9.3	8.7	7.6	7.3	7.6	7.1	8.0	7.1	7.8	7.1	8.1	7.7	8.0	7.6	8.3	7.3	8.3	7.0
FMOD	12.4	11.0	12.2	11.0	9.5	8.2	8.9	7.9	13.8	13.0	13.6	13.0	11.5	9.9	11.3	9.5	8.1	7.1	8.0	7.1	7.7	5.2	7.5	5.6	9.1	7.7	9.1	7.6	8.7	6.7	8.3	6.7
FN1	16.3	16.5	16.2	16.6	16.4	16.3	16.5	16.2	16.8	16.5	16.4	16.6	16.4	16.5	16.3	16.6	14.6	15.6	14.4	15.5	14.3	15.3	14.3	15.4	14.0	15.1	13.9	14.9	14.0	15.8	13.9	15.7
FOXA2	5.8	2.7	5.2	3.1	3.6	2.1	2.8	3.2	7.4	6.2	7.4	6.3	4.2	2.7	3.5	2.8	2.3	1.6	2.8	1.9	2.8	1.0	2.9	2.4	4.3	1.7	4.2	1.8	3.8	2.0	3.4	1.1
FOXA3	3.6	3.0	3.7	2.8	3.4	2.4	2.9	2.8	4.5	4.3	4.3	4.4	3.9	3.6	4.2	3.2	2.1	1.3	2.4	1.6	3.0	1.6	2.7	1.4	3.3	2.8	3.2	2.7	3.9	2.2	3.4	1.3
FOXC1	5.5	5.7	5.6	5.5	5.1	5.1	5.5	5.0	5.8	5.9	5.9	5.7	6.0	5.8	5.8	5.6	5.6	5.4	5.0	5.6	5.7	5.7	5.2	5.5	5.4	5.6	5.2	6.1	6.0	5.6	6.1	5.2
FOXC2	6.6	6.0	6.7	5.8	7.8	6.2	7.6	6.4	6.2	5.7	6.2	5.8	7.4	6.5	7.3	6.6	7.5	6.5	7.5	6.5	8.7	6.1	8.7	6.4	7.1	6.4	7.1	6.6	9.0	5.9	8.8	5.9
FOXD1	7.4	7.4	7.3	7.3	7.6	7.1	7.7	7.3	7.0	7.5	7.1	7.7	8.0	8.2	8.1	8.2	7.1	6.6	7.0	6.5	7.1	7.2	7.2	7.2	7.0	6.9	6.9	7.1	7.3	7.6	7.3	7.9
FOXD2	2.7	2.4	3.0	2.6	2.0	1.6	2.3	2.2	2.8	3.2	3.1	3.2	2.0	1.5	2.3	2.6	3.4	2.6	3.0	2.4	2.9	2.0	3.2	1.3	4.1	3.0	3.3	3.6	1.6	2.4	1.8	0.2
FOXM1	6.2	6.6	6.1	6.8	4.8	5.1	4.9	5.1	5.2	5.8	5.3	5.4	5.4	5.3	5.1	5.2	4.7	3.3	4.6	3.3	4.5	3.5	3.4	3.8	4.7	4.4	4.6	4.2	5.2	4.6	5.1	5.1
FOXO1	8.3	9.2	8.4	9.2	8.8	9.3	8.9	9.1	6.6	7.2	6.4	7.2	8.0	8.4	8.2	8.8	6.8	7.3	6.4	7.3	5.4	5.7	5.6	5.5	6.9	8.3	6.7	8.4	5.6	5.6	5.1	6.2
FOXQ1	3.3	6.0	3.7	5.9	3.2	3.5	3.2	4.0	2.9	3.1	2.4	2.8	3.0	2.9	2.2	3.1	2.1	2.6	2.9	2.6	3.0	5.7	2.6	5.6	2.8	2.9	3.0	3.2	3.6	6.4	3.6	6.0
FRZB	5.6	4.4	5.5	4.6	5.6	2.9	5.9	3.0	6.3	6.0	6.2	5.7	6.0	4.0	5.6	4.2	6.9	1.9	6.9	2.0	6.2	2.3	6.3	2.2	7.3	2.3	7.3	2.8	6.4	2.6	6.7	2.0
FZD2	5.2	5.2	5.1	5.3	5.1	5.4	5.2	5.5	5.0	4.8	4.7	5.1	4.9	5.4	4.9	5.6	5.7	6.1	5.8	6.0	6.4	5.7	6.5	5.8	5.5	6.2	5.5	6.7	6.4	6.3	6.1	6.5
FZD4	4.8	4.1	4.9	4.4	4.5	4.2	4.6	5.0	4.6	4.4	4.2	3.9	4.2	4.8	3.5	4.3	6.9	6.0	6.9	6.1	5.2	4.6	5.5	4.6	7.6	6.8	7.5	6.9	5.9	5.0	5.8	3.2
FZD6	5.7	6.8	5.9	6.7	6.3	6.0	6.2	6.0	4.6	5.4	4.6	5.5	5.8	5.7	6.0	5.9	5.2	6.5	5.2	6.4	6.1	6.2	5.8	6.2	4.9	6.2	4.8	6.3	5.8	6.0	6.1	6.4
FZD7	6.6	6.9	6.8	6.9	7.4	7.3	7.6	7.6	6.2	6.6	5.9	6.9	6.8	7.7	6.9	7.9	9.1	6.6	9.1	6.6	8.6	7.5	8.6	7.7	8.3	7.4	8.2	7.3	7.5	7.7	7.4	7.5
FZD8	7.3	6.7	7.3	6.8	8.5	7.3	8.3	7.3	6.1	6.4	6.5	6.2	8.2	7.8	8.4	7.7	8.0	4.5	7.9	4.5	8.6	7.2	8.6	7.6	7.9	5.4	7.9	5.5	8.7	7.2	8.8	6.9
FZD9	4.4	2.9	4.1	2.7	3.4	2.1	2.1	2.3	5.2	5.0	5.1	5.1	3.5	3.1	4.9	3.7	0.8	1.7	1.7	1.1	1.0	1.4	0.8	1.2	1.7	0.8	2.3	1.6	0.9	1.5	2.4	1.1
GLI1	4.0	3.7	3.9	3.5	2.6	3.4	3.0	3.4	4.2	4.6	4.1	4.8	4.5	4.9	4.4	4.5	1.8	1.6	1.8	1.5	1.8	2.7	2.9	2.8	2.2	2.4	1.9	1.2	3.6	1.5	3.6	2.7
GLI2	3.4	3.5	3.2	3.4	3.7	4.1	3.8	3.5	2.9	3.3	3.0	3.7	4.3	4.3	4.2	4.3	3.3	2.7	3.7	2.8	3.5	2.2	3.3	1.5	3.2	3.7	3.1	3.3	3.6	2.4	4.4	4.0
GLI3	5.7	6.6	5.7	6.6	6.5	6.8	6.6	6.7	5.0	5.4	5.0	5.7	6.4	6.7	6.5	6.8	6.5	6.4	6.6	6.4	6.9	7.2	7.1	7.1	6.4	7.0	6.4	6.9	7.4	7.5	7.2	7.8
GLI4	2.3	2.5	3.0	2.4	2.6	2.5	2.8	2.5	2.8	2.5	2.5	2.8	3.3	3.1	3.4	2.7	2.7	2.2	3.0	2.3	3.1	2.8	3.5	2.6	3.6	3.5	2.4	2.7	3.1	3.1	4.1	2.6
GLIS3	5.2	5.1	4.8	4.8	4.6	4.6	4.0	3.9	5.9	5.3	5.7	5.3	4.0	3.9	4.3	4.2	4.7	5.4	4.3	5.6	4.7	5.3	4.7	4.8	4.8	5.9	4.8	5.6	4.4	5.8	4.6	5.3
HAPLN1	12.6	9.9	12.3	10.1	6.5	4.7	5.6	4.5	14.0	13.0	13.9	13.1	10.0	7.2	9.5	6.7	6.6	2.4	6.5	2.7	4.7	2.0	4.3	2.9	9.5	4.5	9.7	3.1	6.9	4.3	6.7	4.9
HAPLN3	7.0	6.7	6.9	6.7	6.7	6.7	6.5	6.6	7.4	6.9	7.1	7.1	6.8	7.0	7.1	7.1	5.8	6.3	5.4	6.1	5.9	5.8	6.1	5.9	6.3	7.1	6.0	6.9	6.1	6.3	6.1	6.0
HAS1	7.8	8.8	7.5	8.8	5.6	7.1	5.6	7.0	5.6	5.9	5.3	5.9	4.8	6.0	4.7	6.2	5.9	5.6	6.1	5.2	4.1	5.4	3.0	5.2	4.3	4.1	4.8	4.0	4.3	4.6	4.1	5.6
HAS2	6.7	7.8	6.5	8.0	5.7	8.0	5.8	8.0	7.3	6.9	7.4	7.1	5.6	5.9	5.7	6.1	6.5	9.2	6.3	9.3	4.9	8.1	5.4	8.1	7.5	9.6	7.5	9.4	4.9	8.0	4.8	7.8
HAS3	1.6	1.8	2.1	2.7	2.6	2.0	3.2	2.2	1.9	1.7	1.9	2.4	2.3	2.7	2.8	2.3	3.0	2.2	3.0	1.5	3.6	2.7	2.9	2.8	2.3	2.3	2.0	3.2	3.7	2.9	3.6	4.2
HCLS1	4.7	3.5	4.3	3.9	3.5	3.6	3.7	3.4	5.5	4.7	5.5	4.8	3.9	3.6	4.0	3.6	3.3	3.0	3.1	2.5	3.2	2.8	2.6	1.4	3.6	3.6	2.9	2.6	3.4	3.1	3.6	2.6
HES1	5.5	5.4	5.4	5.3	6.7	6.9	6.9	7.0	5.8	4.6	5.5	4.7	5.7	6.0	5.7	6.5	6.1	3.3	5.8	3.4	6.9	6.7	6.8	6.7	6.0	3.8	6.2	3.1	5.9	7.0	5.8	7.2
HESX1	3.9	3.9	4.1	3.8	3.8	2.9	3.8	3.3	4.5	4.2	4.2	4.6	4.2	3.6	4.1	3.2	3.6	2.8	3.6	3.0	4.3	2.6	3.9	1.4	3.3	3.4	3.1	3.4	4.7	2.2	4.0	4.0
HEY1	8.5	4.6	8.4	4.8	7.3	2.5	7.4	3.1	5.7	4.5	6.0	4.5	7.6	4.3	7.7	3.6	10.6	1.3	10.7	1.7	11.1	1.0	11.1	1.4	10.2	2.3	10.3	2.4	11.5	3.5	11.5	1.4
HEY2	3.6	2.0	3.7	2.1	5.6	3.2	5.7	3.5	3.1	2.8	3.2	2.4	4.6	4.1	5.3	4.1	3.1	2.0	3.1	1.9	6.1	2.6	5.7	2.7	2.9	3.0	3.1	3.1	6.8	2.0	7.0	4.1
HIF1A	10.3	9.8	10.3	10.0	9.7	10.1	9.8	9.8	10.4	10.5	10.6	10.4	10.1	9.7	9.9	9.8	10.7	9.3	10.8	9.2	9.8	9.3	9.9	9.3	10.4	9.1	10.2	9.0	10.1	9.2	10.0	9.0
HIF2A	2.0	1.7	2.1	1.7	2.2	1.8	2.2	1.9	1.5	1.6	1.6	1.6	2.3	2.0	2.1	2.0	2.5	2.0	2.3	1.9	2.6	2.3	2.6	2.3	2.4	2.2	2.5	2.2	2.8	2.4	2.8	2.4
HMBS	5.5	5.4	5.2	5.5	5.2	5.2	5.4	5.2	4.9	5.3	5.2	5.5	5.6	5.5	4.5	5.0	5.2	4.8	5.0	4.9	5.4	4.7	5.4	4.6	5.6	4.6	5.3	4.6	5.3	4.8	6.0	5.4
HP	0.9	1.3	1.0	1.4	1.5	1.9	1.5	1.2	2.0	1.1	1.1	1.6	1.4	1.6	1.9	1.5	3.3	3.5	3.7	3.6	2.0	0.7	1.6	1.9	3.8	4.5	4.1	4.3	3.1	2.1	2.2	1.0
HPRT1	7.6	7.5	7.5	7.4	6.5	6.5	6.6	6.5	7.7	7.5	7.7	7.7	6.7	6.7	6.6	6.6	7.0	6.5	7.0	6.6	6.6	6.2	6.3	6.5	7.0	6.2	7.0	6.2	6.6	6.1	6.3	6.2
HYAL1	4.6	4.5	4.4	4.2	3.9	3.8	3.4	3.7	5.2	4.6	4.9	4.7	4.0	4.4	4.6	4.2	3.7	3.4	3.5	3.1	3.3	3.0	3.9	3.0	3.9	3.1	4.0	3.9	4.2	4.3	3.4	2.8
ICAM1	2.7	2.7	3.1	2.4	3.7	3.4	3.3	3.1	3.3	2.8	3.5	2.8	3.2	3.4	3.7	3.5	3.1	1.8	3.2	2.0	4.0	3.8	4.1	1.4	3.4	3.4	3.7	2.7	4.4	4.5	4.9	5.1
IFNA1	2.0	1.5	1.6	1.6	2.2	1.8	2.5	1.8	2.8	2.7	2.8	2.5	2.7	2.6	1.2	2.7	2.2	1.1	1.9	0.8	1.5	1.7	1.7	1.8	2.1	1.3	1.5	2.1	3.7	2.6	2.6	4.1
IFNB1	1.8	1.8	1.9	1.7	1.4	1.8	1.8	2.0	2.3	1.8	2.0	2.2	2.3	1.6	2.1	1.2	1.3	1.9	2.1	1.0	2.1	1.8	0.2	1.0	2.2	2.3	2.8	2.4	1.2	1.6	2.8	3.3
IFNG	2.5	2.1	2.3	2.2	1.0	1.8	2.3	1.5	2.8	2.9	2.2	2.7	2.1	2.2	1.7	1.7	1.4	0.5	1.5	1.5	2.0	1.1	2.4	2.0	1.8	2.0	2.4	1.7	2.3	2.6	2.8	1.4
IGFBP1	2.2	2.0	2.3	1.8	1.6	2.2	2.6	2.2	2.2	2.4	2.0	2.1	2.0	1.8	2.8	1.8	2.5	1.4	2.7	1.7	3.0	5.0	2.6	4.6	2.5	2.6	2.1	2.8	0.9	3.9	1.8	1.6
IGFBP2	5.8	5.5	5.8	5.5	5.7	5.5	5.8	5.5	5.8	5.9	5.8	6.1	6.2	6.4	5.9	6.4	7.2	9.9	7.3	9.7	7.0	6.2	7.0	6.0	7.1	8.1	7.3	8.0	6.7	7.2	6.9	6.9
IGFBP3	10.1	10.7	10.2	10.6	11.8	11.7	12.3	12.0	8.8	8.2	7.6	8.1	8.5	9.8	8.6	10.4	13.2	13.0	13.3	13.1	12.4	13.5	12.3	13.6	12.2	12.6	12.0	12.5	10.0	12.9	10.4	12.7
IGFBP4	10.0	11.4	9.9	11.4	9.9	10.8	10.2	10.6	8.0	8.3	7.9	8.4	9.2	9.7	9.4	10.1	9.9	12.0	10.1	12.1	10.6	11.7	10.5	11.8	9.8	11.9	9.6	11.9	10.6	12.2	10.6	11.8
IGFBP5	12.9	12.9	12.7	12.9	13.5	12.5	13.5	12.3	10.5	11.5	10.7	11.3	12.9	12.1	13.2	12.3	12.0	8.2	12.1	8.4	14.8	11.1	14.8	11.1	11.7	9.2	11.7	9.3	15.4	9.1	15.4	9.1
IGFBP6	8.5	8.6	8.5	8.6	7.8	7.2	7.8	7.1	8.1	7.1	7.8	7.3	7.4	7.2	7.4	7.1	9.2	9.9	9.3	9.8	9.0	8.1	9.1	8.2	8.9	7.9	9.1	8.0	8.4	8.7	8.3	8.4
IGFBP7	8.9	9.0	8.8	8.9	8.2	8.7	8.3	8.9	9.3	9.0	9.3	9.1	8.3	8.6	8.2	8.7	8.5	8.8	8.6	8.9	7.9	8.2	7.8	8.4	8.3	9.1	8.2	9.2	7.9	9.0	7.9	9.1
IHH	7.9	5.7	7.4	5.7	4.0	2.4	2.7	2.9	9.0	8.8	9.0	8.7	7.8	5.9	7.5	6.0	1.8	2.0	2.4	1.7	1.8	2.8	2.0	1.6	4.1	2.7	4.4	2.0	3.0	3.7	2.1	2.5
IL11	5.7	6.2	5.3	6.1	7.0	6.6	6.8	5.9	4.7	5.5	4.9	5.8	7.5	6.2	7.4	6.4	2.5	2.2	2.3	2.1	4.1	4.3	3.8	3.7	2.5	2.6	2.6	2.6	3.2	3.3	4.5	4.9
IL13RA1	8.8	7.6	8.8	7.6	8.1	7.2	8.2	7.1	9.2	8.1	9.0	8.4	7.7	7.1	7.6	7.1	8.3	6.5	8.5	6.3	9.6	6.7	9.5	6.5	9.0	6.2	8.8	6.4	9.6	7.1	9.5	7.2
IL16	6.6	7.5	6.4	7.3	5.1	6.8	5.3	6.7	7.3	7.1	7.0	7.2	5.4	6.0	5.5	6.0	4.3	4.3	4.6	4.6	3.1	6.4	2.9	6.4	5.7	5.6	5.3	5.9	4.3	6.5	3.7	6.3
IL1R1	8.3	8.4	8.2	8.3	7.3	6.9	7.4	7.0	7.6	8.2	7.5	8.4	8.3	7.9	8.1	8.0	7.7	7.6	7.7	7.5	7.1	7.0	7.0	6.9	7.9	8.6	7.8	8.6	7.5	7.3	7.6	6.8
IL20RB	5.8	3.5	5.2	3.5	2.3	1.3	2.1	2.3	7.7	6.5	7.0	6.5	4.2	2.4	4.2	2.7	2.7	2.3	3.2	2.0	1.5	2.5	2.5	2.3	4.2	3.2	2.8	3.2	1.8	3.4	3.3	2.1
IL6	2.8	3.3	2.6	3.0	3.5	2.9	3.4	2.9	3.7	3.0	3.0	3.0	3.7	3.8	3.5	3.5	3.1	2.6	2.9	2.6	3.8	2.9	3.0	3.3	2.9	2.9	3.3	3.6	4.0	4.4	4.0	4.7
IL7R	4.6	3.9	4.3	4.0	4.5	3.9	4.5	3.5	4.3	3.9	4.3	4.0	5.0	4.0	4.6	4.1	7.5	2.8	7.7	2.4	10.2	3.4	10.6	4.2	6.7	3.3	7.1	2.6	9.5	3.7	9.9	3.7
IRF6	5.9	4.0	5.5	3.5	3.0	3.1	3.7	3.8	8.1	7.1	8.0	7.3	4.1	3.5	4.0	3.7	3.5	2.4	3.0	2.1	4.3	3.0	3.9	4.1	4.0	2.4	4.2	2.6	4.2	2.8	3.7	4.7
IRF9	5.7	6.0	5.9	6.0	6.4	6.5	6.6	6.8	5.4	5.5	5.2	5.6	6.2	6.5	6.1	6.6	6.7	6.5	7.1	6.6	7.1	6.7	7.0	6.6	6.6	6.8	6.5	6.7	6.9	6.5	7.2	6.5
ITGA1	5.2	5.7	5.2	5.7	5.6	5.7	5.7	5.7	5.4	5.1	5.0	4.9	4.8	4.7	5.2	4.7	6.0	3.6	5.9	3.6	5.9	6.3	5.7	6.3	6.1	4.5	6.0	4.4	5.6	4.9	4.6	4.9
ITGA2	5.0	5.1	4.9	5.2	6.5	6.0	7.2	5.9	3.4	3.4	3.1	3.7	5.4	5.0	5.3	5.3	4.8	4.5	5.2	4.9	7.3	6.7	7.5	7.1	4.1	3.5	4.1	3.3	6.5	6.3	6.5	6.1
ITGA3	5.0	5.0	4.9	5.2	5.5	5.4	5.8	5.6	5.9	4.8	5.3	5.0	5.0	4.8	5.0	4.7	4.9	6.6	5.1	6.4	5.3	6.8	5.3	6.8	3.8	5.1	4.1	5.1	5.0	6.8	4.7	7.3
ITGA5	9.5	10.2	9.6	10.2	8.5	8.9	8.7	8.9	9.4	8.8	9.1	8.8	8.5	8.8	8.7	8.8	9.5	10.3	9.4	10.3	8.7	8.8	8.6	8.7	9.2	9.9	9.1	10.0	8.9	9.0	8.9	8.9
ITGB1	11.2	11.5	11.0	11.4	11.1	11.2	11.2	11.2	10.8	11.0	10.7	11.1	11.0	11.0	10.9	11.1	11.2	11.3	11.3	11.3	11.7	10.9	11.7	10.9	11.1	10.9	11.1	10.9	12.1	11.1	12.1	11.0
JAG1	6.7	6.1	6.6	6.4	8.7	7.9	8.7	8.1	5.8	5.8	5.7	5.8	7.5	7.7	7.5	7.6	6.3	5.3	6.4	5.4	8.7	5.1	8.6	5.5	6.4	6.0	6.3	6.1	8.6	5.3	8.7	5.2
JAG2	4.3	2.8	3.7	3.1	2.4	2.3	3.4	2.0	5.1	4.7	4.6	5.3	3.2	3.4	2.1	3.4	2.2	1.3	2.3	1.8	2.9	1.5	2.9	2.4	2.4	2.0	2.9	2.4	2.0	2.1	3.7	1.4
JUNB	8.8	8.2	8.9	8.2	7.9	7.7	7.9	7.5	8.7	8.8	8.8	9.0	8.5	8.3	8.5	8.2	7.6	5.9	7.9	6.1	7.8	7.4	7.4	7.2	7.7	5.8	7.9	6.3	7.4	7.9	7.1	8.2
KERA	3.1	2.8	2.8	2.6	2.8	2.9	3.1	2.8	4.2	3.3	3.9	3.7	2.8	2.8	3.4	1.7	1.7	1.0	1.2	1.7	1.4	2.0	2.4	2.2	2.5	1.2	2.4	1.6	2.1	2.2	2.8	2.8
KLF13	6.9	7.1	6.8	6.9	6.5	6.4	6.5	6.5	5.3	6.1	5.7	6.2	6.4	7.0	6.4	7.0	7.1	6.2	7.2	6.2	6.5	6.2	6.6	6.2	7.2	6.7	7.1	6.8	6.5	6.2	5.8	6.5
KLF15	4.4	3.4	4.4	3.2	2.2	2.5	3.1	1.9	6.0	4.8	5.4	4.8	2.5	2.5	2.7	2.1	4.5	4.6	4.8	4.7	3.4	3.1	3.2	2.6	6.0	6.4	5.6	6.4	1.6	3.8	2.8	3.7
KLF4	7.0	7.0	7.0	6.8	7.0	6.3	7.0	6.4	7.2	7.2	7.1	7.2	7.7	7.5	7.7	7.6	6.2	5.7	6.0	5.3	7.0	5.1	7.2	5.1	6.9	5.9	6.6	6.0	6.6	5.7	6.3	6.4
KLF6	6.8	7.8	6.6	7.7	6.7	7.1	6.7	7.0	6.6	6.7	6.2	6.8	6.5	7.2	6.5	7.2	8.7	9.3	8.8	9.2	7.9	7.2	7.9	7.3	8.2	9.3	8.2	9.4	8.3	7.6	7.8	8.0
LEF1	6.2	6.2	6.2	6.4	6.3	6.4	5.9	6.3	6.2	6.3	6.1	6.0	5.4	5.6	5.6	5.5	3.6	1.5	3.5	1.7	6.2	3.1	6.1	3.4	3.3	2.4	3.2	2.9	5.0	2.6	4.8	1.7
LGALS1	11.1	11.9	11.0	11.7	11.1	11.4	11.1	11.4	10.0	10.2	9.8	10.4	10.5	11.2	10.7	11.1	11.9	12.2	11.9	12.1	11.6	11.3	11.6	11.3	11.6	11.9	11.6	11.9	11.8	11.7	11.6	12.0
LGALS3	8.9	8.9	8.9	8.9	8.6	8.9	8.7	9.0	9.2	9.1	9.3	9.1	8.8	9.0	8.7	9.0	9.2	9.9	9.2	9.9	9.3	9.5	9.4	9.4	9.2	9.9	9.2	10.0	9.5	9.8	9.5	9.3
LGALS3BP	7.1	7.2	7.3	7.2	8.0	8.0	8.3	8.2	6.0	6.4	5.8	6.3	7.5	8.0	7.3	8.0	9.2	9.4	9.3	9.3	9.8	8.9	9.8	8.7	8.6	9.3	8.5	9.3	9.5	8.9	9.6	8.7
LPAR4	7.4	5.2	7.0	5.3	5.0	3.6	5.1	4.3	8.4	8.2	8.6	8.2	7.6	6.0	7.2	5.8	2.3	1.4	2.4	1.9	3.9	2.5	3.4	2.6	4.1	1.9	4.4	1.3	4.1	3.4	4.1	2.4
LRP5	5.4	5.6	5.1	5.5	5.5	5.5	5.8	5.7	4.9	5.7	4.9	5.9	5.7	6.0	6.0	5.7	5.4	5.0	4.9	4.8	5.8	6.4	5.9	6.7	5.3	5.8	5.8	5.3	5.9	6.5	6.0	6.7
LRP6	6.4	6.3	6.3	6.3	6.4	5.8	6.3	6.1	6.4	6.4	6.3	6.5	6.5	6.4	6.3	6.4	6.4	5.7	6.4	5.6	7.1	6.1	6.9	6.0	6.5	6.3	6.2	6.3	7.4	6.0	7.2	4.0
LUM	13.3	13.4	13.2	13.2	13.6	13.2	13.7	13.3	11.8	12.5	11.9	12.5	13.3	13.6	13.4	13.8	13.4	8.5	13.6	8.1	12.8	10.3	12.9	10.2	12.9	9.9	13.0	10.0	12.9	11.6	12.8	11.9
MAF	4.3	3.6	4.4	3.6	4.2	4.7	4.3	4.7	5.0	4.2	5.1	4.5	4.4	4.4	4.2	4.2	5.6	5.1	5.8	4.9	5.1	5.5	5.3	5.4	6.1	6.0	5.7	6.1	5.6	5.5	5.3	6.0
MAFB	6.4	6.0	6.4	6.0	6.8	6.3	6.8	6.5	5.3	5.9	5.4	5.8	6.8	7.1	6.7	6.6	6.1	5.8	6.0	5.8	7.0	6.5	7.1	6.4	6.6	6.9	6.3	6.8	7.2	7.3	7.1	7.4
MATN1	3.8	2.8	3.6	2.8	3.1	2.5	2.9	2.5	5.2	3.8	4.5	4.3	3.0	1.6	3.1	2.9	3.9	2.4	3.7	2.8	3.4	3.3	2.6	3.0	4.2	3.0	3.9	2.9	4.4	3.7	3.6	4.9
MATN2	6.7	7.3	6.5	7.3	6.9	7.4	6.7	7.5	6.2	7.4	6.0	7.4	7.3	8.1	7.2	7.8	5.2	4.2	5.3	4.0	5.4	5.6	5.2	6.0	6.3	5.9	6.2	5.9	6.1	5.9	6.3	6.3
MATN3	12.8	9.9	12.3	10.0	6.6	4.6	5.0	4.9	14.5	13.3	14.3	13.2	8.8	6.6	8.5	6.0	6.4	5.6	6.6	5.3	4.3	3.0	3.9	2.6	9.1	6.2	8.8	6.3	6.0	4.6	4.9	2.6
MATN4	8.2	6.5	8.0	6.8	5.3	3.4	4.7	2.5	9.4	9.5	9.4	9.5	8.1	6.3	7.7	5.5	2.9	0.9	3.3	1.5	3.4	1.0	3.0	1.6	4.8	1.9	4.2	2.4	4.5	2.3	3.6	1.4
MEF2C	8.4	7.5	8.2	7.3	7.8	6.0	7.7	6.3	8.6	8.6	8.6	8.7	9.1	8.1	9.0	8.0	6.5	4.8	6.8	4.9	7.5	5.3	7.5	5.2	7.9	5.4	7.7	5.6	8.2	5.6	8.0	6.0

MEF2D	5.9	6.3	6.2	6.2	6.6	6.3	6.4	6.4	6.4	5.9	6.3	6.0	6.1	6.4	6.0	6.5	5.9	5.5	5.7	5.4	6.1	5.7	6.2	6.2	6.1	5.7	5.7	6.0	6.0	6.0	6.0	5.6
MGP	10.4	9.6	10.3	9.8	11.4	10.1	11.5	9.8	9.4	9.7	9.5	9.6	10.8	10.1	11.1	10.1	9.1	9.3	9.1	9.3	10.2	8.3	10.1	8.4	9.4	10.9	9.2	11.1	10.0	6.7	10.1	6.7
MIA	8.3	6.7	8.0	6.9	6.0	4.5	5.6	4.2	9.2	8.8	9.1	9.0	7.3	5.8	7.4	6.0	5.0	2.3	5.2	2.3	5.4	4.2	5.5	4.3	6.4	3.5	6.1	3.4	5.4	4.1	5.9	5.0
MMP1	2.1	3.7	1.7	3.7	7.2	8.2	8.1	8.3	2.7	1.7	2.2	2.0	3.2	5.2	4.0	6.2	1.5	2.1	2.4	1.9	5.8	7.0	5.3	7.6	1.4	2.6	2.3	2.8	3.0	7.4	3.7	7.1
MMP13	9.3	10.8	9.4	11.1	12.8	13.7	13.3	13.8	6.6	6.5	6.7	6.5	10.5	11.9	10.7	12.5	9.8	3.3	9.8	3.0	11.2	11.2	11.3	11.0	9.2	4.7	8.9	4.5	10.3	11.1	9.9	11.0
MMP2	11.1	11.4	10.9	11.3	10.7	11.2	10.8	11.2	11.3	11.2	11.2	11.4	11.1	11.5	11.0	11.5	9.9	11.0	9.7	11.0	10.4	11.2	10.4	11.2	10.1	11.8	10.0	11.9	10.8	11.8	10.9	11.6
MMP28	6.0	5.4	6.2	5.7	4.3	4.5	4.2	4.4	6.5	6.0	6.4	6.2	4.3	4.8	4.8	4.8	7.2	6.7	7.1	6.7	4.3	3.3	4.0	3.0	7.9	7.9	7.8	8.1	3.8	4.4	4.1	4.6
MMP3	4.3	5.0	4.0	5.2	3.5	3.3	2.3	3.2	5.2	3.9	4.4	3.8	3.3	2.5	3.1	3.0	2.9	1.1	1.9	1.7	2.5	2.4	2.6	2.3	3.2	2.0	3.6	2.1	3.1	3.7	2.9	3.5
MMP7	5.3	6.6	5.9	6.5	2.8	2.4	2.0	2.4	6.3	6.9	6.0	7.0	3.1	2.6	2.1	1.5	8.8	8.0	8.7	7.9	4.1	1.8	3.6	2.9	8.5	9.9	8.3	10.0	3.6	3.2	4.3	2.7
MMP8	3.4	3.0	3.3	2.7	3.2	2.9	3.7	2.0	3.6	3.6	3.0	3.5	3.5	3.6	3.2	3.1	4.4	2.4	3.6	2.1	3.2	1.0	3.7	2.6	3.8	2.5	3.8	2.2	4.3	3.7	3.9	3.3
MMP9	3.7	3.5	3.4	3.5	4.6	3.2	4.9	3.5	3.2	3.6	3.3	3.5	4.5	5.0	4.5	4.7	2.9	1.7	3.4	2.0	4.0	3.3	4.6	3.8	3.7	2.8	3.4	3.0	4.5	5.4	5.2	4.6
MST1	4.2	3.6	4.0	3.8	4.3	3.7	3.8	3.3	4.4	3.7	3.6	3.8	3.6	3.8	3.4	4.0	5.3	4.7	5.3	4.5	5.5	3.4	5.7	4.2	5.3	5.1	5.6	5.7	5.6	4.5	5.8	4.5
MST4	7.5	5.8	7.3	5.6	5.4	4.5	5.2	4.5	8.7	8.1	8.9	8.2	6.8	5.2	6.5	4.9	4.1	3.6	4.1	3.5	4.8	3.8	4.8	3.5	5.4	3.9	5.5	4.3	5.2	4.7	5.1	4.6
MSX1	6.9	5.7	6.7	6.0	6.5	4.8	6.8	5.3	5.1	4.9	5.2	5.2	6.0	5.4	5.7	5.6	7.7	3.4	8.1	3.6	8.4	4.1	8.5	4.0	6.4	4.3	6.6	4.1	8.1	4.8	8.1	4.9
MYC	7.5	7.8	7.4	7.7	5.6	5.6	5.1	5.6	8.1	8.0	8.1	8.0	6.5	6.6	6.6	6.3	6.5	7.9	6.4	8.0	4.9	5.5	5.0	5.5	7.0	7.7	7.1	7.8	5.4	5.8	4.8	5.9
NFAT5	7.3	6.8	7.2	6.8	7.2	7.0	7.4	7.0	7.3	7.4	7.4	7.4	7.5	7.2	7.3	7.0	6.7	6.5	6.9	6.7	7.5	6.8	7.3	7.0	6.8	6.8	6.8	6.9	7.6	6.8	7.2	7.0
NFE2	1.4	0.9	0.4	1.1	0.9	1.2	1.8	0.9	1.7	1.2	1.4	1.7	1.4	0.7	1.1	1.5	0.2	0.8	0.9	1.0	1.1	1.6	1.5	0.2	2.1	1.5	1.0	1.4	2.7	0.9	1.5	2.1
NFIB	3.5	3.5	3.8	3.9	2.9	3.2	3.5	3.5	3.7	3.7	3.5	3.3	3.0	3.3	3.6	3.5	6.6	6.5	6.9	6.6	6.0	5.8	5.7	5.8	6.2	7.2	6.0	7.0	5.4	6.6	5.4	6.0
NFIL3	6.3	6.3	6.6	6.2	4.9	5.2	5.3	5.0	6.9	6.4	6.6	6.5	5.5	5.4	5.3	5.2	7.1	8.0	6.9	8.0	6.5	6.3	6.7	5.8	7.2	8.7	7.0	8.9	6.9	6.4	6.8	6.4
NFKBIA	7.7	7.2	7.7	7.2	6.3	5.8	6.6	5.6	6.9	6.6	6.7	6.8	6.5	6.3	6.4	6.3	8.9	7.6	8.9	7.7	7.3	6.0	7.3	6.0	9.0	8.5	8.9	8.4	7.8	6.8	7.8	6.7
NGEF	6.9	6.3	6.9	6.5	6.5	5.8	6.5	5.7	7.4	7.6	7.3	7.7	7.3	7.0	7.1	6.9	4.0	2.6	4.0	2.4	4.7	5.1	4.7	4.8	5.7	2.4	5.7	2.8	3.9	5.0	4.8	5.0
NOG	5.9	3.1	6.0	2.8	2.6	2.2	2.7	0.9	7.0	4.1	7.0	4.3	3.9	1.6	4.0	2.7	6.8	2.1	6.4	1.6	6.7	2.8	6.8	3.3	6.9	1.4	6.9	2.6	8.1	2.7	8.4	2.6
NOS2	5.9	4.3	5.5	4.2	3.3	1.9	2.9	1.9	6.3	6.6	6.7	6.5	4.5	3.2	4.4	3.4	3.2	1.6	3.1	1.9	2.0	2.6	3.2	2.3	3.6	3.0	4.0	2.7	4.0	3.6	3.7	4.0
NOTCH1	3.7	4.2	3.9	4.2	5.1	5.5	5.3	5.6	3.5	3.9	3.5	3.9	4.5	5.0	3.2	5.1	4.3	3.5	4.6	3.8	5.2	5.2	5.2	5.3	4.7	4.2	4.4	4.1	3.2	5.5	4.9	5.5
NOTCH2	9.2	9.3	9.2	9.4	9.0	8.7	9.0	8.6	8.3	8.7	8.1	8.7	8.9	8.8	9.2	8.8	9.9	8.6	9.8	8.6	9.7	8.4	9.8	8.7	9.6	9.0	9.5	9.0	10.3	8.8	10.2	9.1
NOTCH3	8.7	8.7	8.7	8.7	9.6	8.8	9.7	8.9	6.7	6.9	6.3	6.7	8.5	8.6	8.8	8.7	9.0	7.4	8.8	7.5	9.6	7.8	9.4	7.9	8.2	7.3	8.2	7.5	9.5	7.9	9.5	7.3
NOX1	2.6	2.1	2.2	2.1	3.1	2.2	2.7	2.9	2.7	2.2	2.4	2.1	2.5	1.9	2.0	2.7	2.8	1.5	2.9	2.0	3.4	3.0	2.6	3.2	2.8	3.1	3.1	1.9	3.7	2.9	3.1	4.8
NPAS2	5.7	6.1	5.4	5.9	3.5	3.8	3.3	3.5	7.0	6.4	6.7	6.5	3.7	4.1	2.5	4.0	4.8	6.4	4.9	6.1	3.5	4.8	3.7	4.5	5.4	6.2	5.5	6.4	4.1	5.3	4.1	5.3
NR4A2	4.4	4.0	4.2	3.9	5.0	4.3	4.9	4.0	4.0	4.4	3.9	4.3	5.2	4.8	4.5	4.1	3.7	2.5	3.7	2.3	4.6	4.8	4.9	5.4	4.3	3.6	3.8	2.9	4.4	4.0	4.6	3.5
NRG1	4.4	4.2	4.0	4.1	4.4	4.4	4.8	4.4	4.8	4.5	4.3	4.5	4.3	4.4	4.3	4.2	3.5	3.8	3.3	3.4	5.1	4.7	6.0	4.3	3.8	4.7	4.2	4.3	5.0	3.8	5.3	3.2
OAF	8.5	6.3	8.3	6.5	6.2	5.2	5.9	5.2	9.1	8.4	9.2	8.3	7.8	6.4	7.7	6.1	6.5	4.6	6.1	4.5	6.4	5.8	6.3	6.0	7.5	4.4	7.3	4.9	5.6	6.3	6.7	5.7
OGN	8.9	8.8	8.7	8.4	7.1	6.9	7.2	7.2	7.9	8.7	7.7	8.8	8.6	9.1	8.4	8.9	9.0	6.1	9.2	5.8	7.6	3.0	8.2	3.2	10.0	7.5	10.1	7.5	8.9	4.9	8.5	5.6
OMD	11.0	10.4	10.9	10.4	10.1	7.8	10.0	8.0	9.4	10.3	9.2	10.6	11.7	10.3	11.4	10.2	10.3	5.4	10.6	5.1	10.2	2.4	10.4	2.4	11.6	7.1	11.6	6.9	11.6	5.2	11.4	4.8
PANX3	9.8	8.1	9.3	7.6	4.3	3.6	4.0	3.4	9.6	11.3	10.0	11.4	9.3	8.1	8.5	6.9	3.3	1.7	4.1	1.3	2.7	1.5	2.4	2.4	8.1	1.8	8.0	3.3	3.1	1.7	3.9	3.9
PBX1	5.1	5.0	5.0	4.9	6.1	5.9	6.1	5.8	3.6	4.3	3.7	4.3	5.8	5.9	5.3	5.8	6.6	6.2	6.7	6.2	7.5	5.7	7.8	6.0	6.3	6.8	6.1	6.7	8.1	6.1	8.0	6.0
PCOLCE2	9.0	7.4	8.7	7.5	6.9	6.2	6.6	5.5	9.7	8.7	9.7	8.8	7.7	6.7	7.5	6.4	7.3	6.2	7.2	6.0	7.9	4.5	8.3	3.6	8.4	5.6	8.4	5.1	8.8	4.8	8.8	4.5
PDPN	7.0	6.6	6.5	6.5	5.9	7.6	6.1	7.2	8.7	7.1	8.8	7.2	6.3	6.5	6.4	7.1	3.5	7.5	3.7	7.7	3.9	8.3	3.7	8.2	5.5	7.9	5.6	8.0	4.1	8.6	4.7	8.3
PGK1	12.1	12.0	11.8	11.6	9.8	9.6	9.5	9.4	12.5	12.0	12.5	12.3	10.3	9.8	10.2	9.7	9.8	9.5	10.0	9.4	9.4	8.8	9.4	8.9	10.3	8.9	10.0	8.9	9.6	9.2	9.4	9.2
PLCD1	9.6	8.6	9.4	8.5	7.7	6.8	7.5	6.9	10.5	10.1	10.3	10.2	9.4	8.7	9.2	8.6	8.0	7.5	8.0	7.5	7.1	6.4	7.3	6.6	8.8	7.7	8.7	7.8	7.6	7.0	7.5	7.5
PLCG2	2.8	2.0	2.8	2.1	2.3	3.5	3.1	3.2	3.0	3.0	2.8	3.1	3.5	2.0	1.2	3.0	2.5	1.3	2.0	1.5	3.2	2.8	3.2	3.4	2.9	1.2	2.6	2.6	3.7	2.7	3.1	2.6
PMP2	5.2	3.0	4.9	3.3	2.4	1.4	2.8	1.9	6.3	5.3	6.3	5.5	3.6	2.8	3.6	2.9	2.8	1.9	2.8	1.7	2.2	1.6	2.9	2.9	3.2	2.4	2.7	2.3	2.2	3.5	3.2	4.0
PPARG	3.2	3.0	3.4	2.9	1.2	2.5	1.8	2.2	4.1	3.6	3.9	3.5	2.7	2.0	1.9	1.8	6.5	7.3	6.7	7.2	3.3	3.5	3.8	3.7	7.2	7.3	7.1	7.4	4.0	4.3	4.1	4.0
PPP1R14C	5.6	5.1	5.4	4.9	4.5	4.1	4.6	4.2	6.5	5.6	6.1	5.6	5.4	3.1	4.8	3.9	2.0	2.6	2.5	2.1	3.0	3.2	3.3	3.1	3.2	1.6	2.8	1.6	3.1	2.6	3.1	3.0
PRELP	7.5	6.8	7.2	6.8	4.2	4.1	3.7	3.2	7.3	7.3	7.5	7.4	6.0	4.8	5.3	4.3	2.7	2.1	2.5	1.7	2.6	2.6	2.5	0.9	4.6	3.1	4.8	2.6	2.7	3.1	3.6	2.2
PRG4	5.2	5.4	5.2	5.5	5.5	6.0	5.7	5.6	5.9	5.0	5.6	5.3	4.9	4.7	5.1	5.1	5.0	7.0	4.2	6.7	3.1	4.0	2.7	3.2	5.0	9.3	5.2	9.3	3.8	3.6	2.9	4.4
PSMC4	7.4	7.1	7.1	7.1	7.0	7.1	7.0	7.1	6.8	7.3	7.1	7.3	7.3	7.4	7.4	7.3	6.7	6.3	7.0	6.5	7.0	6.8	7.0	6.8	6.8	6.3	7.1	6.2	7.3	6.9	7.1	7.0
PTCH1	7.2	6.5	6.8	6.3	5.7	5.0	5.7	5.0	7.2	7.9	7.2	8.1	7.3	6.5	6.9	6.8	5.4	4.4	5.3	4.3	6.1	3.4	6.3	2.4	6.2	5.2	5.7	5.1	6.3	3.7	6.5	3.1
PTCH2	2.5	2.5	2.2	2.4	3.3	2.7	3.6	2.5	2.6	2.6	0.8	2.6	3.4	3.5	2.1	2.9	2.8	2.3	2.7	2.7	3.7	2.6	3.5	2.5	2.9	2.9	2.7	3.1	3.4	3.3	3.4	2.2
PTHLH	2.7	2.0	2.9	2.2	2.9	2.7	3.4	3.0	4.0	2.7	3.3	3.2	3.4	2.7	2.8	3.0	2.2	1.1	2.4	1.8	4.9	5.1	5.2	5.7	1.6	1.9	1.8	0.9	5.0	4.2	5.4	3.3
PTHR1	9.4	8.3	9.0	8.1	6.3	4.9	5.9	5.2	10.3	10.2	10.3	10.4	8.8	8.6	8.8	8.2	7.1	4.0	7.3	3.8	3.9	2.7	4.0	2.7	8.5	5.5	8.4	5.7	5.4	2.4	5.1	4.6
RASL11B	8.3	9.2	8.3	9.0	8.0	7.2	8.1	7.4	5.7	7.5	5.7	7.8	7.4	8.0	7.3	7.9	5.4	4.7	5.6	4.7	6.8	2.7	6.5	2.3	5.6	6.3	5.5	6.4	6.8	2.4	6.8	4.5
RCOR2	7.4	6.5	7.1	6.2	4.9	4.1	5.0	3.9	7.6	7.8	7.6	7.9	7.2	6.3	7.1	6.0	4.2	2.7	4.1	3.2	4.5	2.5	4.1	3.1	5.9	3.3	6.0	3.2	3.7	3.4	4.1	4.8
RNF144	7.4	7.8	7.4	7.5	5.9	6.3	5.7	6.3	7.0	7.2	6.8	7.4	6.5	6.8	6.4	6.5	5.3	5.7	5.6	5.8	4.4	6.2	4.4	5.9	6.3	6.7	5.8	6.5	5.2	6.2	4.6	6.4
RPL37A	13.8	13.7	13.8	13.6	13.1	13.0	13.2	13.1	14.0	13.8	13.9	14.0	13.6	13.5	13.4	13.4	14.3	14.2	14.4	14.2	13.9	12.7	13.9	12.7	14.4	14.5	14.3	14.5	14.0	13.4	14.0	13.3
RPS27L	9.9	9.9	10.0	9.9	10.2	10.4	10.2	10.4	9.9	9.9	9.9	10.0	10.1	10.2	10.0	10.3	9.8	10.3	9.8	10.3	10.0	10.2	10.2	10.2	10.0	10.2	9.9	10.3	10.1	10.4	10.1	10.2
RUNX1	8.5	8.9	8.3	8.9	9.2	9.4	9.3	9.4	7.7	8.2	7.7	8.3	8.9	9.5	8.9	9.4	7.8	6.9	7.7	6.9	8.4	8.1	8.1	8.2	7.6	7.6	7.2	7.7	8.7	8.4	8.6	8.4
RUNX2	8.0	8.2	7.9	8.1	8.5	8.2	8.7	8.2	7.1	7.3	7.1	7.5	8.6	8.8	8.6	8.8	8.5	6.5	8.5	6.4	8.7	7.4	8.7	7.4	8.5	7.3	8.4	7.3	8.8	7.5	8.8	8.0
RUNX3	6.2	5.9	6.2	5.8	5.4	5.0	5.5	4.6	6.6	6.4	6.2	6.3	5.9	5.1	5.7	5.1	5.2	4.2	5.2	4.3	5.3	3.3	5.6	3.5	5.0	4.9	5.4	5.0	5.6	4.7	5.5	3.8
RYK	7.2	6.9	7.3	6.9	7.4	7.2	7.4	7.3	6.8	7.0	7.0	7.1	7.2	7.2	7.0	7.2	8.1	7.1	8.2	7.1	7.6	7.0	7.6	7.2	7.8	7.4	7.7	7.3	7.7	7.4	7.6	7.1
S100A1	10.0	7.9	9.7	8.1	7.1	4.8	6.5	4.6	10.8	10.4	10.9	10.5	9.0	7.2	8.9	6.9	3.9	1.8	3.8	1.4	3.9	1.5	3.8	1.8	6.3	2.0	6.3	3.2	3.6	1.7	4.4	1.6
S100B	7.1	5.3	6.9	5.6	5.2	4.1	4.6	4.5	7.2	7.0	7.2	6.9	6.0	5.3	5.9	4.8	6.5	3.9	6.3	3.4	5.4	3.4	5.4	2.7	7.6	4.1	7.6	4.6	5.4	4.2	5.8	1.9
S100P	11.5	7.1	11.0	7.2	5.7	2.9	4.1	2.2	13.3	10.6	13.2	11.2	8.7	4.9	8.1	4.7	4.8	1.7	5.4	2.0	4.4	2.9	3.7	2.0	8.6	3.2	8.4	2.9	5.8	3.5	6.0	3.3
SCIN	9.0	6.6	8.7	6.5	5.4	3.3	5.0	2.8	10.1	9.4	10.1	9.7	7.4	4.7	7.2	4.8	5.5	2.6	5.4	2.8	5.0	2.8	5.5	3.9	7.0	3.5	6.6	3.1	5.0	3.8	5.3	2.8
SCRG1	11.2	9.5	11.1	9.5	6.8	5.0	6.8	4.8	11.9	11.2	11.8	11.4	9.1	7.5	8.7	7.0	11.2	8.3	11.3	8.0	7.6	3.4	7.9	1.7	11.4	9.7	11.2	9.8	7.9	2.9	8.0	2.7
SDC3	6.9	6.2	6.9	6.3	6.1	5.2	5.8	4.8	6.8	6.7	6.8	6.8	6.6	5.9	6.6	5.6	8.8	7.0	8.9	7.0	7.6	6.1	7.8	6.1	8.5	7.1	8.5	7.2	8.2	6.1	8.0	6.5
SERPINA1	4.0	3.5	4.0	3.6	3.4	4.6	4.2	4.7	5.6	4.0	5.2	4.6	4.7	3.6	3.9	3.3	2.6	2.6	3.3	2.8	3.0	2.3	3.4	3.0	3.1	2.1	3.1	2.2	0.9	3.5	1.7	1.1
SERPINA3	7.8	7.1	7.9	7.4	4.8	4.1	4.5	3.2	9.2	8.7	9.0	8.7	6.3	4.8	6.1	4.8	5.1	5.7	5.0	5.4	4.1	2.4	4.0	2.9	6.1	6.0	6.3	6.4	4.4	2.0	4.6	3.3
SERPINA5	5.0	4.7	5.2	4.8	3.5	3.0	4.2	3.8	5.4	5.3	5.2	5.4	4.0	3.9	4.2	4.2	4.1	3.5	3.9	3.2	4.2	2.0	3.9	1.5	4.3	4.3	4.1	4.0	3.8	3.2	4.0	2.9
SFRP1	5.3	4.1	4.9	3.7	1.2	3.5	2.4	2.7	6.8	5.7	6.2	5.7	3.6	3.0	2.2	2.8	3.4	4.2	3.4	4.1	3.8	5.0	2.0	5.5	4.3	5.1	4.0	4.9	3.7	4.0	3.4	4.4
SFRP2	2.6	3.0	2.5	2.5	2.7	2.9	2.4	2.1	3.1	2.7	2.8	2.5	2.7	2.4	1.4	2.3	2.4	1.9	2.7	1.6	2.5	1.5	2.1	2.9	1.4	2.1	2.3	2.4	1.4	1.4	3.2	1.8
SFRP4	3.7	2.1	3.6	2.4	9.3	6.9	9.8	7.4	2.2	2.6	2.6	2.8	8.4	7.5	8.4	7.8	2.2	1.9	1.7	2.2	7.9	6.8	7.9	6.6	1.9	2.4	3.3	3.6	4.9	6.6	5.6	5.9
SHC4	7.8	6.4	7.5	6.5	6.0	4.1	5.6	4.1	8.7	8.4	8.8	8.4	6.4	5.0	6.5	4.9	4.9	3.4	5.0	3.3	5.0	4.2	5.0	3.1	5.8	3.5	5.7	4.0	5.4	3.5	5.0	2.8
SLAMF9	4.7	3.4	4.6	3.5	2.6	1.8	2.3	1.5	6.5	4.1	5.8	4.7	2.9	2.6	3.3	1.2	1.8	1.1	1.4	1.4	2.2	1.9	3.1	2.1	2.5	1.9	2.4	1.0	3.4	1.7	2.6	1.8
SLC13A5	7.5	5.1	7.0	5.0	2.3	1.5	2.4	2.2	8.6	7.6	8.6	8.0	6.4	4.5	6.4	4.4	1.8	1.2	2.6	1.6	3.3	1.9	1.9	1.4	5.0	1.8	4.5	2.1	3.1	3.5	4.0	2.6
SMAD1	5.8	6.2	5.8	6.4	5.7	6.4	5.7	6.4	5.3	5.9	5.5	5.8	5.9	6.5	6.0	6.5	6.4	5.6	6.1	5.7	6.3	6.7	6.6	6.8	6.3	5.6	6.3	5.9	6.8	7.0	6.7	7.5
SMAD2	7.8	8.1	7.9	8.1	8.1	8.2	8.1	8.0	7.7	7.8	7.6	7.9	8.1	8.2	8.3	8.3	8.4	8.1	8.4	8.0	8.6	7.9	8.6	7.6	8.4	8.5	8.4	8.4	9.0	8.5	8.7	8.5
SMAD3	5.4	5.6	5.3	5.5	5.6	5.4	5.9	5.4	5.8	5.8	5.8	5.9	6.1	5.8	5.8	5.6	5.8	6.2	5.9	6.2	6.6	6.5	6.8	6.2	6.3	7.0	6.0	7.1	6.6	6.7	6.5	6.6
SMAD4	6.8	6.8	6.8	6.9	6.8	6.7	6.9	6.9	6.3	6.6	6.3	6.7	6.7	7.0	7.0	7.1	7.3	6.9	7.4	7.0	7.2	6.5	7.3	6.4	7.0	7.2	7.2	7.2	7.4	6.7	7.4	6.9
SMAD5	8.0	7.8	7.9	7.9	8.0	7.8	8.1	7.7	7.6	7.8	7.6	8.0	7.8	7.8	7.7	7.9	8.1	7.6	8.1	7.6	8.7	7.3	8.4	7.5	8.1	8.0	8.1	8.2	8.7	7.8	8.8	7.9
SMAD6	8.7	6.8	8.6	6.8	8.6	6.7	8.6	6.6	7.8	6.9	8.0	7.2	8.7	7.6	8.9	7.6	9.3	4.2	9.2	4.1	9.9	4.4	9.9	5.0	9.4	4.9	9.4	5.0	9.9	4.8	10.0	5.6
SMAD7	8.8	8.2	8.8	8.3	9.4	8.9	9.5	8.8	7.2	7.0	7.5	7.3	9.0	8.7	9.1	8.8	8.8	5.0	8.8	5.0	9.4	7.1	9.4	7.1	8.7	5.5	8.7	5.6	9.8	7.1	9.7	7.7
SMAD9	8.1	6.3	8.0	6.5	7.1	5.0	7.2	5.2	7.5	6.0	7.2	6.3	6.5	5.4	6.7	5.2	8.2	3.8	8.3	3.9	8.3	4.5	8.3	4.6	7.9	4.4	7.7	4.7	8.5	4.3	8.5	3.5
SMO	5.8	5.5	5.6	5.3	4.9	4.9	4.9	5.4	5.5	5.8	5.6	5.8	5.4	5.8	5.1	5.6	5.5	4.7	5.8	4.8	6.1	5.2	5.9	4.8	6.1	5.0	5.7	5.1	6.4	5.2	6.5	5.2
SOCS2	7.1	5.8	7.1	5.9	6.1	3.8	6.2	3.8	7.6	5.8	7.2	5.9	6.5	4.7	6.6	4.6	5.8	4.8	5.2	5.0	6.8	4.2	6.7	4.2	6.1	4.0	5.9	4.0	6.9	4.5	7.0	4.7
SOX11	4.9	5.4	5.0	5.4	5.5	6.2	5.7	6.0	4.2	4.1	4.0	3.7	4.9	5.2	5.1	5.9	4.6	4.6	4.6	4.7	4.6	6.2	4.3	6.3	5.0	4.9	4.6	4.7	4.3	6.0	5.4	6.3
SOX5	7.2	6.3	7.1	6.3	7.4	6.8	7.6	6.7	7.5	7.3	7.6	7.3	7.2	6.5	6.8	6.7	5.5	5.3	5.8	5.1	5.8	6.2	5.8	6.5	5.2	5.6	5.4	5.4	5.1	5.9	5.7	5.9
SOX6	5.9	5.3	5.7	5.2	4.9	4.4	4.9	4.0	6.6	6.4	6.4	6.6	5.7	5.4	6.1	4.8	3.6	2.8	3.3	2.7	3.8	2.8	4.2	3.5	4.7	3.2	5.1	2.6	4.1	3.6	3.9	2.5
SOX8	8.3	5.6	8.0	5.5	3.8	1.8	3.1	2.4	9.3	8.6	9.3	8.8	7.0	4.9	6.7	4.3	4.9	2.0	4.8	2.1	3.4	2.4	3.2	1.2	6.7	2.6	6.8	2.5	5.2	1.3	5.0	4.0
SOX9	10.9	9.9	10.7	9.9	9.1	8.4	9.1	8.4	12.0	11.2	11.8	11.3	9.7	9.2	9.6	9.1	9.8	7.4	9.8	7.4	9.6	8.3	9.6	8.4	10.4	7.9	10.2	8.1	10.3	7.9	10.2	7.9
SP7	8.9	8.3	8.9	8.0	8.3	6.7	8.3	6.7	8.2	8.5	8.4	8.6	9.3	8.6	9.4	8.4	8.5	1.8	8.6	2.0	8.7	2.9	8.8	2.9	8.8	3.3	9.1	3.0	9.3	3.7	9.3	4.1
SPARCL1	4.6	4.1	4.4	4.0	1.9	2.5	2.2	1.4	4.7	4.6	4.3	4.2	1.8	2.6	1.8	2.3	8.3	11.0	8.0	11.0	2.1	2.8	2.2	2.3	7.6	11.2	7.3	11.3	2.0	4.2	3.4	4.1
SPP1	14.3	12.4	14.0	12.2	15.5	13.9	15.8	14.3	9.3	11.6	9.4	11.7	15.4	14.4	15.1	14.3	12.7	5.0	12.9	5.0	14.7	12.3	14.7	12.3	11.6	5.2	11.6	5.8	12.4	8.6	12.4	9.2
STEAP3	7.1	7.3	7.1	7.3	7.0	7.4	7.0	7.2	7.0	8.1	7.1	8.2	7.9	8.3	7.7	7.9	6.5	6.8	6.4	6.9	5.6	6.7	6.2	6.6	6.3	6.2	6.5	6.2	5.4	6.9	5.3	6.7
TBP	5.6	5.5	5.4	5.4	5.3	5.2	5.2	5.1	5.1	5.5	5.3	5.3	5.8	5.4	5.5	5.3	5.8	5.0	5.6	5.2	5.4	5.1	5.8	4.6	5.6	5.5	5.7	5.9	5.8	5.3	5.6	5.0
TBX15	5.5	5.4	5.5	5.7	5.1	5.2	5.4	5.2	5.7	5.2	5.1	5.3	4.1	4.4	4.8	4.7	7.2	6.5	7.1	6.6	6.1	5.4	5.7	5.4	7.0	6.8	6.8	7.1	6.3	5.7	6.4	5.9
TBX2	7.3	6.7	7.2	6.5	6.4	5.9	6.4	6.0	5.2	5.4	5.4	5.5	6.1	5.9	6.0	5.7	8.7	5.4	8.8	5.4	9.2	6.9	9.4	6.9	8.2	5.7	8.4	5.6	8.6	6.3	8.5	6.6
TBX3	7.1	6.5	6.7	6.6	6.8	6.4	7.1	6.5	4.4	4.6	4.4	4.2	5.8	5.4	5.8	5.8	7.9	6.9	7.9	6.8	8.6	5.8	8.7	5.9	7.8	6.5	7.6	6.7	9.4	6.3	9.5	5.8
TGFB1	10.7	10.4	10.6	10.3	11.4	10.5	11.6	10.5	9.0	10.1	9.0	10.1	11.4	11.1	11.3	11.0	10.4	8.6	10.5	8.6	11.4	9.2	11.2	9.2	10.0	9.0	10.1	9.1	10.9	9.1	10.8	9.4
TGFB2	7.9	7.6	8.1	7.9	9.7	9.3	9.9	9.2	7.8	8.0	7.5	8.2	9.1	8.7	9.3	8.9	8.4	6.2	8.4	6.3	8.9	9.9	8.7	9.9	8.9	7.4	8.7	7.4	7.9	9.0	8.1	9.2
TGFB3	4.4	5.9	4.6	5.8	6.1	6.3	6.1	6.5	2.8	4.5	3.5	4.1	5.6	6.6	5.6	6.8	3.7	4.3	4.5	4.3	5.7	6.4	5.8	6.6	4.0	4.3	4.1	4.4	6.7	6.6	6.6	6.7
TGFBR1	8.1	8.2	8.0	8.3	8.0	8.1	8.3	8.1	7.6	7.8	7.6	7.8	7.6	7.9	7.8	7.7	7.0	6.1	7.1	6.0	7.0	7.0	7.2	7.2	6.7	6.3	6.5	6.1	7.4	7.5	7.4	7.1
TGFBR2	6.2	6.4	6.3	6.5	5.9	5.9	6.1	5.8	5.9	6.0	5.7	6.1	5.9	6.0	5.7	5.5	6.7	8.0	6.6	8.0	6.7	7.2	7.0	7.2	6.3	7.1	6.3	7.2	6.6	7.1	6.5	6.5
TGFBR3	3.1	3.8	2.9	3.8	3.4	3.3	2.5	3.7	2.2	2.6	2.3	2.7	2.4	3.2	2.5	3.3	5.1	7.1	4.6	7.3	4.4	5.1	3.9	4.7	5.1	8.4	5.0	8.5	4.3	5.3	4.5	5.5
TIMP2	8.7	9.4	8.7	9.3	9.6	10.2	9.8	10.2	7.1	7.9	7.1	8.0	9.1	9.8	9.3	10.0	9.1	10.0	9.0	9.9	9.3	10.3	9.2	10.3	9.0	10.5	9.0	10.5	9.3	10.4	9.3	10.6
TIMP4	8.4	7.1	8.1	7.1	4.5	4.3	4.5	4.6	9.6	8.3	9.4	8.5	5.2	5.0	4.7	4.6	10.3	10.0	10.2	10.0	5.4	4.9	5.2	4.7	10.7	10.4	10.6	10.5	6.1	5.4	5.4	5.0
TMEM100	5.6	4.9	5.3	5.1	5.2	4.6	5.5	4.3	5.5	5.4	5.4	5.4	4.2	3.4	3.4	3.7	7.3	7.1	7.2	7.4	8.3	4.3	8.6	4.4	6.9	7.8	6.7	8.0	6.5	4.8	6.3	4.1
TNFAIP8L3	5.1	4.4	5.0	4.2	3.0	1.7	3.4	2.2	5.5	4.7	5.4	4.8	3.3	3.3	3.8	2.5	4.1	4.8	4.4	4.7	3.2	2.7	2.8	2.3	4.6	4.1	4.7	4.7	2.9	1.7	3.7	1.3
TNFRS11B	2.8	3.2	2.5	2.8	3.2	3.5	2.8	2.7	3.7	4.4	3.8	4.1	4.2	3.5	4.1	4.1	2.3	3.7	2.3	3.9	3.2	4.6	2.5	5.0	2.9	4.2	2.5	4.7	3.7	4.3	1.8	5.1
TNFRSF11A	2.0	2.2	2.2	1.9	1.4	1.9	2.3	1.2	2.4	2.1	2.4	2.1	2.1	1.8	2.9	2.0	1.6	1.5	1.8	1.4	1.9	1.2	1.7	1.6	2.0	2.4	1.7	1.1	2.7	2.0	3.1	2.0
TNFSF11	7.6	7.0	7.4	7.1	9.2	8.8	9.3	8.4	6.7	7.2	6.9	7.1	8.2	7.9	8.7	8.1	3.2	3.2	3.5	3.4	5.7	6.2	6.0	6.4	2.8	3.7	3.2	3.6	6.7	5.0	6.4	6.1
TRPV4	8.2	6.2	7.9	6.1	5.6	4.6	5.0	4.3	9.3	8.7	9.4	8.8	7.3	5.9	6.9	5.4	5.6	2.8	5.5	2.8	4.9	3.7	5.2	3.2	6.7	2.7	6.0	3.2	6.3	4.2	6.1	2.1
TSC22D1	6.4	6.6	6.2	6.5	6.8	7.3	6.8	7.1	6.0	6.3	5.6	6.3	6.6	6.9	6.6	7.1	7.3	7.4	7.4	7.2	7.2	6.8	7.3	6.8	7.3	7.5	7.1	7.4	7.4	7.0	7.6	7.0
TSC22D3	10.5	10.6	10.4	10.4	7.3	7.0	7.2	7.2	10.3	9.7	9.9	9.8	7.5	7.9	7.3	7.8	10.8	10.3	10.8	10.4	7.3	5.6	7.4	6.0	10.9	11.4	10.8	11.5	8.1	6.6	8.0	6.2
TSC22D4	8.5	7.6	8.5	7.4	7.4	6.9	7.5	7.1	9.4	8.5	9.2	8.7	7.7	7.3	7.8	7.4	8.6	8.0	8.5	8.0	8.0	7.2	8.1	7.2	8.8	8.1	8.8	8.4	8.2	7.3	8.1	7.7
TUBB2A	9.5	7.7	9.3	7.7	6.6	6.7	6.4	6.8	10.7	10.1	10.9	10.0	8.5	7.6	8.4	7.4	8.0	7.0	8.0	6.9	8.1	6.0	7.9	6.0	8.3	6.9	8.3	6.8	8.8	6.8	8.8	7.0
TUBB2B	9.3	6.5	8.8	6.5	5.3	4.1	4.7	4.5	10.9	9.8	10.8	9.8	7.4	5.5	6.6	5.3	6.7	4.8	6.6	5.0	7.9	3.9	7.9	4.1	7.4	6.2	7.3	6.2	8.7	5.2	8.7	5.4
TXNIP	10.0	9.6	10.2	9.4	8.0	7.8	7.7	7.7	9.3	9.2	9.0	9.6	8.0	8.5	8.1	8.4	10.7	9.6	10.7	9.5	7.2	6.5	7.2	6.2	10.9	10.7	10.8	10.7	8.2	7.6	7.8	7.4
TYRO3	8.8	7.8	8.9	7.7	7.3	6.2	7.3	5.8	8.4	8.6	8.4	8.6	8.6	7.9	8.4	7.6	6.6	6.0	6.5	5.9	6.4	4.7	6.3	5.1	7.3	6.0	7.3	6.1	7.0	5.7	6.7	5.5
UNQ1940	9.2	7.0	8.9	6.9	6.0	4.8	5.2	4.6	10.1	9.8	10.2	9.9	7.1	5.7	6.8	5.3	5.2	5.8	5.3	5.7	4.4	5.6	4.7	5.4	6.4	6.3	6.4	6.3	4.5	6.0	5.3	5.8

UNQ830	6.0	3.4	5.6	3.3	2.9	2.2	2.5	1.4	7.5	6.2	7.5	6.1	4.9	2.8	4.8	3.1
VBP1	6.8	6.9	6.8	7.0	6.5	6.5	6.8	6.7	6.7	6.9	6.8	6.8	7.0	6.9	6.9	7.1
VCAM1	3.4	5.8	3.0	5.8	6.5	8.8	6.8	8.9	2.2	3.0	2.0	2.3	4.9	7.5	5.5	7.8
VCAN	8.3	8.5	8.0	8.6	8.4	8.3	8.4	8.3	8.0	8.3	7.9	8.1	8.7	9.0	8.7	8.7
WNT10B	4.8	3.8	4.3	3.4	3.9	2.6	3.8	2.9	4.4	5.1	4.1	5.1	6.2	5.0	6.1	4.7
WNT11	8.5	6.8	8.2	6.5	5.9	4.8	5.3	4.6	9.4	9.2	9.4	9.2	8.7	7.4	8.4	7.4
WNT3A	3.2	2.8	3.0	2.9	2.8	2.5	2.9	2.8	4.4	3.7	3.9	3.8	2.8	2.5	2.1	3.0
WNT4	3.8	2.5	3.8	2.2	2.3	2.1	3.0	2.7	4.4	4.0	4.5	4.2	3.8	3.1	2.5	2.8
WNT5A	10.5	10.2	10.4	10.3	11.7	11.3	11.9	11.3	7.9	7.6	7.8	7.6	9.8	9.8	9.9	10.1
WNT5B	8.0	8.5	7.9	8.6	8.5	8.4	8.7	8.4	6.4	7.1	6.4	7.1	7.8	8.1	8.1	8.1
WNT7A	3.4	2.4	3.0	2.3	2.4	1.8	2.8	1.6	4.7	3.4	4.4	3.9	2.7	2.6	2.8	1.1
ZBTB16	6.8	7.0	6.8	6.9	3.3	2.7	3.1	2.8	6.7	6.2	6.3	6.4	3.0	3.4	3.3	1.5
ZNF277	6.4	6.6	6.4	6.3	6.3	6.2	6.5	6.2	6.1	6.0	6.1	6.0	6.2	6.4	5.6	6.4
ZNF521	6.1	6.2	5.6	6.2	5.9	6.1	5.7	6.1	6.1	6.1	5.9	6.3	5.9	6.1	5.2	6.3
ZNF533	6.5	4.9	6.4	4.7	4.0	1.7	3.3	2.2	7.7	7.1	7.7	7.2	6.0	4.0	5.5	3.9

UNQ830	2.9	2.8	3.0	2.4	2.8	2.8	3.2	2.9	4.0	2.4	4.4	2.4	3.6	2.2	2.9	1.8
VBP1	7.0	6.5	7.1	6.7	7.1	6.5	7.2	6.4	7.1	6.4	7.0	6.5	7.4	6.6	7.2	5.7
VCAM1	4.8	6.0	4.1	5.9	5.9	9.2	5.9	9.1	4.4	6.5	4.5	6.3	5.0	9.5	5.3	9.1
VCAN	9.6	9.2	9.3	9.3	10.0	7.3	9.7	7.3	9.7	10.1	9.7	10.0	10.5	8.3	10.7	8.5
WNT10B	2.1	1.2	2.0	1.2	2.4	1.0	2.6	0.8	2.7	2.1	4.3	0.8	2.7	2.9	1.7	2.3
WNT11	3.6	2.9	3.3	3.0	4.1	2.7	3.8	2.9	5.6	3.5	5.8	3.5	5.1	3.6	5.3	4.0
WNT3A	1.1	1.6	1.4	1.6	2.0	1.9	2.6	0.8	0.9	1.1	1.2	1.7	3.3	2.6	2.9	1.8
WNT4	2.1	2.2	2.4	1.6	2.6	1.8	3.5	1.2	2.4	2.2	2.8	2.1	3.0	2.1	3.5	2.4
WNT5A	11.8	9.5	11.8	9.6	12.4	11.3	12.4	11.4	10.1	8.5	10.1	8.4	12.1	10.5	12.2	10.4
WNT5B	7.5	8.1	7.5	8.0	7.0	7.0	6.8	7.1	6.5	7.5	6.2	7.8	7.6	7.1	7.6	7.5
WNT7A	2.8	2.3	2.6	1.8	1.6	2.8	2.0	1.4	2.9	2.4	2.4	2.8	3.4	2.4	3.2	3.5
ZBTB16	8.9	8.7	9.0	8.8	3.5	2.6	3.1	0.2	9.0	9.7	8.7	9.7	3.5	2.7	3.4	1.6
ZNF277	7.3	7.0	7.2	7.0	6.9	6.1	6.8	6.2	7.2	7.7	7.0	7.7	7.4	6.5	7.2	6.3
ZNF521	5.7	5.8	6.0	5.9	6.1	6.5	6.0	6.5	5.9	6.6	5.7	6.3	6.3	6.7	6.2	6.3
ZNF533	2.3	2.4	2.1	2.4	2.4	1.2	3.1	2.7	4.8	3.7	4.1	3.6	3.6	2.0	1.5	1.9
