# Supplementary material for: Analysis of the Effects of Five Factors Relevant to In Vitro Chondrogenesis of Human Mesenchymal Stem Cells Using Factorial Design and High Throughput mRNA-Profiling
Source: PLoS One. 2014 May 9;9(5):e96615. doi: 10.1371/journal.pone.0096615 (PMC4015996; doi:10.1371/journal.pone.0096615)
Supplement: Table S1 — Primary and secondary antibodies used in immunohistochemistry. (DOC) [file pone.0096615.s010.doc]

**Supporting Information Table S1:** List of primary and secondary antibodies used for immunohistochemistry.

| **Antibodies for immunohistochemistry** | Working concentration | Protein | Company | Catalog number |
| --- | --- | --- | --- | --- |
| Monoclonal Mouse IgG1 anti-COL2 | 0.83 mg/mL | Type 2 collagen | MP Biomedicals | Clone II-4C11 / #863171 |
| Monoclonal Mouse IgG1anti-ACAN | 4.55 mg/mL | Aggrecan | Biosource | Clone 969D4D11 /  58.146.21 |
| Monoclonal Mouse IgG2a anti-COL1 | 1 mg/mL | Type I collagen | MP Biomedicals | Clone I-8H5 / #863170 |
| Polyclonal Rabbit anti-SOX9 | 0.2 mg/mL | SRY-box containing gene 9 | Millipore | AB5535 |
| Alexa488-conjugated goat anti-rabbit | 5 mg/mL |  | Invitrogen |  |
| Cy3-conjugated donkey anti-mouse IgG | 2 mg/mL |  | Jackson Immuno Research |  |
