# Supplementary material for: Analysis of the Effects of Five Factors Relevant to In Vitro Chondrogenesis of Human Mesenchymal Stem Cells Using Factorial Design and High Throughput mRNA-Profiling
Source: PLoS One. 2014 May 9;9(5):e96615. doi: 10.1371/journal.pone.0096615 (PMC4015996; doi:10.1371/journal.pone.0096615)
Supplement: Table S2 — Statistical response analysis of main effects and second and third order interactions. (DOCX) [file pone.0096615.s011.docx]

**Supporting Information Table S2: Statistical response analysis of main effects and second and third order interactions at day 1 and 7.**

| **Factorial Fit Of Wanted Genes versus All Five Factors Including Two-Factor Interactions on Day 7** | | | | | |
| --- | --- | --- | --- | --- | --- |
| **Term** | **Effect** | | **Coef** | **T** | **P** |
| Constant |  | | 9.3892 | 110.81 | **0.000** |
| TGFb1 | 3.3493 | | 1.6746 | 19.76 | **0.000** |
| FGF2 | -1.3188 | | -0.6594 | -7.78 | **0.000** |
| DEX | 1.9587 | | 0.9794 | 11.56 | **0.000** |
| IGF1 | 0.0902 | | 0.0451 | 0.53 | 0.596 |
| BMP2 | 1.5581 | | 0.7791 | 9.19 | **0.000** |
| TGFb1*FGF2 | -0.3273 | | -0.1637 | -1.93 | 0.057 |
| TGFb1*DEX | 1.1082 | | 0.5541 | 6.54 | **0.000** |
| TGFb1*IGF1 | 0.0979 | | 0.0489 | 0.58 | 0.565 |
| TGFb1*BMP2 | -0.4874 | | -0.2437 | -2.88 | **0.005** |
| FGF2*DEX | 0.0864 | | 0.0432 | 0.51 | 0.612 |
| FGF2*IGF1 | 0.0052 | | 0.0026 | 0.03 | 0.976 |
| FGF2*BMP2 | -0.1035 | | -0.0517 | -0.61 | 0.543 |
| DEX*IGF1 | -0.1022 | | -0.0511 | -0.6 | 0.548 |
| DEX*BMP2 | 0.2296 | | 0.1148 | 1.35 | 0.180 |
| IGF1*BMP2 | 0.0462 | | 0.0231 | 0.27 | 0.786 |
| TGFb1*DEX*BMP2 | -0.3821 | | -0.1911 | -2.25 | **0.027** |
| R^2^ = 91.32% |  | | |  | |
|  |  | | |  | |
| **Factorial Fit Of Unwanted Genes versus All Five Factors Including Two-Factor Interactions on Day 7** | | | | | |
| **Term** | **Effect** | **Coef** | | **T** | **P** |
| Constant |  | 10.8056 | | 225.62 | 0.000 |
| TGFb1 | 1.0835 | 0.5418 | | 11.31 | 0.000 |
| FGF2 | -0.2662 | -0.1331 | | -2.78 | 0.007 |
| DEX | 0.1495 | 0.0747 | | 1.56 | 0.123 |
| IGF1 | -0.0217 | -0.0109 | | -0.23 | 0.821 |
| BMP2 | 1.0365 | 0.5183 | | 10.82 | 0.000 |
| TGFb1*FGF2 | 0.0332 | 0.0166 | | 0.35 | 0.730 |
| TGFb1*DEX | -0.1856 | -0.0928 | | -1.94 | 0.057 |
| TGFb1*IGF1 | 0.0361 | 0.018 | | 0.38 | 0.707 |
| TGFb1*BMP2 | -0.9729 | -0.4865 | | -10.16 | 0.000 |
| FGF2*DEX | 0.2545 | 0.1272 | | 2.66 | 0.010 |
| FGF2*IGF1 | -0.0015 | -0.0007 | | -0.02 | 0.988 |
| FGF2*BMP2 | 0.148 | 0.074 | | 1.54 | 0.127 |
| DEX*IGF1 | 0.0188 | 0.0094 | | 0.2 | 0.845 |
| DEX*BMP2 | -0.059 | -0.0295 | | -0.62 | 0.540 |
| IGF1*BMP2 | 0.0155 | 0.0078 | | 0.16 | 0.872 |
| TGFb1*FGF2*DEX | 0.2719 | 0.1359 | | 2.84 | **0.006** |
| R^2^= 84.67% |  | | |  | |

| **Factorial Fit Of Wanted Genes versus All Five Factors Including Two-Factor Interactions on Day 1** | | | | | |
| --- | --- | --- | --- | --- | --- |
| **Term** | | **Effect** | **Coef** | **T** | **P** |
| Constant | |  | 7.5912 | 218.76 | **0.000** |
| TGFb1 | | 0.5807 | 0.2904 | 8.37 | **0.000** |
| FGF2 | | -0.4061 | -0.2031 | -5.85 | **0.000** |
| DEX | | 0.4144 | 0.2072 | 5.97 | **0.000** |
| IGF1 | | -0.0429 | -0.0214 | -0.62 | 0.540 |
| BMP2 | | 0.735 | 0.3675 | 10.59 | **0.000** |
| TGFb1*FGF2 | | -0.0912 | -0.0456 | -1.31 | 0.196 |
| TGFb1*DEX | | 0.1099 | 0.0549 | 1.58 | 0.122 |
| TGFb1*IGF1 | | -0.0528 | -0.0264 | -0.76 | 0.452 |
| TGFb1*BMP2 | | -0.3338 | -0.1669 | -4.81 | **0.000** |
| FGF2*DEX | | -0.0326 | -0.0163 | -0.47 | 0.641 |
| FGF2*IGF1 | | -0.022 | -0.011 | -0.32 | 0.753 |
| FGF2*BMP2 | | -0.1376 | -0.0688 | -1.98 | 0.055 |
| DEX*IGF1 | | -0.0121 | -0.0061 | -0.17 | 0.862 |
| DEX*BMP2 | | 0.0698 | 0.0349 | 1.01 | 0.321 |
| IGF1*BMP2 | | 0.0409 | 0.0205 | 0.59 | 0.559 |
| R^2^= 88.38 % | |  | |  | |
|  | |  | |  | |
| **Factorial Fit Of Unwanted Genes versus All Five Factors Including Two-Factor Interactions on Day 1** | | | | | |
| **Term** | **Effect** | | **Coef** | **T** | **P** |
| Constant |  | | 9.5693 | 337.77 | **0.000** |
| TGFb1 | 0.1489 | | 0.0745 | 2.63 | **0.012** |
| FGF2 | -0.2385 | | -0.1193 | -4.21 | **0.000** |
| DEX | -0.0565 | | -0.0283 | -1 | 0.325 |
| IGF1 | -0.0138 | | -0.0069 | -0.24 | 0.808 |
| BMP2 | 0.2797 | | 0.1398 | 4.94 | **0.000** |
| TGFb1*FGF2 | -0.0746 | | -0.0373 | -1.32 | 0.196 |
| TGFb1*DEX | -0.0899 | | -0.0449 | -1.59 | 0.121 |
| TGFb1*IGF1 | 0.0007 | | 0.0004 | 0.01 | 0.990 |
| TGFb1*BMP2 | -0.1963 | | -0.0981 | -3.46 | **0.001** |
| FGF2*DEX | 0.0284 | | 0.0142 | 0.5 | 0.619 |
| FGF2*IGF1 | 0.0123 | | 0.0061 | 0.22 | 0.830 |
| FGF2*BMP2 | -0.0437 | | -0.0218 | -0.77 | 0.445 |
| DEX*IGF1 | -0.0155 | | -0.0078 | -0.27 | 0.786 |
| DEX*BMP2 | -0.0321 | | -0.0161 | -0.57 | 0.574 |
| IGF1*BMP2 | 0.0536 | | 0.0268 | 0.95 | 0.350 |
| R^2^= 65.67% |  | | |  | |

R^2^ represent the proportion of variation in the response data that is explained by the fitted mathematical model.
