# Supplementary material for: Analysis of the Effects of Five Factors Relevant to In Vitro Chondrogenesis of Human Mesenchymal Stem Cells Using Factorial Design and High Throughput mRNA-Profiling
Source: PLoS One. 2014 May 9;9(5):e96615. doi: 10.1371/journal.pone.0096615 (PMC4015996; doi:10.1371/journal.pone.0096615)
Supplement: Table S3 — Significantly regulated genes uniformly up- or downregulated by DEX. (DOCX) [file pone.0096615.s012.docx]

**Supporting Information Table S3:** List of significantly regulated genes uniformly up- or downregulated by DEX in the presence of TGFb1 alone or in combination with BMP2 corresponding to the overlap in the Venn-diagram in Supporting Information Figure S6.

| **Down** |  | **Up** |  |  |  |
| --- | --- | --- | --- | --- | --- |
| ARHGEF3 | LGALS3BP | ACAN | CXCL14 | NOS2 | TRPV4 |
| CASP4 | LUM | ANG | FMOD | NPAS2 | TSC22D3 |
| CD44 | MMP13 | ANGPTL7 | FOXA2 | PCOLCE2 | TSC22D4 |
| CDH11 | NOTCH3 | APOD | HAPLN1 | PGK1 | TUBB2A |
| CDKN2B | PBX1 | BDKRB1 | HPRT1 | PMP2 | TUBB2B |
| COL18A1 | RUNX1 | C1QTNF3 | IL13RA1 | S100P | UNQ1940 |
| COL3A1 | RUNX2 | CA9 | IL20RB | SCIN | UNQ830 |
| CXCL12 | SFRP4 | CD109 | IRF6 | SCRG1 | ZBTB16 |
| EPHA2 | SMAD7 | CDKN1C | KLF15 | SERPINA3 |  |
| FGFR1 | TBX3 | COL11A1 | MATN3 | SFRP1 |  |
| FOXO1 | TGFB3 | COL2A1_iso2 | MMP7 | SHC4 |  |
| FZD8 | TIMP2 | COL9A1 | MST4 | SOX8 |  |
| GLI3 | VCAM1 | COL9A3 | MYC | SOX9 |  |
| JAG1 | WNT5A | CTGF | NFIL3 | TIMP4 |  |
